# CuPCA: a web server for pan-cancer association analysis of large-scale cuproptosis-related genes

**DOI:** 10.1093/database/baae075

**Published:** 2024-09-04

**Authors:** Yishu Xu, Zhenshu Ma, Yajie Wang, Long Zhang, Jiaming Ye, Yuan Chen, Zhengrong Yuan

**Affiliations:** College of Biological Sciences and Technology, Beijing Forestry University, Beijing 100083, China; College of Computer Science and Technology, Beijing Forestry University, Beijing 100083, China; Department of Clinical Laboratory, Beijing Ditan Hospital, Capital Medical University, Beijing 100015, China; College of Art, Beijing Forestry University, Beijing 100083, China; College of Biological Sciences and Technology, Beijing Forestry University, Beijing 100083, China; Department of Clinical Laboratory, Beijing Ditan Hospital, Capital Medical University, Beijing 100015, China; College of Biological Sciences and Technology, Beijing Forestry University, Beijing 100083, China

## Abstract

Copper-induced cell death is a novel mechanism of cell death, which is defined as cuproptosis. The increasing level of copper can produce toxicity in cells and may cause the occurrence of cell death. Several previous studies have proved that cuproptosis has a tight association with various cancers. Thus, the discovery of relationships between cuproptosis-related genes (CRGs) and human cancers is of great importance. Pan-cancer analysis can efficiently help researchers find out the relationship between multiple cancers and target genes precisely and make various prognostic analyses on cancers and cancer patients. Pan-cancer web servers can provide researchers with direct results of pan-cancer prognostic analyses, which can greatly improve the efficiency of their work. However, to date, no web server provides pan-cancer analysis about CRGs. Therefore, we introduce the cuproptosis pan-cancer analysis database (CuPCA), the first database for various analysis results of CRGs through 33 cancer types. CuPCA is a user-friendly resource for cancer researchers to gain various prognostic analyses between cuproptosis and cancers. It provides single CRG pan-cancer analysis, multi-CRGs pan-cancer analysis, multi-CRlncRNA pan-cancer analysis, and mRNA–circRNA–lncRNA conjoint analysis. These analysis results can not only indicate the relationship between cancers and cuproptosis at both gene level and protein level, but also predict the conditions of different cancer patients, which include their clinical condition, survival condition, and their immunological condition. CuPCA procures the delivery of analyzed data to end users, which improves the efficiency of wide research as well as releases the value of data resources.

**Database URL**: http://cupca.cn/

## Introduction

Copper is a crucial trace element necessary for cardiovascular, neural, and immune system functions [1]. It plays a significant supportive role in various life processes by maintaining homeostasis [2]. Copper-dependent cell death occurs when copper directly binds to lipoylated components of the tricarboxylic acid (TCA) cycle [3, 4]. This can lead to various types of cell death, such as apoptosis and autophagy, primarily through mechanisms like antiangiogenesis, proteasome inhibition, and accumulation of reactive oxygen species [5]. This unique type of copper-induced regulated cell death is called cuproptosis [6]. Cuproptosis is related to cancers since unbalanced Cu homeostasis can affect tumor growth and may cause irreversible damage to cancer cells. Thus, cuproptosis can be used for developing effective cancer therapies, and the expression of CRGs is of great importance in cancer therapy research. Pan-cancer studies have identified many genes that are frequently somatically altered across multiple tumor types, suggesting that pathway-targeted therapies can be deployed across diverse cancers [7, 8].

Pan-cancer analysis can help researchers discover the relationship between multiple cancers and target genes precisely and efficiently. Some databases have provided gene data for tumor and normal samples through various cancer types, such as The Cancer Genome Atlas (TCGA, https://portal.gdc.cancer.gov/) and the exoRBase database (http://www.exorbase.org/). The data from these databases are downloaded and become the resource that we need, and there are various analyses that we can carry out through them, such as differential expression analysis, clinical analysis, correlation analysis, patient survival analysis, immune function analysis, and conjoint analysis. With the use of pan-cancer analysis, web servers can provide direct and convenient information to cancer researchers, which may help improve their experimental progress. For example, the Ferritin Database [9] (FerrDb) is a database for markers and regulators of ferroptosis and ferroptosis–disease associations. Gene Expression Profiling Interactive Analysis [10] (GEPIA) database provides users with the differential expression conditions of certain genes between cancer patients and normal people. However, no web server has provided a pan-cancer analysis about CRGs until now. Therefore, we introduce CuPCA, the first database that works for CRGs. The CuPCA website is freely available to all visitors.

## Materials and methods

### Acquisition of data

**Figure 1.**
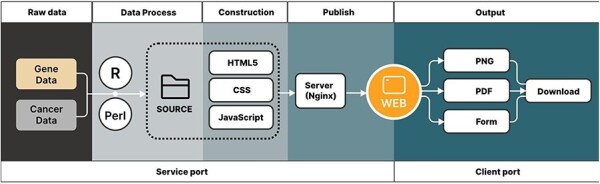
Outlines show the acquisition of data and data output for the CuPCA visualization tools.

CRG-related articles were searched and selected from online libraries, such as PubMed and Web of Science. These articles have shown their discovery through genes related to cuproptosis, and then 1187 CRGs (Supplementary Table S1) were selected for the use of our pan-cancer analysis. The gene data and cancer data source of the article were downloaded from the TCGA and GTEx databases (Fig. 1). These raw data were divided into the tumor and normal groups, which were most suitable for comparisons.

### Construction of CuPCA

After finishing the organization of raw data, the pan-cancer analysis was processed using R script (version 4.2.1) and Perl (version 5.30.0.1) (Fig. 1). CuPCA used HyperText Markup Language 5 (HTML5) and Cascading Style Sheets (CSS) to build the basic framework of the web page, including the navigation bar, search box, etc. (Fig. 1). JavaScript was used to realize the presentation and the switching of the search mechanism and background chart (Fig. 1). All of the prognostic analysis results that CuPCA provides are stored on the lightweight cloud server, while the web server uses the popular Nginx, which is opened to implement website publishing after setting the pat. The web traffic is monitored based on web page background user data, and the safety of website operation is ensured through the cloud server anti-Distributed Denial of Service (DDOS) service (Fig. 1). Users can obtain analysis results on the website by browsing or downloading them. The formats of the analysis results include and are not limited to PNG (Portable Network Graphics), PDF (Portable Document Format), and Table (Fig. 1). The website can change its look and feel automatically according to different browsers and devices, ranging from desktop computers to tablets and smartphones.

### Functionalities

#### Quick start

**Figure 2.**
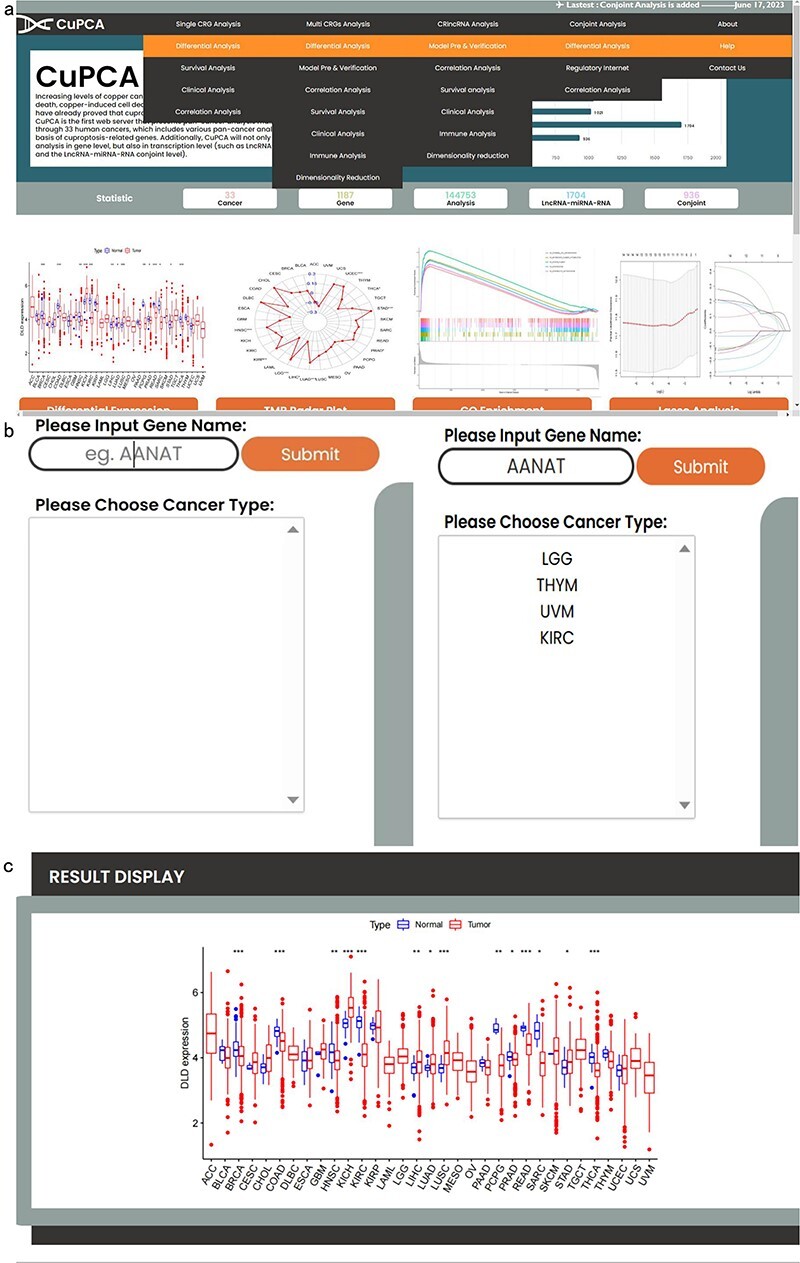
Top navigation of CuPCA functions and the initial interface of CuPCA. (a) The total menu of CuPCA. (b) The search bar of CuPCA. (c) The example of a pan-cancer analysis result presented by CuPCA.

CuPCA provides a convenient search interface. Since we have different kinds of analysis (Fig. 2a), users can choose the type of analysis they want and then put a gene symbol (e.g. *AANAT*) in the “Gene Name” option or their interested cancer type in the “Please Choose Cancer Type” option (Fig. 2b) to search for the various analysis results (Fig. 2c). For example, Fig. 2c presents the differential expression analysis result of the dihydrolipoamide dehydrogenase (*DLD*) gene in the single CRG analysis part.

## Results

### Pan-cancer analysis of a single CRG

In this unit, CuPCA provides pan-cancer analysis based on every single CRG (Supplementary Table S1) and cancers (Supplementary Table S2), which involve 1187 genes and 33 cancers in total. Users can search for the name of the target gene to gain the pan-cancer analysis that they want. Association analysis results of CRGs are divided into four categories, namely, differential analysis, survival analysis, clinical analysis, and correlation analysis categories.

### Differential analysis

**Figure 3.**
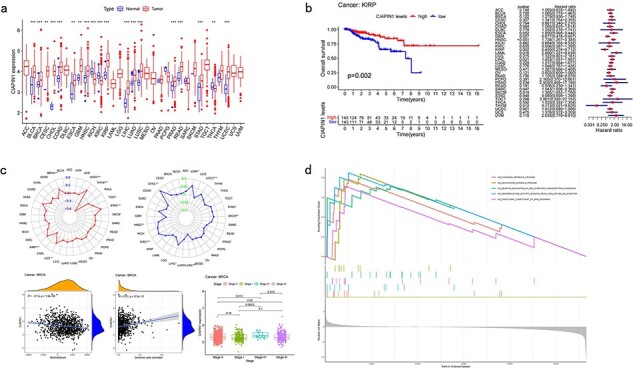
Pan-cancer analysis of single CRG. (a) Differential analysis. (b) Survival analysis. (c) Clinical analysis. (d) Correlation analysis.

Differential analysis (Fig. 3a) profiles show users the differential expression of each CRG in tumor and normal samples. Users can also find precise *P*-values in the profile to know the significance of differentiation.

### Survival analysis

Survival analysis (Fig. 3b) shows the influence that each CRG has on cancer patients, which includes the overall survival analysis (OS), disease-specific survival analysis (DSS), progression-free interval analysis (PFI), and disease-free interval analysis (DFI). The profiles include both the Kaplan–Meier (KM) analysis results and the Cox analysis results [11]. Survival analysis can determine which CRG has an influence on cancer patients and the degree of their influence, which includes the time and possibility that they can survive in the future.

### Clinical analysis

There are various correlation analyses in this part, which include tumor mutational burden (TMB), microsatellite instability (MSI), estimate correlation (EC), CIBERSORT correlation (CC), and clinical correlation analysis (CCA) (Fig. 3c). Both the analyses show the correlation between cancers, CRGs, and some clinical characteristics. TMB analysis and MSI analysis indicate the relationship between the expression of CRG and each cancer’s TMB score and MSI score. EC and CC analyses infer the relationship between the expression of CRG and each cancer’s microenvironment, and the content of the immune cell, respectively. CCA shows users the correlation between CRG’s expression and different clinical groups.

### Correlation analysis

At the gene level, CuPCA provides the Gene Set Enrichment Analysis (GSEA), which includes the Kyoto Encyclopedia of Genes and Genomes (KEGG) and Gene Ontology (GO) analysis. This analysis points out the relationship between target genes, certain immune functions, and some cell pathways (Fig. 3d).

### Pan-cancer analysis of multi-CRGs

In this unit, CuPCA provides users with a pan-cancer analysis of all the most related or most important CRGs. CuPCA downloaded the transcriptomic data of 143 CRGs (Supplementary Table S3) from the TCGA database and regarded the expression data (mRNA data) as the training group. Meanwhile, data that gained from the Gene Expression Omnibus (GEO) database were obtained from the NCBI website (https://www.ncbi.nlm.nih.gov/), and they were regarded as the test group. All patients were divided into a high-risk group and a low-risk group according to their risk scores. A prognostic model [12,13] was built based on the two groups for each cancer. The prognostic models were presented with a formula, which is shown as follows:


$$Risk\;Score = \sum\limits_{i = 1}^n {Expi \times CRG}\\[4pt], $$

where Exp*_i_* means the expression of each CRG. CRG means each prognostic gene’s correlation coefficients, i.e. regression coefficients for each feature (gene) in the model. This formula was used to calculate each patient’s risk score. Due to the lack of certain cancers’ GEO data, CuPCA can only present 11 cancer models. CuPCA provides users with both the details of the model and various prognostic analyses, and users can gain information by searching their target cancer.

### Differential analysis

**Figure 4.**
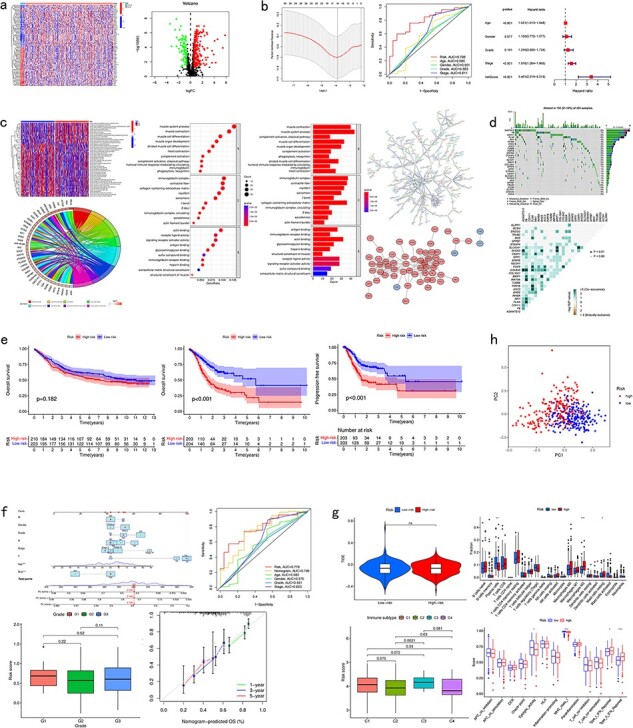
Pan-cancer analysis of multi-CRGs. (a) Differential analysis. (b) Presentation of the prognostic model and its verification results. (c) Correlation analysis. (d) Mutation analysis. (e) Survival analysis. (f) Clinical analysis. (g) Immune analysis. (h) PCA analysis.

CuPCA gives out a differential gene heatmap and a volcano plot, which presents users with the different expression conditions of each CRG in certain cancers (Fig. 4a).

### Model presentation and verification

For the model presentation and verification, CuPCA shows users both the information about the models and the verification of the model’s prognosis ability. For the model itself, CuPCA presents the model formula, the result of prognostic gene prediction, the Least Absolute Shrinkage and Selection Operator (LASSO) Regression analysis, and the Cox analysis. For verification, CuPCA provides users with the results of the independent prognostic analysis (IPA) and the receiver operating characteristic (ROC) curve (Fig. 4b). The ROC curve can indicate the accuracy of our prognostic model, since the larger the area under the ROC curve, the stronger the ability of the model to predict patient survival conditions [14,15].

### Correlation analysis

As for the correlation analysis, CuPCA provides various correlation analysis results, which include the Gene Set Variation analysis (GSVA), GO and KEGG enrichment analysis, Protein–Protein Interaction (PPI), and Cytoscape visualization (Fig. 4c). They show users the relationship between gene and immune functions, gene and pathways, and protein and protein.

### Mutation analysis

As for the mutation analysis, CuPCA provides a Prognostic Gene Waterfall Plot, which can present the mutation frequency of those genes in different patients’ samples (Fig. 4d).

### Survival analysis

In this unit, CuPCA provides users with OS analysis and progression-free survival (PFS) analysis. The OS analysis has two plots in total, which are the survival analyses of the TCGA data and the GEO data, respectively. The PFS analysis has only one plot, which presents the survival analysis of the TCGA data (Fig. 4e).

### Clinical analysis

For the clinical analysis, CuPCA presents users with the clinical correlation analysis (Fig. 4f) and the Nomogram [16]. The Nomogram is presented with its ROC curve and its independent prognostic analysis (Fig. 4f).

### Immune analysis

Several kinds of immune analysis are provided by CuPCA in this part, which includes the results of immunotyping analysis, immune cell differential analysis, immune-related functions analysis, and immunotherapy analysis. This may help users gain treatment thoughts for certain cancers (Fig. 4g). Since immunotyping analysis determines the difference in patients’ risk scores in various immune groups, immune cell differential analysis, and immune-related functions analysis show the relationship between immune cells and different patient groups, and the relationship between immune-related functions and different patient groups. Immunotherapy predicts the therapeutic effects of drugs on different tumor patients.

### Dimensionality reduction

CuPCA provides the results of the principle component analysis (PCA) [17] for a given gene list and sample dataset in each cancer. It shows users whether the prognostic model can distinguish the high-risk patients and low-risk patients (Fig. 4h).

### Pan-cancer analysis of CRlncRNA

Some lncRNA has a relationship with cuproptosis and human cancers [18–23]. We did various prognostic analyses on these CRlncRNA, too. We downloaded the transcriptomic data through 33 cancers from the TCGA database, and the lncRNA data were selected from it. Due to the insufficient raw data, CuPCA only provides 13 human cancer analyses in total. Since the number of lncRNAs that are related to all CRGs is large, the analysis of those is meaningless. So CuPCA firstly selected lncRNA (named CRlncRNA) that has a relationship with 10 core CRGs, which were *CDKN2A*, *FDX1*, *DLD*, *DLAT, LIAS*, *GLS*, *LIPT1*, *MTF1*, *PDHA*, and *PDHB* [3]. LASSO analysis performs variable selection and dimensionality reduction by adding a penalty term to reduce model overfitting, while COX analysis evaluates the relationship between a single variable and survival time to identify potential influencing factors [24–26]. Thus, CuPCA secondly carried out LASSO analysis and single-factor Cox analysis on them to choose the lncRNA that is most related to the prognostic process. At last, CuPCA gained the CRlncRNA that is highly related to cuproptosis through multi-factor Cox analysis and made a prognostic model based on them. Thus, each cancer has its most related CRlncRNA and a prognostic model. Users can choose their target cancer, and the analysis based on the model will be shown. There are seven kinds of analyses in total, which are model presentation and verification, correlation analysis, mutation analysis, survival analysis, clinical analysis, immune analysis, and dimensionality reduction.

### Model presentation and verification

**Figure 5.**
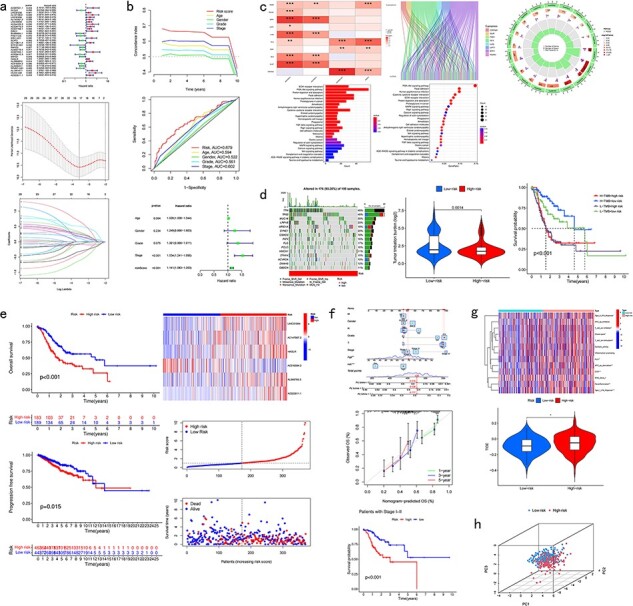
Pan-cancer analysis of CRlncRNA. (a) Presentation of the prognostic model. (b) Verification results of the prognostic model. (c) Correlation analysis. (d) Mutation analysis. (e) Survival analysis. (f) Clinical analysis. (g) Immune analysis. (h) PCA analysis.

CuPCA has a prognostic model for each cancer. The CRlncRNA samples were divided into two groups, which were the training group and the test group. According to these two groups, a prognostic model was made. In the presentation part, CuPCA provided the model’s formula, the single-factor forest plot, and the LASSO analysis result of the model (Fig. 5a). For the verification part, CuPCA provides the C-index curve, the ROC curve, and the independent prognostic analysis (Fig. 5b) to visualize the prognostic model’s ability to calculate the risk score of samples [27]. The formula is presented as follows:


$$Risk\;Score = \sum\limits_{i = 1}^n {Expi \times LncRNA}, $$

where Exp_i_ means the expression of each lncRNA and lncRNA means each prognostic lncRNA’s correlation coefficients, i.e. regression coefficients for each feature (lncRNA) in the model.

### Correlation analysis

This function can help users find out the relationship between lncRNA and CRGs in each cancer, the correlation between the prognostic analysis and the clinical factors, and the relationship between lncRNA and the pathway or functionality of every cancer. Due to the limitation of raw data in TCGA, not all types of cancers can be put into the correlation analysis part. CuPCA provides users with various correlation analyses that include the co-expression analysis, correlation heatmap, Sanger plot, GO, and KEGG enrichment analysis (Fig. 5c). It is worth mentioning that CuPCA has made the co-expression analysis an interaction chart. Users can get direct, precise correlation details between target CRlncRNA and CRGs by searching the name of the target lncRNA or CRG, which includes the correlation coefficient, *P*-value, and their regulation relationship.

### Mutation analysis

CuPCA carries out tumor mutation load score, tumor mutation load differential analysis, and tumor mutation burden survival analysis (Fig. 5d). Besides, CuPCA provides visualization results of the frequency and type of mutations, which is called the Waterfall plot (Fig. 5d).

### Survival analysis

CuPCA provides several kinds of survival analysis of CRlncRNA, which include OS Analysis, PFS Analysis, and RiskPlot (Fig. 5e).

### Clinical analysis

CuPCA presents the model validation, which can be used to identify whether our model is suitable for patients in different stages. CuPCA also shows users the Nomogram that can tell users about the prediction of patients’ survival (Fig. 5f).

### Immune analysis

This function helps users gain analysis results of immune-related functions analysis, immune evasion and immunotherapy analysis results (Fig. 5g). Immune evasion and immunotherapy analysis [28] can help users compare the effects of patients receiving immunotherapy in high- and low-risk groups.

### Dimensionality reduction

PCA [29,30] analysis reduces the dimensionality of data while maximizing the retention of the original data’s variance by transforming the data into a new coordinate system. For a given gene list and sample dataset, CuPCA provides PCA analysis results of each tumor (Fig. 5h). It provides users the accurcy of our prognostic model, by comparing the prognostic lncRNA’s ability to distinguish the high-risk patients and low-risk patients with the other CRlncRNA, lncRNA, CRGs, and all genes.

### Pan-cancer conjoint analysis of cuproptosis-related mRNA–lncRNA–circRNA

CuPCA provides users with conjoint analysis based on mRNA, lncRNA, and circRNA. The data of these were downloaded from the exoRBase database (http://www.exorbase.org/). They became linked through their competition for miRNA, CuPCA visualized the interaction of them by making it a regulatory internet.

### Differential analysis

**Figure 6.**
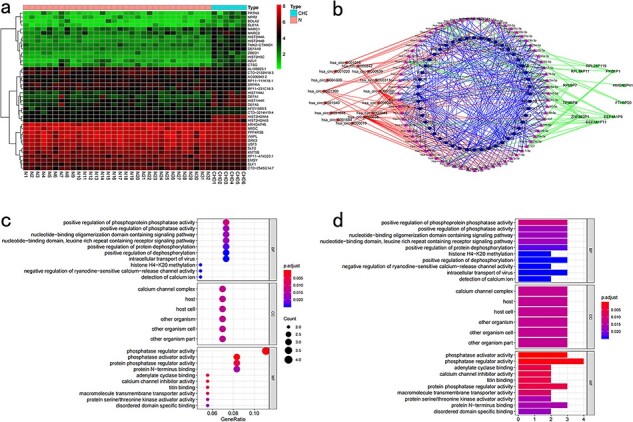
Pan-cancer conjoint analysis of mRNA–lncRNA–circRNA. (a) Differential analysis. (b) Regulatory internet. (c) KEGG enrichment analysis. (d) GO enrichment analysis.

CuPCA presents users with a differential analysis of mRNA, lncRNA, and circRNA. According to the differential analysis chart, the heatmap was prepared (Fig. 6a). These analyses show users the different expressions of cuproptosis-related mRNA, lncRNA, and circRNA between tumors and normal tissues.

### Regulatory internet

mRNA, lncRNA, and circRNA have a relationship with each other through the binding of miRNA, and miRNA is the bridge that brings them together. Thus, in this part, CuPCA shows users the binding condition of miRNA and mRNA, miRNA and lncRNA, and miRNA and circRNA. According to the binding condition presented in the part above, a regulatory network was created. It is a visualization of the miRNA-binding condition, from which the users can gain the competition results between miRNA and mRNA, miRNA and lncRNA, and miRNA and circRNA (Fig. 6b).

### Correlation analysis

CuPCA provides users with the results of GO and KEGG analyses (Fig. 6c and d), which indicate the enrichment condition of genes in certain functions and pathways.

## Discussion

The way copper induces tumor cell death is vital for research targeting the discovery of various cancer therapies. With the help of pan-cancer analysis, CuPCA can carry out clear results about the influence of CRGs on cancers. Databases such as TCGA and GTEx have provided CuPCA with a tremendous amount of gene data through 33 cancer types and their clinical information. While some websites have shown some gene expression profiling and interactive analyses, many other types of gene analyses that may be useful for cancer treatment are still not carried out. For example, websites such as GEPIA provide differential analysis of genes but have no specific *P*-value, which means that it cannot distinguish between the *P*-value of 0.05, 0.01, and 0.001 range, neither fold-change nor gene rank. Moreover, survival analysis on the other websites is relatively simple, with too little clinical information. CuPCA has added immune function analysis such as immune escape and immunotherapy analysis to help researchers have a prediction for the effect of their cancer treatment. To sum up, the most important characteristic of CuPCA is that it is the first database related to cuproptosis. Researchers who are interested in this field can use the data provided by CuPCA to find out biomarkers, highly related genes or lncRNA, the regulatory network of cuproptosis-related mRNA, lncRNA, and circRNA, and proper drugs forward various cancers as well as new ways of disease treatment.

In conclusion, CuPCA has worked out 1187 gene analyses through 33 cancer types in total. CuPCA is dedicated to helping the work of cancer researchers, which includes reducing their experiment time and providing cancer therapy ideas. CuPCA database is freely available to all visitors. CuPCA will not only be served for cuproptosis-related pan-cancer analysis in the future, but also new human genes’ relative analysis results will be added.

## Supplementary Material

baae075_Supp

## Data Availability

The CuPCA database along with the detailed documentation (handbook) is available at http://cupca.cn/Page_5_1_1/.
