# Supplementary material for: CuPCA: a web server for pan-cancer association analysis of large-scale cuproptosis-related genes
Source: Database (Oxford). 2024 Sep 4;2024:baae075. doi: 10.1093/database/baae075 (PMC11373563; doi:10.1093/database/baae075)
Supplement: baae075_Supp [file baae075_supp.zip › suppl_data/Handbook.docx]

## Handbook

### About

Copper-induced cell death is a new type of cell death, which is different from other programmed cell death (such as apoptosis and necrosis) and is defined as cuproptosis. Previous studies have proved that cuproptosis is closely related to various cancers. Therefore, finding the relationship between genes and cancers is of great significance for cancer treatment. Pan-cancer analysis can help researchers effectively discover the similarities and differences of target genes in various aspects of different cancer types as well as the relationship between target genes and cancers. The Cuproptosis Pan-cancer Analysis (CuPCA) database was created to fulfill this purpose.

CuPCA is the first manually curated database that provides researchers with a pan-cancer analysis of cuproptosis-related genes (CRGs). CuPCA provides various prognostic analyses of CRGs and human cancers at both the gene level, transcription level, and the mRNA-lncRNA-circRNA conjoint level. All analyses are provided straightly in the database.

CuPCA is regularly updated to ensure continuous support for its long-term services. The data within CuPCA is freely available for download and is exclusively intended for academic and research purposes.

### Data Structure

Pan-cancer analysis can help researchers discover the relationship between multiple cancers and target genes precisely and efficiently. We collected raw data from tumor and normal samples through various cancer types from public databases, such as The Cancer Genome Atlas (TCGA, https://portal.gdc.cancer.gov/[)](https://www.cancer.gov/ccg/research/genome-sequencing/tcga)) and exoRBase (http://www.exorbase.org/).

### Abbreviations for cancers

| **Cancer Name** | **Description** |
| --- | --- |
| ACC | Adrenocortical carcinoma |
| BLCA | Bladder Urothelial Carcinoma |
| BRCA | Breast invasive carcinoma |
| CESC | Cervical squamous cell carcinoma and endocervical adenocarcinoma |
| CHOL | Cholangiocarcinoma |
| COAD | Colon adenocarcinoma |
| DLBC | Lymphoid Neoplasm Diffuse Large B-cell Lymphoma |
| ESCA | Esophageal carcinoma |
| GBM | Glioblastoma multiforme |
| HNSC | Head and Neck squamous cell carcinoma |
| KICH | Kidney Chromophobe |
| KIRC | Kidney renal clear cell carcinoma |
| KIRP | Kidney renal papillary cell carcinoma |
| LAML | Acute Myeloid Leukemia |
| LGG | Brain Low Grade Glioma |
| LIHC | Liver hepatocellular carcinoma |
| LUAD | Lung adenocarcinoma |
| LUSC | Lung squamous cell carcinoma |
| MESO | Mesothelioma |
| OV | Ovarian serous cystadenocarcinoma |
| PAAD | Pancreatic adenocarcinoma |
| PCPG | Pheochromocytoma and Paraganglioma |
| PRAD | Prostate adenocarcinoma |
| READ | Rectum adenocarcinoma |
| SARC | Sarcoma |
| SKCM | Skin Cutaneous Melanoma |
| STAD | Stomach adenocarcinoma |
| TCGT | Testicular Germ Cell Tumors |
| THCA | Thyroid carcinoma |
| THYM | Thymoma |
| UCEC | Uterine Corpus Endometrial Carcinoma |
| UCS | Uterine Carcinosarcoma |
| UVM | Uveal Melanoma |

### Single CRG Analysis

### Differential Analysis

Under the Single CRG Analysis drop-down, click Differential Analysis to open and use the analysis. Users can gain profiles about the differential expression of target genes in tumor and normal samples and can find precise *P-*values in the picture to know the significance of differentiation. Differential analysis is done based on a single gene’s differential expression on 33 cancers(Figure 1). Users can only get the analysis result after putting the right target gene’s name into the ‘gene name’ input panel, and there is a search box beside the input panel that may help to imply users the name of target genes(Figure 2).

As shown in Figure 1, the horizontal axis represents the different cancer types, blue for Normal and red for Tumor. The vertical axis indicates the expression levels of the studied genes. Normally, each tissue corresponds to two box lines, but if no transcriptome testing was performed or if there is a lack of paracancerous tissue samples, there is only one box line. The upper asterisk (*) indicates the significance of the difference between normal and tumor tissues.


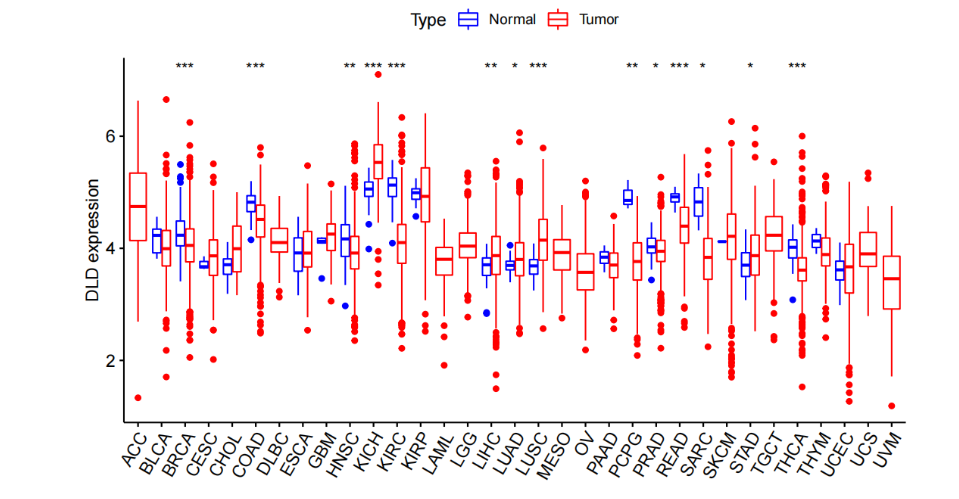


Figure 1. Differential Analysis of a single CRG.


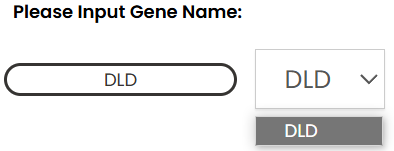


Figure 2. Target gene searching bar in the differential analysis.

### Survival Analysis

Under the Single CRG Analysis drop-down, click Survival Analysis to open and use the analysis. Within the Survival Analysis section, researchers can access a range of sub-analyses for a thorough investigation:

1. Overall Survival Analysis(OS). *The prediction of patients’ survival condition in the OS period.*
2. Disease Specific Survival Analysis(DSS). *The prediction of patients’ survival condition in the DSS period.*
3. Progression-Free Interval Analysis(PFI). *The prediction of patients’ survival condition in the PFI period.*
4. Disease-Free Interval Analysis(DFI). *The prediction of patients’ survival condition in the DFI period.*

Since each gene has a different influence on each cancer, CuPCA only provides survival analysis on the cancers that can be significantly(*P* < 0.05) influenced by the CRG. Users should input the target gene, and choose the target cancer type from the slider bar below that provides the cancers which can be influenced by the target gene(Figure 3). After choosing the target cancer type, users can get the final analysis result of this part(Figure 4). Otherwise, users will not be provided with the result.


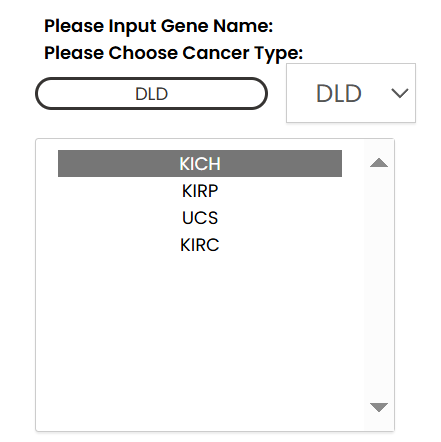


Figure 3. Target gene and target cancer searching bar in the survival analysis.

As shown in Figure 4, in the KM analysis, curves depict the relationship between survival time and survival rate. The horizontal axis represents the survival time of cancer patients, measured in months or years. The vertical axis indicates the survival rate, reflecting the proportion of patients surviving at a specific time point. The curve depicts the gradual decline in patient survival over time, starting from diagnosis, with its height reflecting the proportion of surviving patients at each time point(Figure 4a).

The COX analysis(Figure 4b) assesses the correlation between gene expression and patient survival. The significance of this correlation is determined by the *P*-value, where a value greater than 0.05 indicates no significant correlation. The risk level of a gene is represented by the Hazard Ratio (HR) value. HR that greater than 1 indicates a high-risk gene, implying a positive relationship between gene expression and patient risk. Conversely, HR that less than 1 suggests a low-risk gene, indicating an inverse relationship between gene expression and patient risk, for instance, i.e., data points positioned to the right of the dashed line indicate high-risk genes within the tumor, while data points to the left of the dashed line suggest low-risk genes within the tumor.


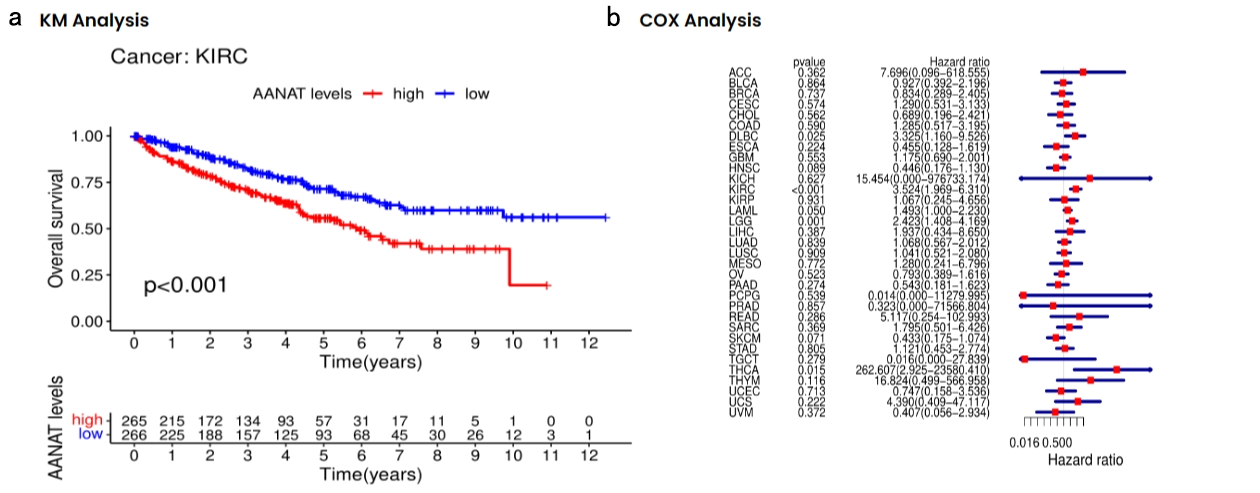


Figure 4. Survival Analysis of single CRG. (a)KM Analysis results. (b)COX Analysis results.

### Clinical Analysis

Under the Single CRG Analysis drop-down, click Clinical Analysis to open and use the analysis. Within the Clinical Analysis section, researchers can access a range of sub-analyses for a thorough investigation:

1. Tumor Mutational Burden. *The number of bases is mutated for every one million bases in each tumor type.*
2. Microsatellite Instability. *Relative to normal cells, microsatellites in tumor cells are unstable due to the insertion or deletion of repetitive units, resulting in an alteration of microsatellite length.*
3. Estimate Correlation. *Scoring is performed for stromal cells and immune cells, where a higher score indicates a greater cellular content of these cells within the tumor cells.*
4. CIBERSORT Correlation. *CIBERSORT utilizes gene expression data to estimate the proportions of different cell types within a mixed cell population.*
5. Clinical Correlation Analysis. *Whether there are differences in the gene expression between different clinical subgroups.*

As shown in Figure 5, the values in the radar plot represent correlation coefficients, where a larger radius of the circular rings corresponds to higher correlation coefficient values. The abbreviations of various tumors are located on the outer side of the concentric rings. The asterisk (*) denotes the significance level.


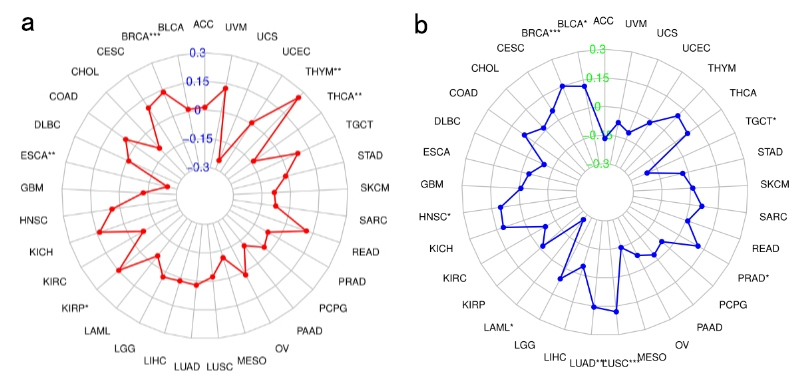


Figure 5. Radar plots in the clinical analysis. (a)Tumor Mutational Burden(TMB) analysis result. (b)Microsatellite Instability(MSI) analysis result.

As shown in Figures 6 and 7, the horizontal axis represents the scoring of cells, corresponding to the yellow-filled band in the upper part. The vertical axis represents gene expression scoring, corresponding to the blue-filled band on the right side. The blue lines within the plot indicate the relationship between gene expression and cell scoring. A positive slope signifies a positive correlation, while a negative slope indicates a negative correlation. The *P*-value reflects the significance of the gene expression and cell relationship. In the Estimate Correlation, cells correspond to the immune and stromal types. In the CIBERSORT Correlation, specific cell types of cancer are considered.


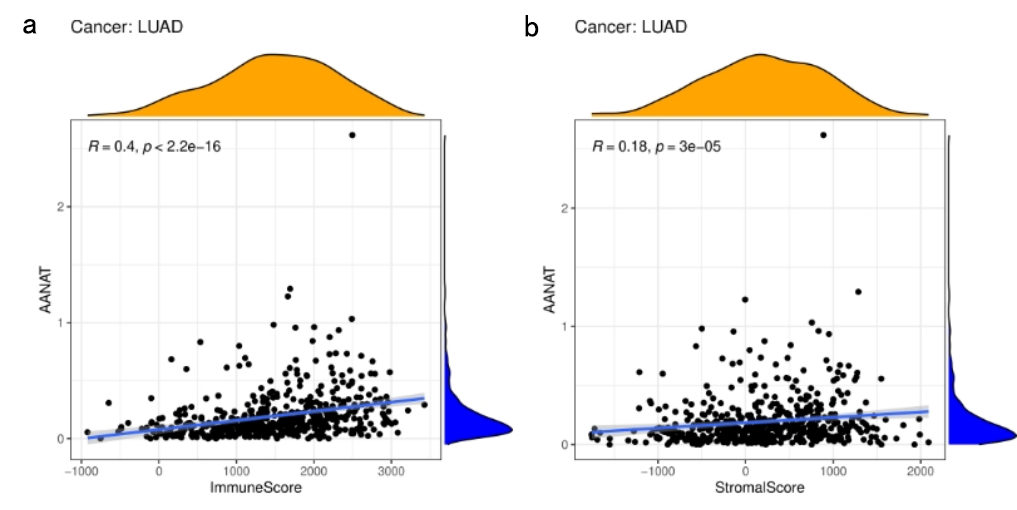


Figure 6. Estimate Correlation in the clinical analysis. (a)The Immune Score of gene AANAT in the Lung adenocarcinoma (LUAD) cancer. (b)The Stromal Score of gene AANAT in the Lung adenocarcinoma (LUAD) cancer.


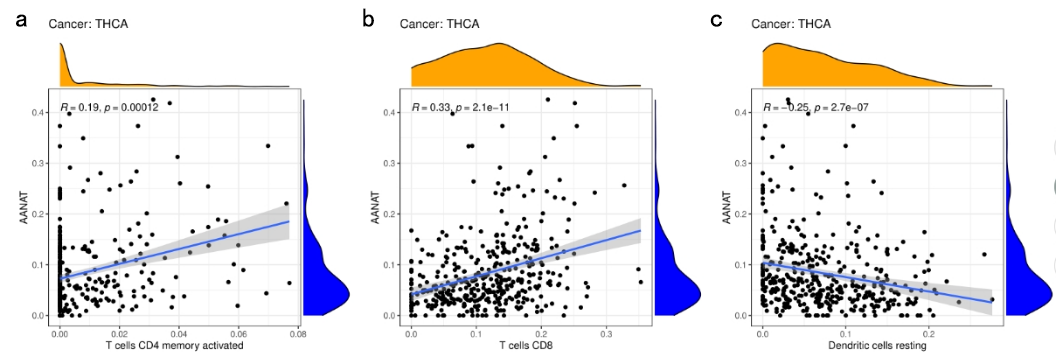


Figure 7. CIBERSORT Correlation in the clinical analysis. (a)The relationship between gene AANAT and T cells CD4 (memory activated) in Thyroid Carcinoma (THCA) cancer. (b)The relationship between gene AANAT and T cells CD8 in Thyroid Carcinoma (THCA) cancer. (c)The relationship between gene AANAT and Dendritic cells resting in Thyroid Carcinoma (THCA) cancer.

As shown in Figure 8, the horizontal axis represents the clinical stage, while the vertical axis represents the expression of the target gene in the given cancer. The endpoints of the black line segments correspond to the two compared stages. The numbers above indicate the *P*-values, indicating the significance of the differences in gene expression among different clinical groups.


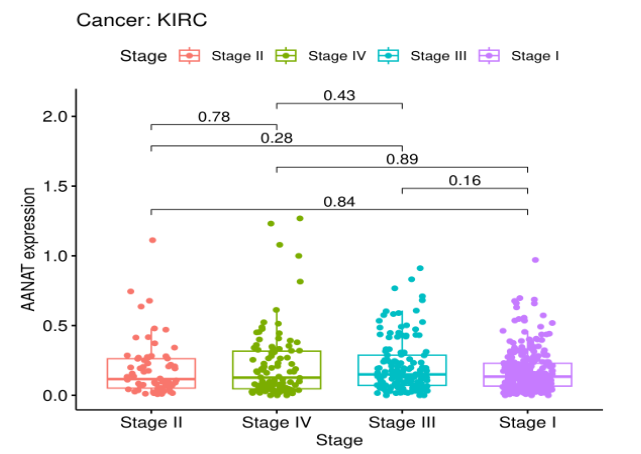


Figure 8. Clinical Correlation Analysis in the clinical analysis.

### Correlation Analysis

Correlation analysis concludes two enrichment analyses, which are the Gene Ontology (GO) enrichment analysis and the Kyoto Encyclopedia of Genes and Genomes (KEGG) enrichment analysis.

1. GO Enrichment Analysis. *Perform GO enrichment analysis on the differentially expressed genes.*
2. KEGG Enrichment Analysis. *Perform KEGG enrichment analysis on the differentially expressed genes.*

Users should input the target gene and choose the target cancer type out of the 33 cancers(Figure 9).


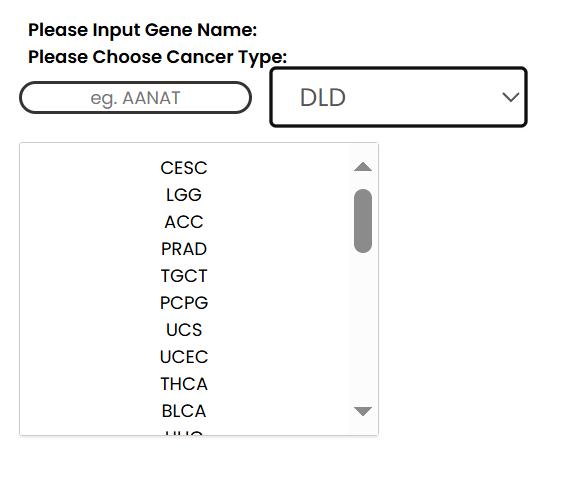


Figure 9. Target gene and target cancer searching bar in the correlation analysis.

Figure 10 shows the presence of the connecting lines between the target gene and GO categories, which indicates the enrichment of the target gene within the corresponding functional categories.


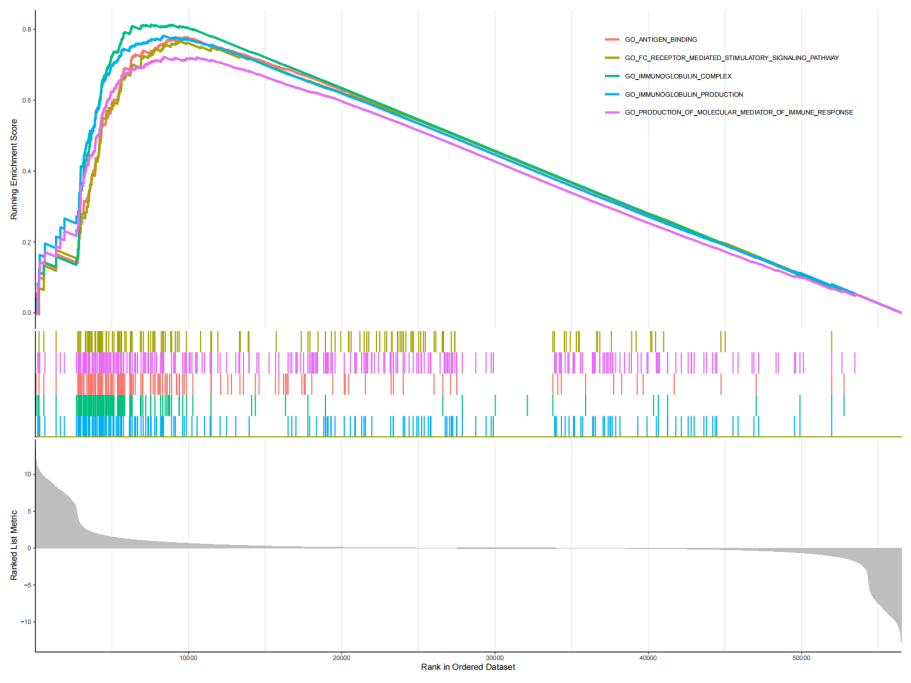


Figure 10. GO Enrichment Analysis in the correlation analysis.

Figure 11 shows the presence of the connecting lines between the target gene and KEGG categories, which indicates the enrichment of the target gene within the corresponding functional categories.


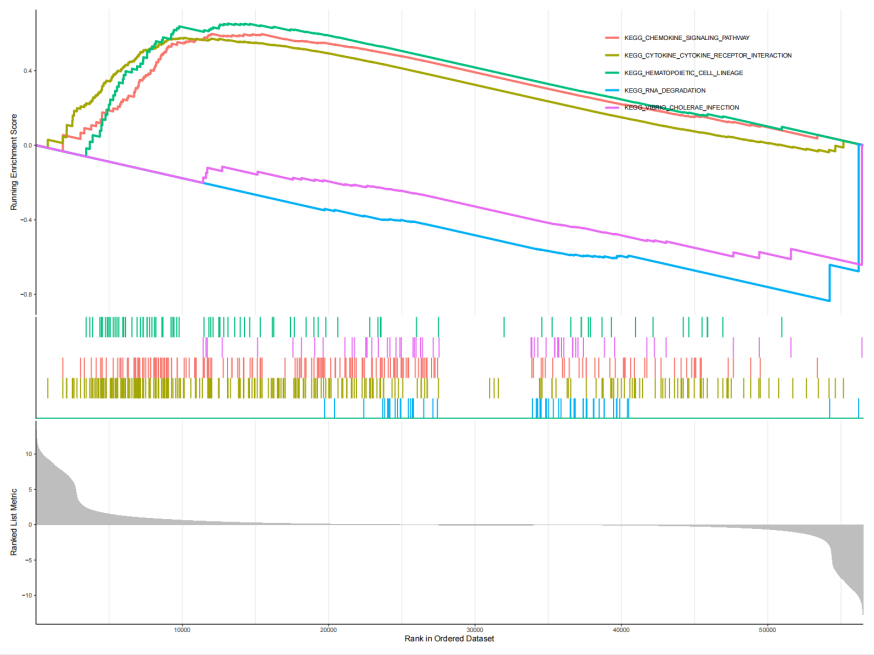


Figure 11. KEGG Enrichment Analysis in the correlation analysis.

### Multi-CRGs Analysis

### Differential Analysis

Under the Multi-CRGs Analysis drop-down, click Differential Analysis to open and use the analysis. Differential Analysis parts are retrieved by the input of the cancer type(Figure 12).

1. Differential Analysis. *Perform the differential expression condition of CRGs through each cancer.*

As shown in Figure 13, the horizontal represents samples, while the vertical axis represents differentially expressed genes. The differential expression condition of CRGs is presented in two plots, which are the heatmap and the volcano plot, respectively. According to the heatmap(Figure 13a), samples are grouped into normal and tumor categories, while the color scale indicates expression levels, with blue representing low expression and red representing high expression in each sample. This allows for the extraction of gene expression levels for differentially expressed genes in each sample. The volcano plot (Figure 13b) straightly shows users the genes that are up-regulated(red) and the genes that are down-regulated(green).


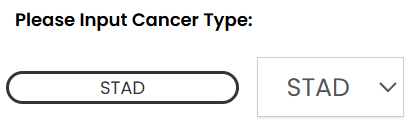


Figure 12. The cancer search bar in the differential analysis.


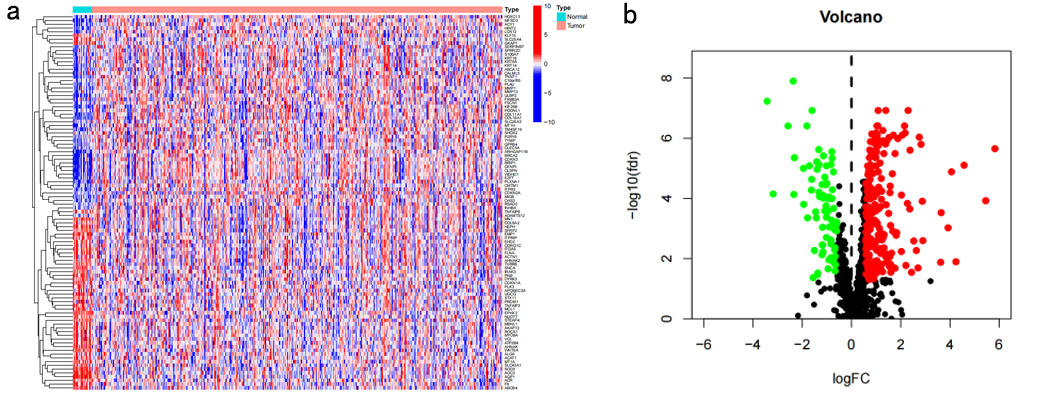


Figure 13. Differential Gene Heatmap and Volcano Plot in the differential analysis. (a) Differential expression heatmap. (b) Volcano plot.

### Model Presentation and Verification

Under the Multi-CRGs Analysis drop-down, click Model Presentation and Verification to open and use the analysis. This part’s retrieval is completed by the input of the target cancer type. The analysis in this part includes:

1. Prognostic Gene Prediction. *The selection of prognostic genes.*
2. Model presentation. *The presentation of the prognostic model and its formula.*
3. Independent Prognostic Analysis. *The verification of the accuracy of the prognostic model.*
4. Receiver Operating Curve. *The verification of the accuracy of the prognostic model.*

CuPCA found the possible gene for the first time that could be put into the construction of the prognostic model by doing survival analysis on all of the CRGs. The CRGs that were mostly related to the risk of patients would be selected as the prognostic genes. The result of Prognostic Gene Prediction is presented with a forest plot (Figure 14). The prognostic genes are listed at the right of the plot, while the left of the plot represents the categories of the genes (high-risk or low-risk) and their degree of impact on each cancer.


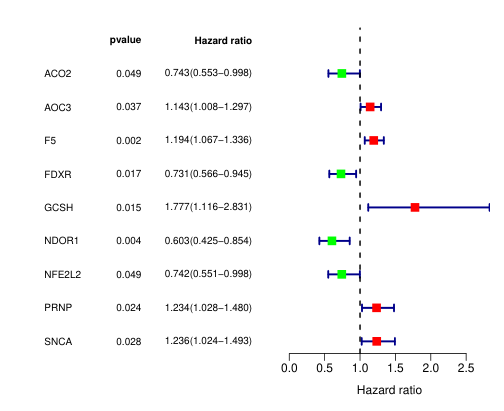


Figure 14. Prognostic Gene Prediction.

By processing and integrating the expression data and survival data from the TCGA/GEO databases, the differential gene expression levels were determined. The TCGA dataset was utilized as the training set to build a prognostic model, while the GEO dataset was employed as the test set to validate the model's accuracy. The model construction process involved deriving a mathematical formula based on the constructed model, which was then used to calculate the risk scores for each sample.

As shown in the example, the formula for Risk score is as follows:

$$Risk score=\sum_{i=0}^{n} \mathrm{Gene}_{i}\times\mathrm{Coef}_{i}$$

The samples were divided into two groups of high and low risk based on the median value of the risk score. The model presentation part includes two Least Absolute Shrinkage and Selection Operator (LASSO) analysis plots(Figure 15), while the model verification part includes 2 parts, which are the Independent Prognostic analysis and the Receiving Operating Curve.


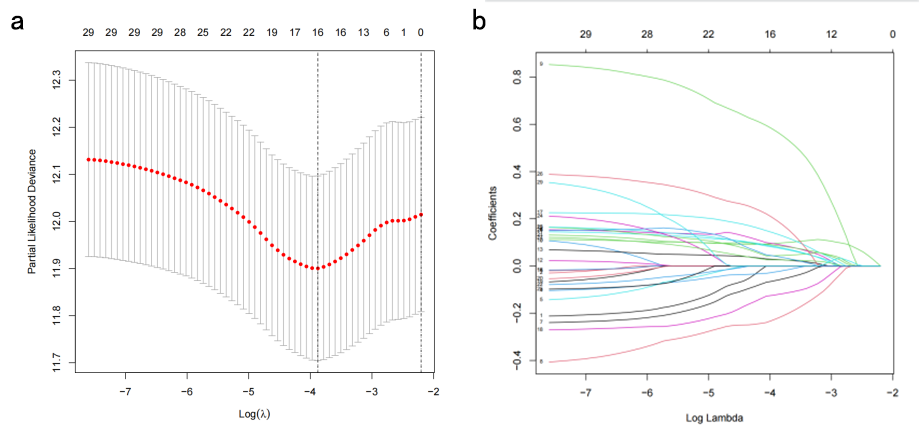


Figure 15. Model Presentation. (a)LASSO cross-validation results.(b)LASSO Regression results.

The Independent Prognostic Analysis determines whether the constructed model can be used as an independent prognostic factor, independent of other clinical features(Figure 16). The Receiving Operating Curve evaluates the model's performance in predicting patient survival in comparison to other clinical features, thereby assessing its superiority(Figure 17).


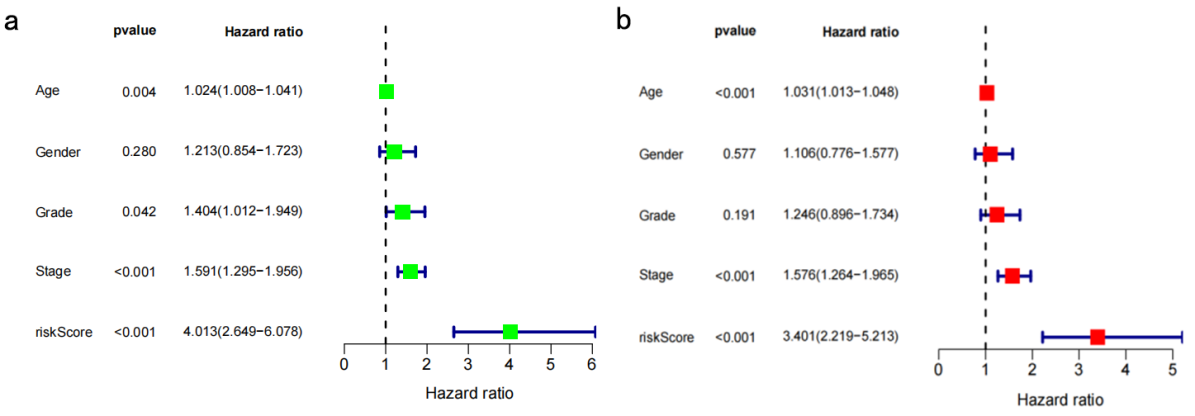


Figure 16. Independent prognostic analysis. (a) Uni-variate independent prognostic analysis. (b)Multi-variate independent prognostic analysis.


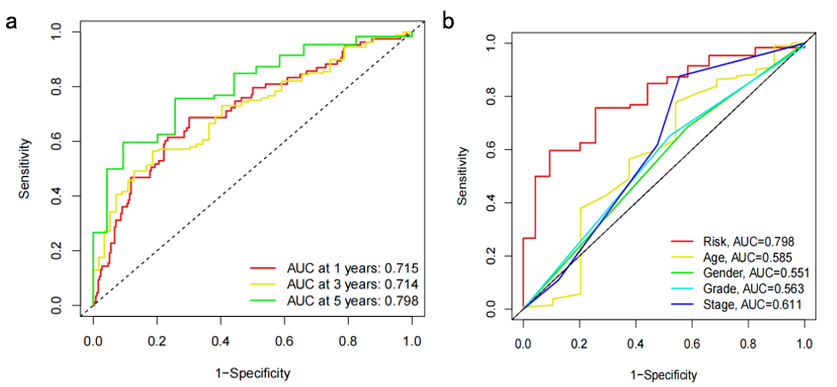


Figure 17. Receiving Operating Curve. (a)ROC results based on clinical factors. (b)ROC results based on survival years.

### Correlation Analysis

Under the Multi CRGs Analysis drop-down, click Correlation Analysis to open and use the analysis. Correlation Analysis results are retrieved by the input of cancer type. Within the Correlation Analysis section, researchers can access a range of sub-analyses for a thorough investigation and the relationship between gene and gene, gene and immune functions, gene and pathways, and gene and mutation categories:

1. Gene Set Variation Analysis. *Observe the functional or pathway activities that are active in the high-risk group and those that are active in the low-risk group.*
2. GO Enrichment Analysis. *Perform the GO enrichment analysis on the differentially expressed genes.*
3. KEGG Enrichment Analysis. *Perform the KEGG enrichment analysis on the differentially expressed genes.*
4. Protein-Protein Interaction. *Display the protein-protein interactions.*
5. Cytoscape Visualization. *Visualize the protein-protein interaction network.*

As shown in Figure 18, the horizontal axis represents the samples, which are categorized based on their risk levels. Samples belonging to the low-risk group are denoted in blue, while those in the high-risk group are indicated in red. The vertical axis represents the pathway name, and the color within each pathway indicates the level of gene expression. The color red signifies high expression, while blue represents low expression.


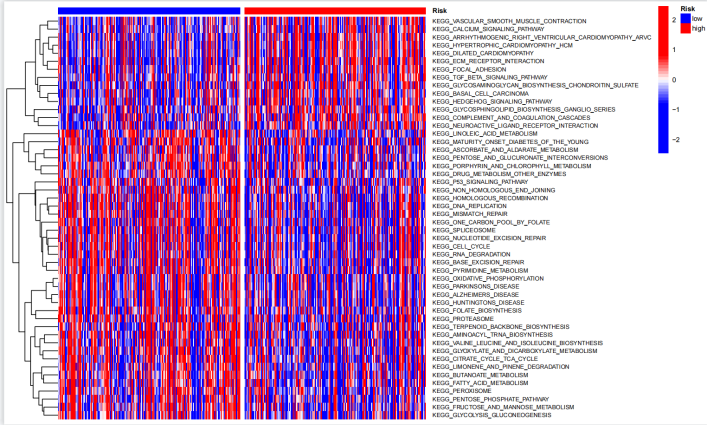


Figure 18. Gene Set Variation Analysis in the correlation analysis.

GO Enrichment Analysis indicates the enrichment condition of CRGs in certain pathways or certain immune cells in each cancer. GO enrichment analysis result presents 3 plots of different types in total, which include a bar plot(Figure 19a), a bubble plot(Figure 19b), and a circle plot(Figure 19c).


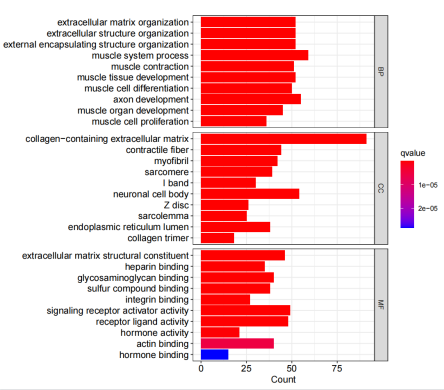

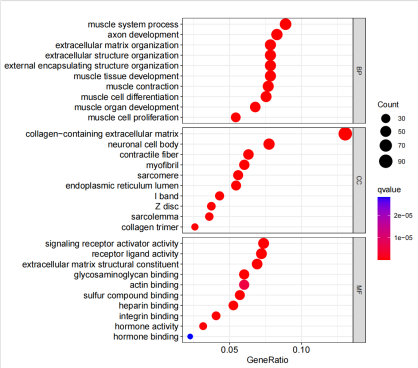

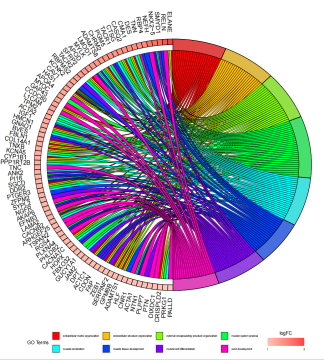


Figure 19. GO Enrichment Analysis in the correlation analysis. (a)Bar plot version of GO results. (b)Bubble plot version of GO results. (c)Circle plot version of GO results.

KEGG Enrichment Analysis indicates the enrichment condition of CRGs in certain pathways or certain immune cells in each cancer. KEGG enrichment analysis result presents 3 plots of different types in total, which include a bar plot(Figure 20a), a bubble plot(Figure 20b), and a circle plot(Figure 20c).


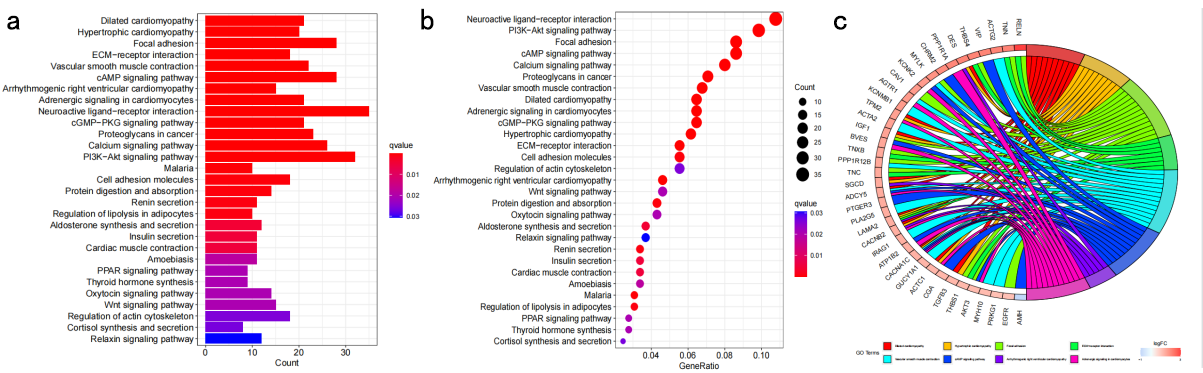


Figure 20. KEGG Enrichment Analysis in the correlation analysis. (a)Bar plot version of KEGG results. (b)Bubble plot version of KEGG results. (c)Circle plot version of KEGG results.

As shown in Figure 21, nodes represent genes or proteins, and edges indicate the interactions between them. When there is a connection between two nodes, it signifies the presence of PPI between the corresponding genes or proteins. The color of the edges reflects the degree of evidence supporting the PPI results.


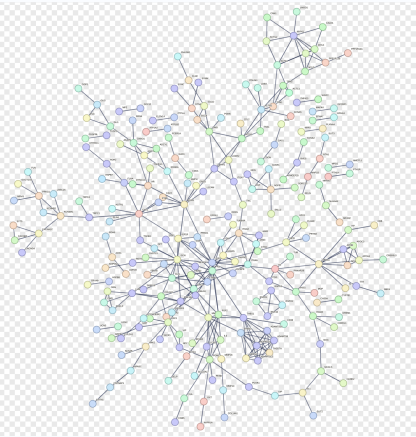


Figure 21. Protein-Protein Interaction Network in the correlation analysis.

As shown in Figure 22, nodes correspond to genes or proteins, and an edge between two nodes signifies the presence of PPI between the associated genes or proteins. The color of the nodes indicates their expression level, with upregulated genes or proteins represented in red and downregulated ones in green.


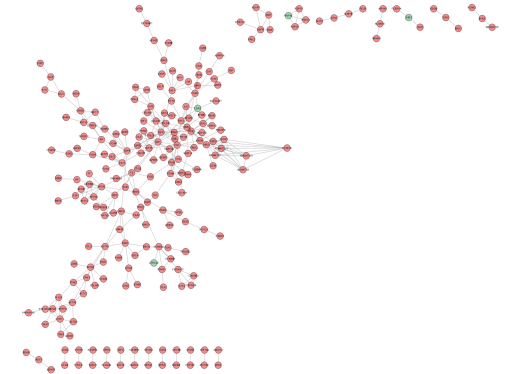


Figure 22. Cytoscape Visualization in the correlation analysis.

### Mutation Analysis

Under the Multi-CRGs Analysis drop-down, click Mutation Analysis to open and use the analysis. Mutation Analysis results are retrieved by the input of cancer type. Within the Mutation Analysis section, researchers can access the mutation condition of prognostic CRGs in each cancer.

1. Prognostic Gene Waterfall Plot. *Mutation frequency of prognostic genes in each cancer.*

As shown in Figure 23, the horizontal axis represents the samples, while the vertical axis represents the status of genes associated with the prognosis. Different mutation types are depicted using distinct colors, enabling the acquisition of mutation frequency information for each gene. The waterfall plot (Figure 23a) is always provided with a co-mutation plot(Figure 23b), presenting the possibility of co-mutation among the prognostic genes in the waterfall plot.


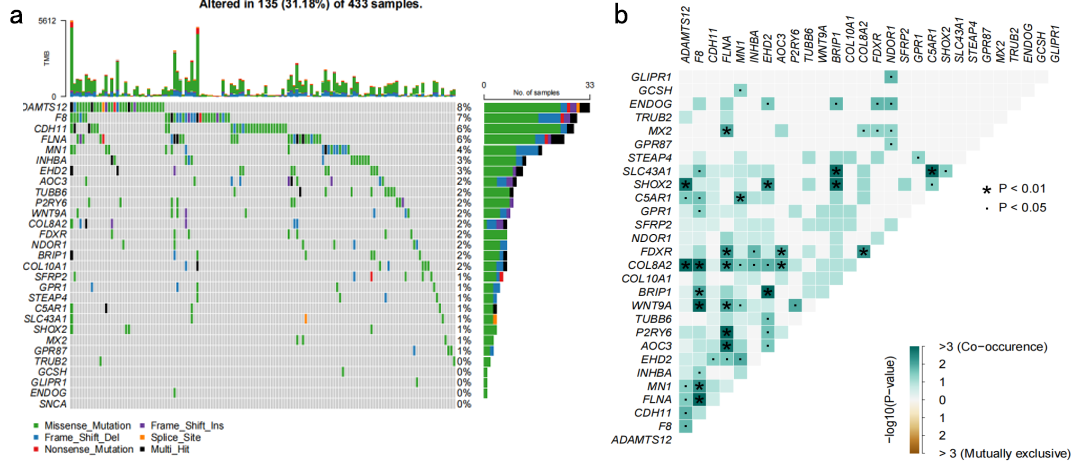


Figure 23. Prognostic Gene Waterfall plot. (a)Waterfall plot. (b) Co-mutation plot.

### Survival Analysis

Under the Multi-CRGs Analysis drop-down, click Survival Analysis to open and use the analysis. Within the Survival Analysis section, researchers can access a range of sub-analyses for a thorough investigation:

1. Overall Survival Analysis. *The prediction of patients’ survival condition in the OS period.*
2. Progression-Free Survival Analysis. *The prediction of patients’ survival condition in the PFS period.*

OS (Figure 24a) and PFS analysis (Figure 24b) can show the survival prediction of patients in the OS and PFS period.


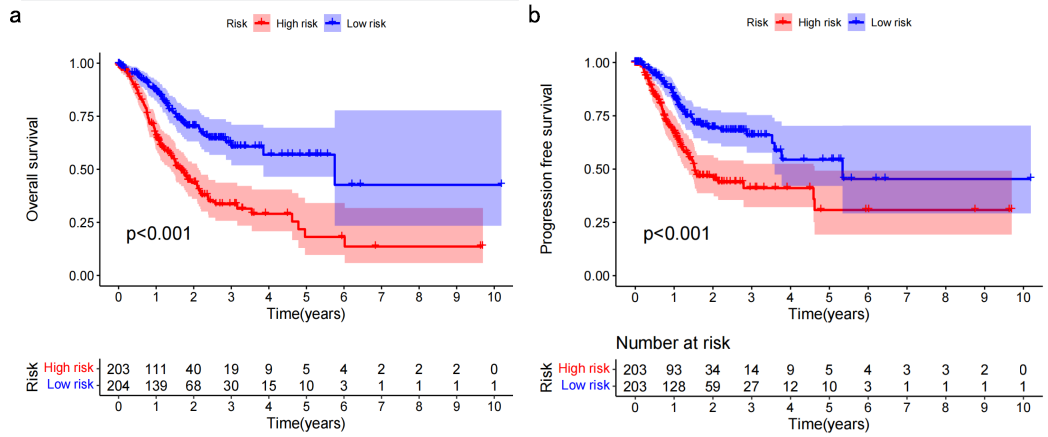


Figure 24. Overall Survival (OS) Analysis results and Progression-Free Survival (PFS) Analysis results. (a)Overall Survival Analysis result. (b)Progression-Free Survival Analysis result.

### Clinical Analysis

Under the Multi-CRGs Analysis drop-down, click Clinical Analysis to open and use the analysis. Within the Clinical Analysis section, researchers can access a range of sub-analyses for a thorough investigation:

1. Clinical Correlation Analysis. *Figure out the differences in the risk scores of patients among various clinical features.*
2. Nomogram Presentation. *Predict the patient's survival time and condition in the future according to their risk score.*
3. Nomogram Independent Prognostic Analysis. *Predict the accuracy of the nomogram.*
4. Nomogram Receiver Operating Curve. *Predict the accuracy of the nomogram.*

Clinical correlation analysis presents the difference in the risk scores of patients among various clinical features, which include age, gender, grade, and stage. For instance, Figure 25 shows the clinical correlation analysis results of patients of different ages.


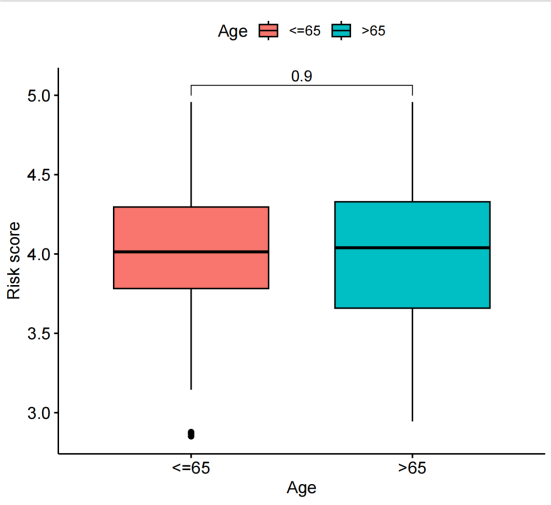


Figure 25. Clinical Correlation Analysis in the clinical analysis.

The Nomogram (Figure 26a) is equipped with individual scales, allowing for independent scoring of each clinical feature. The scores of these clinical features are then aggregated to obtain a composite score, which is used to predict patient survival based on the corresponding scale (Figure 26b).


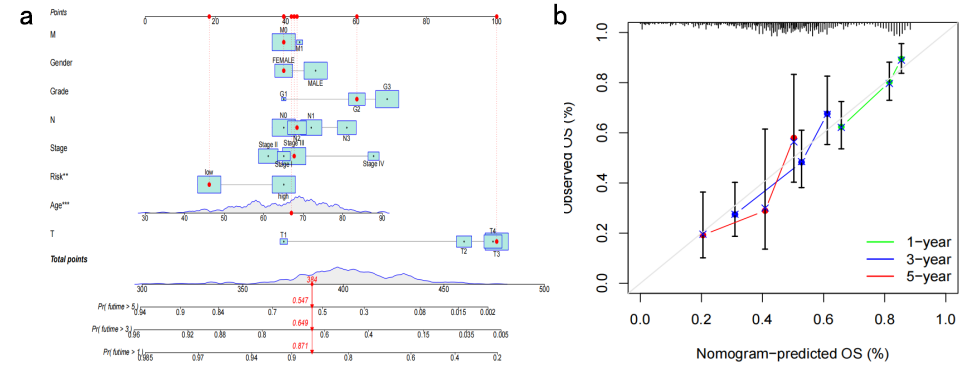


Figure 26. The Nomogram and its calibration plot in the clinical analysis. (a) Nomograom. (b) Calibration plot.

The Nomogram is always presented with a calibration plot and two verification plots to show its correctness, which are the Nomogram Independent Prognostic Analysis plot (Figure 27a) and the Nomogram Receiver Operating Curve (Figure 27b). The Independent Prognostic Analysis plot indicates whether the nomogram can exist as an independent prognostic factor independent of other clinical shapes. The ROC curve shows the ability of the nomogram to calculate the risk score of each clinical factor


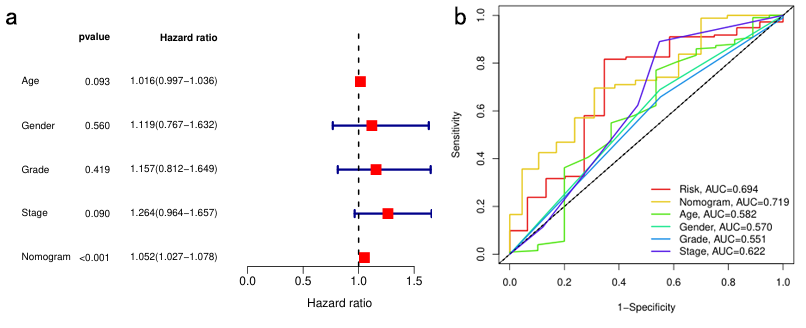


Figure 27. The Nomogram verification plots in the clinical analysis. (a) Indenpendent Prognostic Analysis plot. (b) Receiver Operating Curve.

### Immune Analysis

Under the Multi-CRGs Analysis drop-down, click Immune Analysis to open and use the analysis. Within the Immune Analysis section, researchers can access a range of sub-analyses for a thorough investigation:

1. Immunotyping Analysis. *Examine whether there exist disparities in the risk scores of patients across various immune subtypes.*
2. Immune Cell Differential Analysis. *Observe the associations between the immune cells and risk-differentiated groups.*
3. Immune-related Functions Analysis. *Discover the difference in various immune-related functions between the high-risk and the low-risk groups.*
4. Immunotherapy Analysis. *Identify the subgroup within the high-risk and low-risk groups that demonstrate better response to immune therapy.*

As shown in Figure 28, the results of the Immunotyping Analysis can indicate significant differences in patient risk scores across different immune subtypes. In this box plot, the horizontal axis represents different immune subtypes, while the vertical axis represents the patients’ risk scores. A *P-*value is shown between each of the two immune groups to quantify the significance of the difference. In this example, we can figure out that patients from the C3 group are at a higher risk compared with other groups. There is no significant risk difference between all groups of patients except for patients in groups C2 and C3.


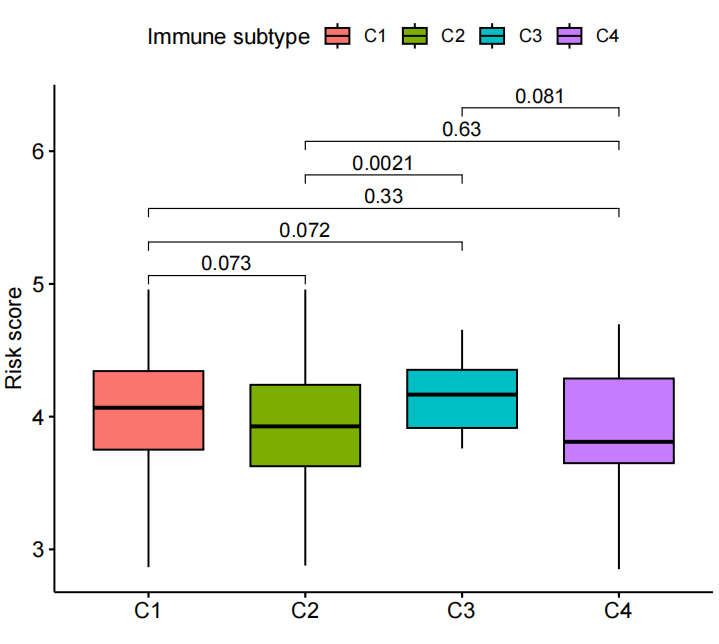


Figure 28. Immunotyping Analysis in the immune analysis.

As shown in Figure 29, the horizontal axis represents the names of immune cells, while the vertical axis represents the quantities of immune cells.


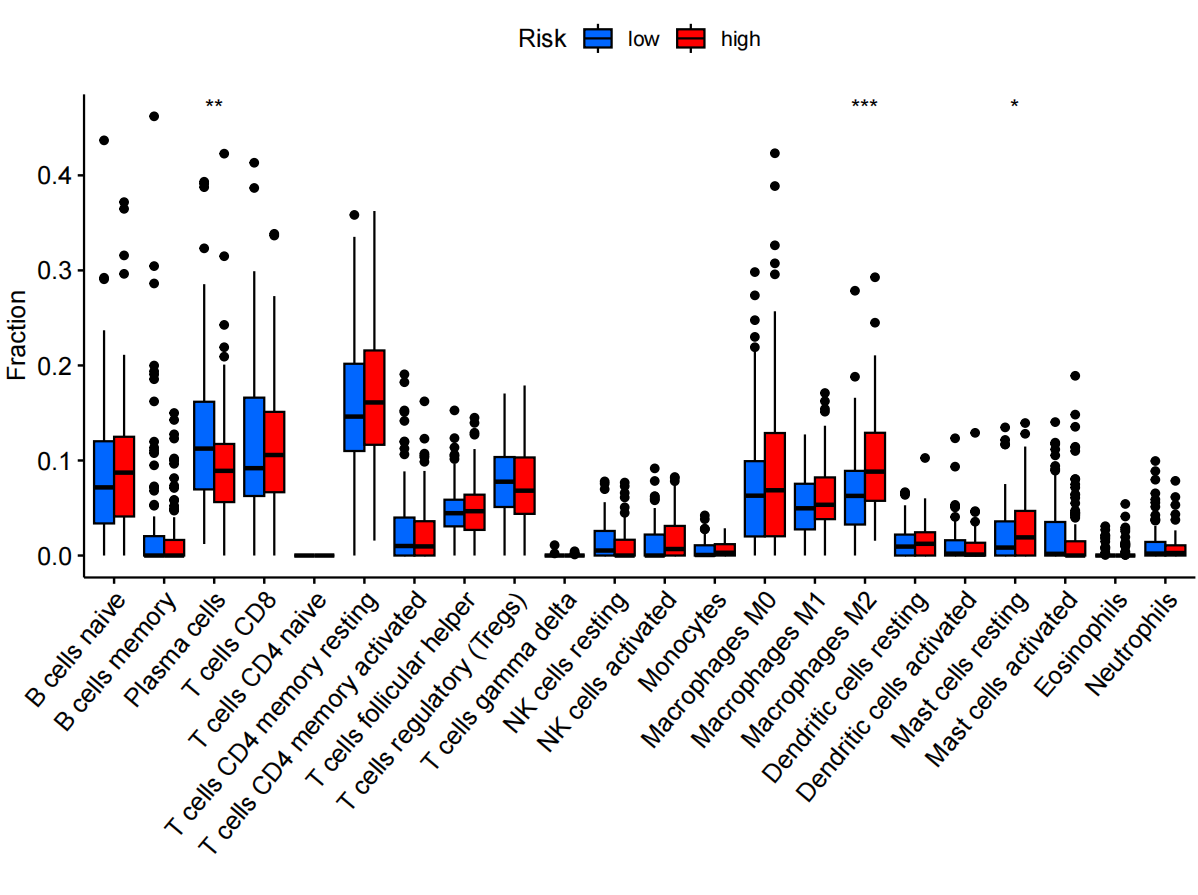


Figure 29. Immune Cell Differential Analysis in the immune analysis.

As shown in Figure 30, the horizontal axis represents immune-related functions, while the vertical axis represents the scores of immune-related functions. Higher scores indicate more active immune-related functions in the sample. The presence of asterisks above the region of immune activity indicates differences in immune cell activity between the high-risk and low-risk groups.


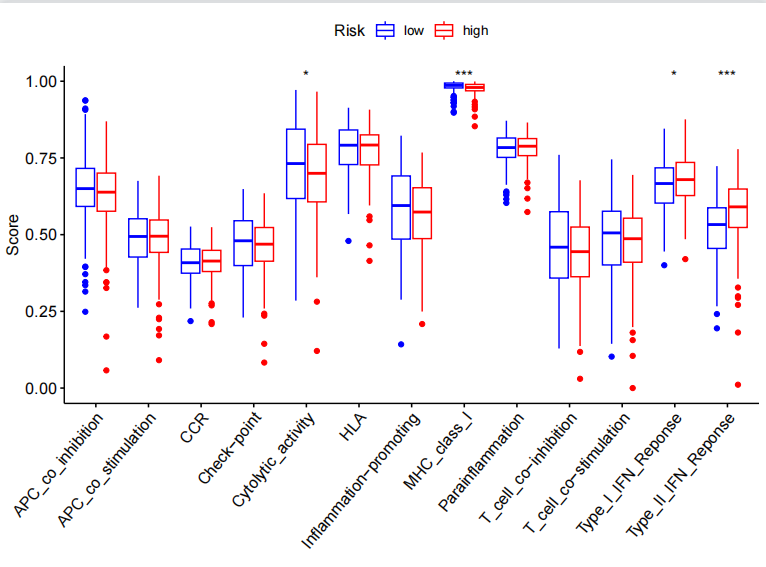


Figure 30. Immune-related Functions in the immune analysis.

As shown in Figure 31, the horizontal axis represents the patients' risk, while the ordinate represents the Institute for Democracy and Electoral Assistance (TIDE) score. A higher score indicates a greater potential for immune evasion, which implies a poorer response to immune therapy in patients.


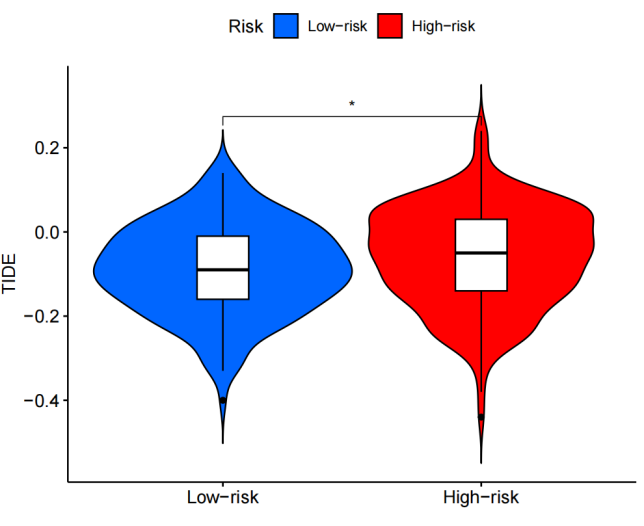


Figure 31. Immunotherapy Analysis in the immune analysis.

### Dimensionality Reduction

Under the Multi-CRGs Analysis drop-down, click Dimensionality Reduction to open and use the analysis. This part includes one analysis, which is:

1. Principle Component Analysis. *Present the ability of the prognostic genes to figure out the high-risk and the low-risk patients.*

The result (Figure 32) reveals whether the prognostic model can distinguish the high-risk patients and low-risk patients.


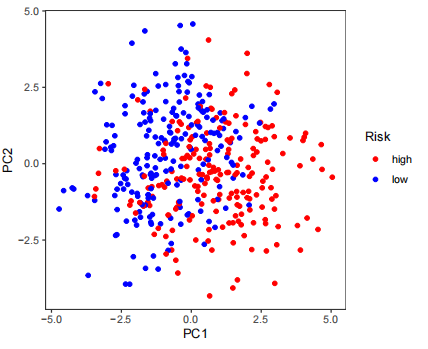


Figure 32. Principle Component Analysis (PCA) in the dimensionality reduction.

### CRlncRNA Analysis

### Model Presentation and Verification

Under the CRGlncRNA Analysis drop-down, click Model Presentation and Verification to open and use the analysis. A prognostic model was constructed on these CRlncRNA. The samples were divided into a Training set and a Test set. The prognostic model was built using the Training set to derive the model formula. The risk scores were calculated for the Test set. The formula for the Risk score is as follows:

$$Risk score=\sum_{i=0}^{n} \mathrm{lncRNA}_{i}\times\mathrm{Coef}_{i}$$

The analysis of this part includes:

1. Model presentation. *The presentation of the prognostic model and its formula.*
2. C-index Verification. *The verification of the accuracy of the prognostic model.*
3. Independent Prognostic Analysis. *The verification of the accuracy of the prognostic model.*
4. Receiver Operating Curve. *The verification of the accuracy of the prognostic model.*

The Test set patients were then classified into the high-risk and low-risk groups based on the median risk score of the Training set. In the model presentation part, the formula mentioned above is presented with the LASSO analysis result (Figure 33a and Figure 33b) and the uniforest plot(Figure 33c).


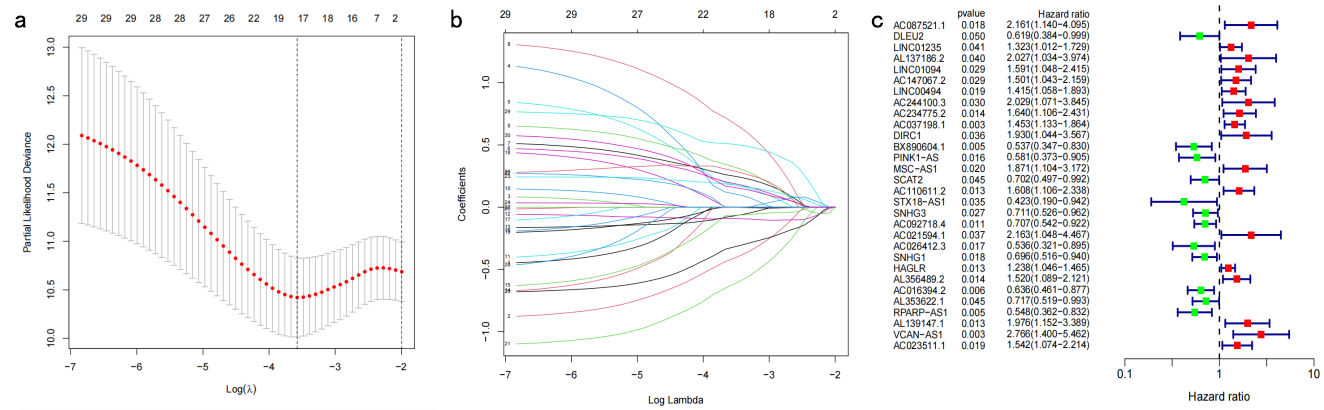


Figure 33. Model Presentation in the CRlncRNA analysis. (a)LASSO cross-validation results. (b)LASSO Regression results. (c)Uniforest plot.

The model verification part provides three types of analysis, which are C-index verification (Figure 34a), Independent verification (Figure 34b), and ROC (Figure 34c).

*
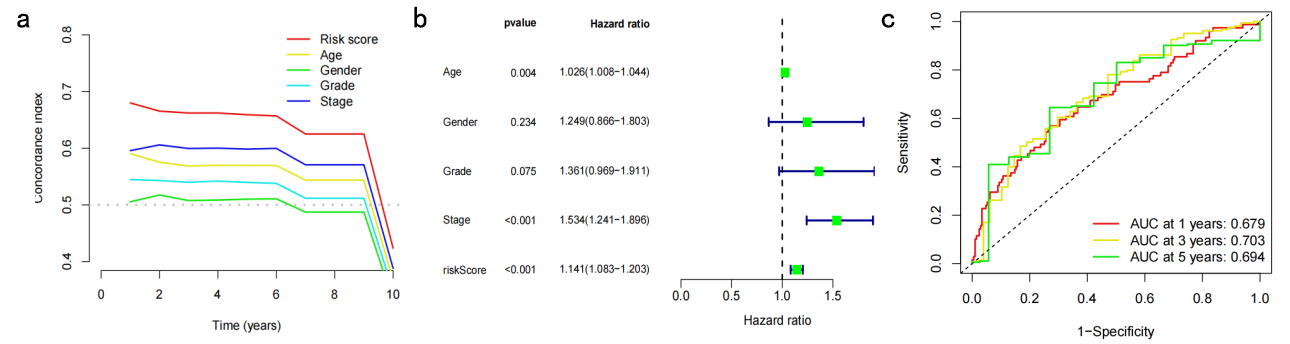
*

Figure 34. Model Verification in the CRlncRNA analysis. (a)C-index verification plot. (b)Independent verification plot. (c)ROC.

### Correlation Analysis

Under the CRGlncRNA Analysis drop-down, click Correlation Analysis to open and use the analysis. Within the Correlation Analysis section, researchers can access a range of sub-analyses for a thorough investigation.

1. Co-expression Analysis. *Find out the CRlncRNAs and their relationship between CRGs, presented in the form of a chart.*
2. Correlation Heatmap. *The visualization of the co-expression chart, indicating the relationship between CRlncRNAs and CRGs.*
3. GO Enrichment Analysis. *Perform GO enrichment analysis on the differentially expressed genes.*
4. KEGG Enrichment Analysis. *Perform KEGG enrichment analysis of differentially expressed genes.*

As shown in Figure 35, the table displays the association between Cuprotosis, which represents genes related to copper-induced cell death, and lncRNA, which denotes lncRNAs that exhibit co-expression with the copper-related genes. The core column represents the correlation coefficient, where values greater than 0 indicate a positive regulatory relationship, and values less than 0 indicate a negative regulatory relationship. A *P*-value of less than 0.05 is considered significant for co-expression, and the regulation column indicates whether the relationship is positive or negative. Users should input the cancer type, and target gene to gain the related lncRNA and the related details(Figure 35).


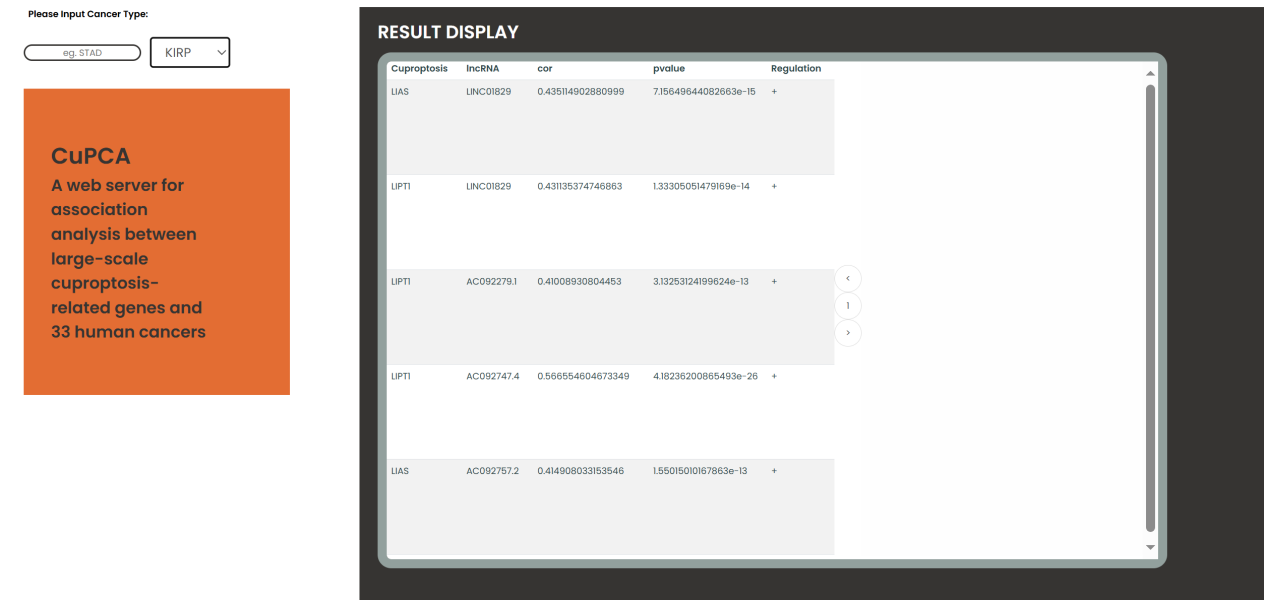
Figure 35. Co-expression Analysis table in the correlation analysis.

As shown in Figure 36, the horizontal axis represents genes associated with mortality, and the vertical axis represents lncRNAs used for model construction. A heatmap illustrating the correlation between them is generated, with blue indicating a negative correlation and red indicating a positive correlation. The presence of an asterisk (*) in a cell signifies a significant correlation. A significant positive correlation indicates a positive regulatory relationship, while a significant negative correlation indicates a negative regulatory relationship.


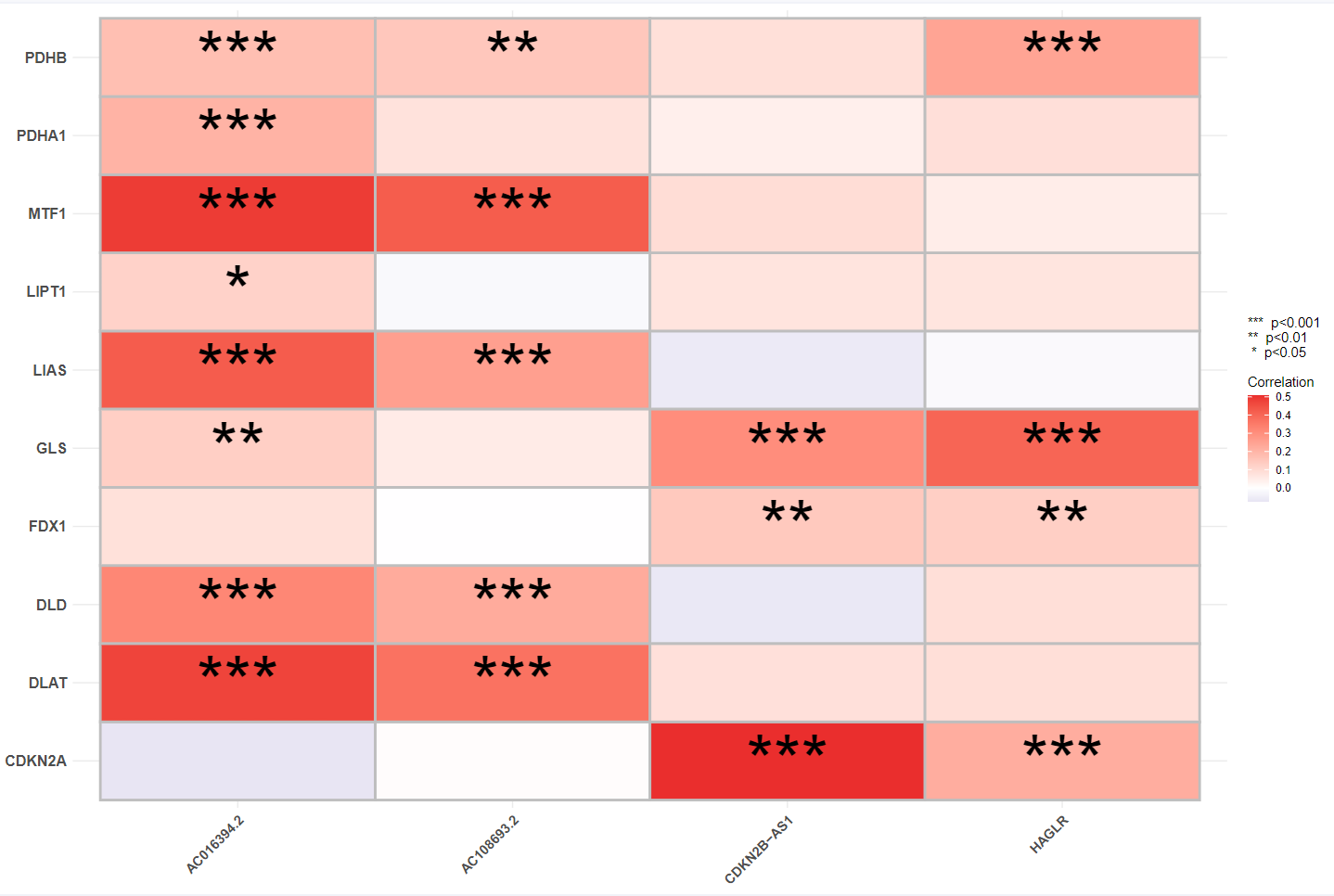


Figure 36. Correlation Heatmap in the correlation analysis*.*

As shown in Figure 37, the horizontal axis represents the names of GO terms (Figure 37a), while the vertical axis represents the number of occurrences of each GO term. Different colors indicate the classification of GO terms. The GO enrichment analysis also has its bubble version (Figure 37b) and circle version (Figure 37c).


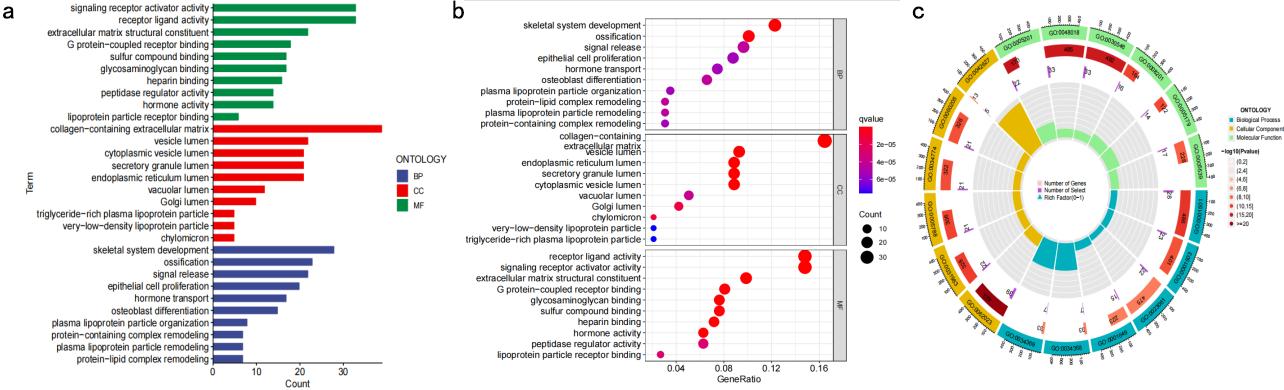


Figure 37. GO Enrichment Analysis in the correlation analysis. (a)Bar plot version of GO results. (b)Bubble plot version of GO results. (c)Circle plot version of GO results.

KEGG Enrichment Analysis indicates the enrichment condition of CRlncRNAs in certain pathways or certain immune cells in each cancer. KEGG enrichment analysis result presents 3 plots of different types in total, which include a bar plot(Figure 38a), a bubble plot(Figure 38b), and a circle plot(Figure 38c).


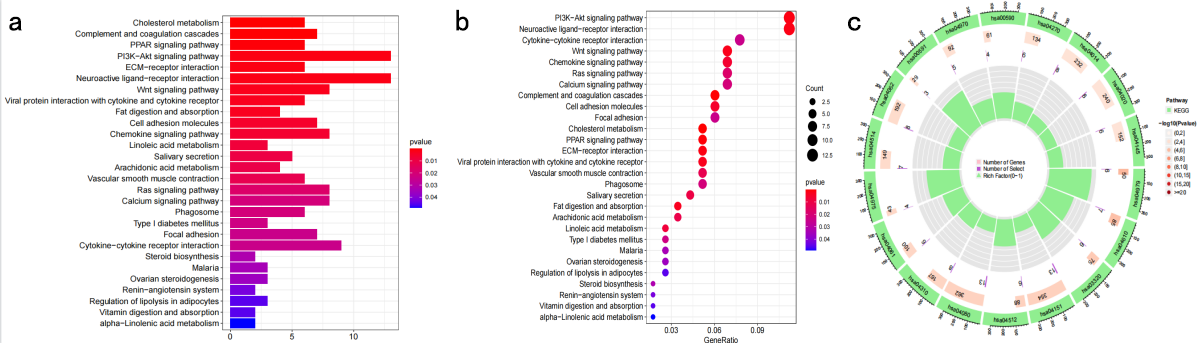


Figure 38. KEGG Enrichment Analysis in the correlation analysis. (a)Bar plot version of KEGG results. (b)Bubble plot version of KEGG results. (c)Circle plot version of KEGG results.

### Mutation Analysis

Under the CRGlncRNA Analysis drop-down, click Mutation Analysis to open and use the analysis. Mutation analysis predicts the mutation condition of different tumors in the high-risk patients group and the low-risk patients group.

1. Tumor Mutation Load Score. *Calculate the TMB score of each sample and compare the mutation frequency of patients between the high-risk group and the low-risk group.*
2. Tumor Mutation Differential Analysis. *Investigate the potential disparity in tumor mutational burden between the high-risk and low-risk groups.*
3. Tumor Mutation Burden Survival Analysis. *Investigate the potential disparity in patient survival between the high tumor mutational burden group and the low tumor mutational burden group.*

As shown in Figure 39, the horizontal axis represents sample names, and the vertical axis represents gene names. Different colors are used to indicate distinct types of mutations. The graphical representation illustrates the mutation frequency of each gene, with the gene names and order being identical in both graphs.


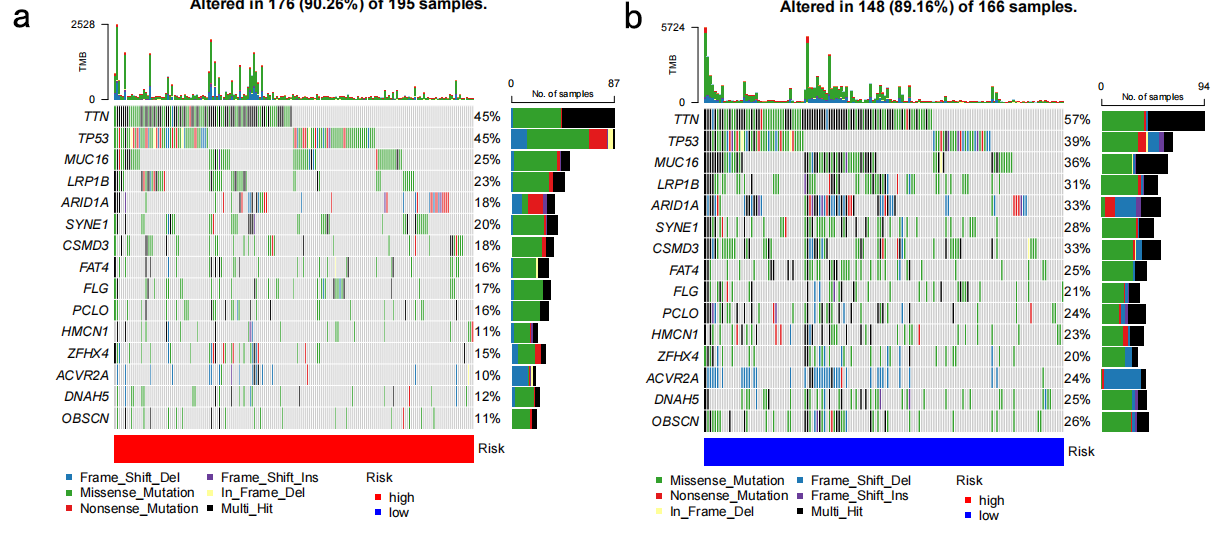


Figure 39. Tumor Mutation Load Score in the mutation analysis. (a)Waterfall Plot of high-risk patients. (b)Waterfall plot of low-risk patients.

As shown in Figure 40, the horizontal axis represents the survival time (in years), while the vertical axis represents the survival rate. Based on the tumor mutational burden of patients, they are divided into high and low mutational burden groups. The aim is to compare the differences in patient survival between these two groups and obtain the *P*-value for the observed differences. If the *P*-value is less than 0.05, it indicates that there is a significant difference in patient survival between the high and low mutational burden groups.


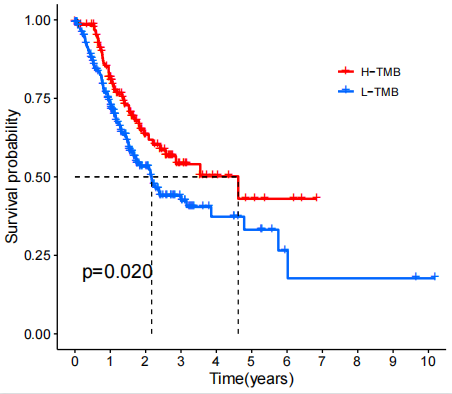


Figure 40. Tumor Mutation Burden Survival Analysis in the clinical analysis*.*

As shown in Figure 41, the horizontal axis represents the risk levels of patients, with the high-risk group indicated in red color and the low-risk group indicated in blue color, while the vertical axis represents the tumor mutation burden of patients. A *P*-value less than 0.05 indicates a significant difference in tumor mutation burden between these two groups.


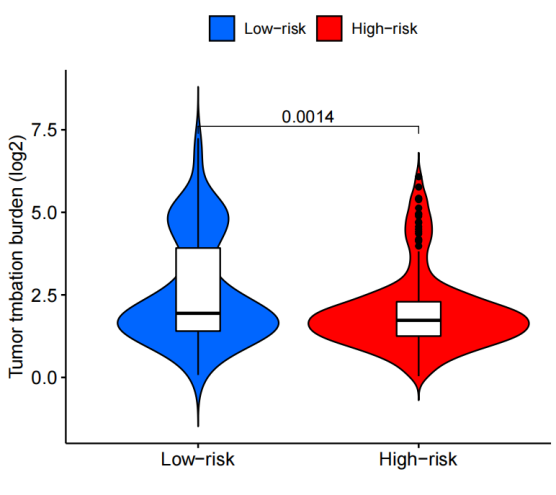


Figure 41. Tumor Mutation Differential Analysis in the clinical analysis.

### Survival Analysis

Under the CRGlncRNA Analysis drop-down, click Survival Analysis to open and use the analysis. Within the Survival Analysis section, researchers can access a range of sub-analyses for a thorough investigation:

1. Overall Survival Analysis. *The prediction of patients’ survival condition in the OS period.*
2. Progression-Free Survival Analysis. *The prediction of patients’ survival condition in the PFS period*
3. RiskPlot. *The prediction of the influence CRlncRNAs have on patients’ survival condition.*

OS analysis and PFS analysis can be referred to as described earlier concerning these two analyses(Figure 42).


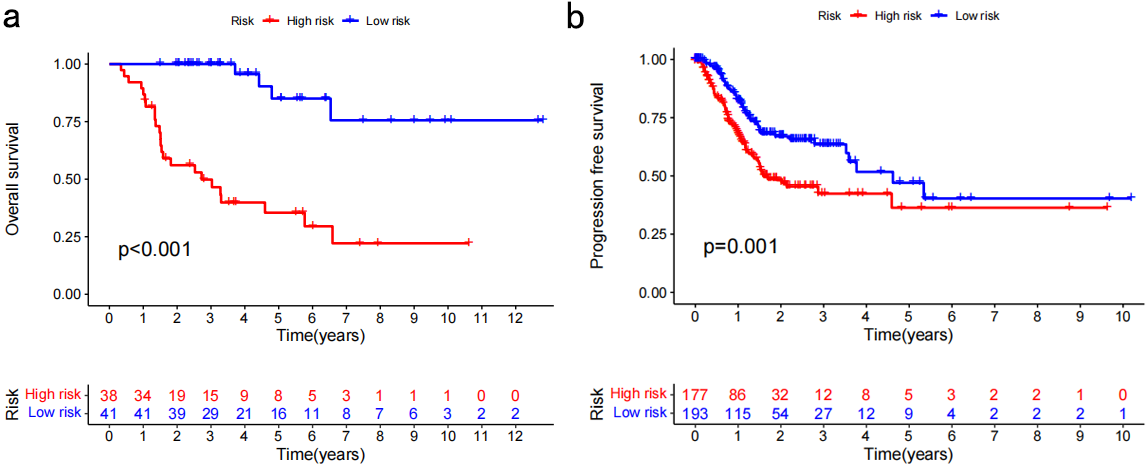


Figure 42. Overall Survival (OS) analysis and Progression-Free Survival (PFS) analysis results in the survival analysis. (a)Overall Survival analysis. (b) Progression-Free Survival Analysis.

As shown in Figure 43, the risk curve consists of three subplots with a consistent abscissa representing the sorted patients in increasing order of risk. Figure 42a shows the risk score of patients on the ordinate. Based on the median risk score, patients are divided into the high-risk group (depicted in red) and the low-risk group (depicted in blue), Figure 42b indicates the survival time (in years) on the ordinate. Red dots represent patients who have died, while blue dots represent patients who are still alive. As patient risk increases, the number of deaths also increases, aligning with expectations. While Figure 42c is a risk heat map, illustrating the high-risk and low-risk lncRNAs. The ordinate represents the expression level of lncRNAs. With increasing patient risk, if the expression of a corresponding lncRNA also increases, it indicates that it is a high-risk lncRNA.


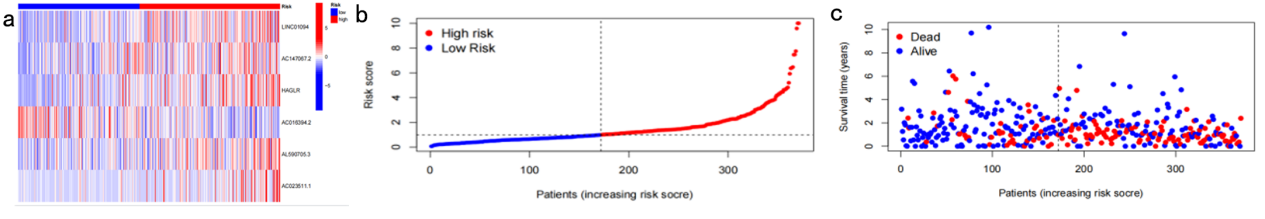


Figure 43. RiskPlot in the survival analysis. (a)Survival heatmap. (b)Survival curve. (c)Survival sample dots.

### Clinical Analysis

Under the CRGlncRNA Analysis drop-down, click Clinical Analysis to open and use the analysis. Within the Clinical Analysis section, researchers can access a range of sub-analyses for a thorough investigation:

1. Model Validation. *Validate the applicability of the constructed model to the different clinical patient subgroups.*
2. Nomogram. *Predict the patient's survival time and condition in the future according to their risk score.*

To assess the applicability of the constructed model to different patient groups, the patients were categorized into early-stage and late-stage groups based on their staging(Figure 44). The model was then applied to both groups separately. If the *P*-values of both models are less than 0.05, it indicates that the constructed model is not only applicable to early-stage patients but also to late-stage patients.


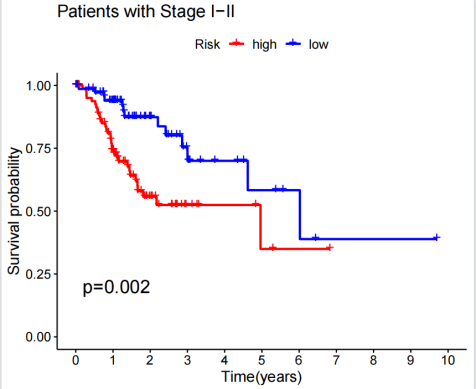


Figure 44. Model Validation in the clinical analysis.

The Nomogram (Figure 45a) is equipped with individual scales, allowing for independent scoring of each clinical feature. The scores of these clinical features are then aggregated to obtain a composite score, which is used to predict patient survival based on the corresponding scale (Figure 45b).


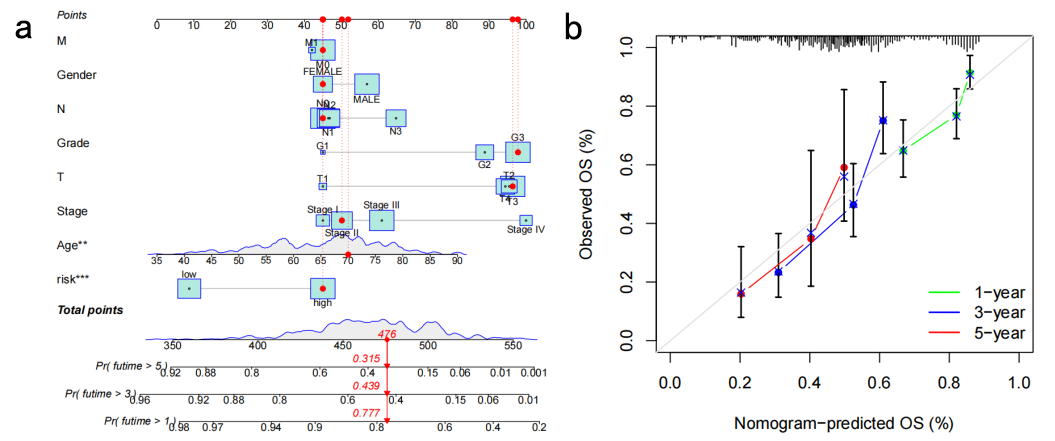


Figure 45. Nomogram in the clinical analysis. (a)Nomogram presentation. (b) Nomogram’s calibration plot.

### Immune Analysis

Under the CRGlncRNA Analysis drop-down, click Immune Analysis to open and use the analysis. Within the Immune Analysis section, researchers can access a range of sub-analyses for a thorough investigation:

1. Immune-related Functions Analysis. *Discover the difference in various immune-related functions between the high-risk and the low-risk groups.*
2. Immune Evasion and Immunotherapy Analysis. *Identify the subgroup within the high-risk and low-risk groups that demonstrate better response to immune therapy.*

Immune-related Functions (Figure 46) can show the type of immune-related functions that differ between high and low-risk groups. The horizontal axis indicates the samples, while green means low-risk samples and red means high-risk samples), and the vertical axis indicates the type of immune-related functions. The blue squares inside the heatmap indicate low CRlncRNA expression samples and the red squares inside the heatmap indicate high CRlncRNA expression samples.


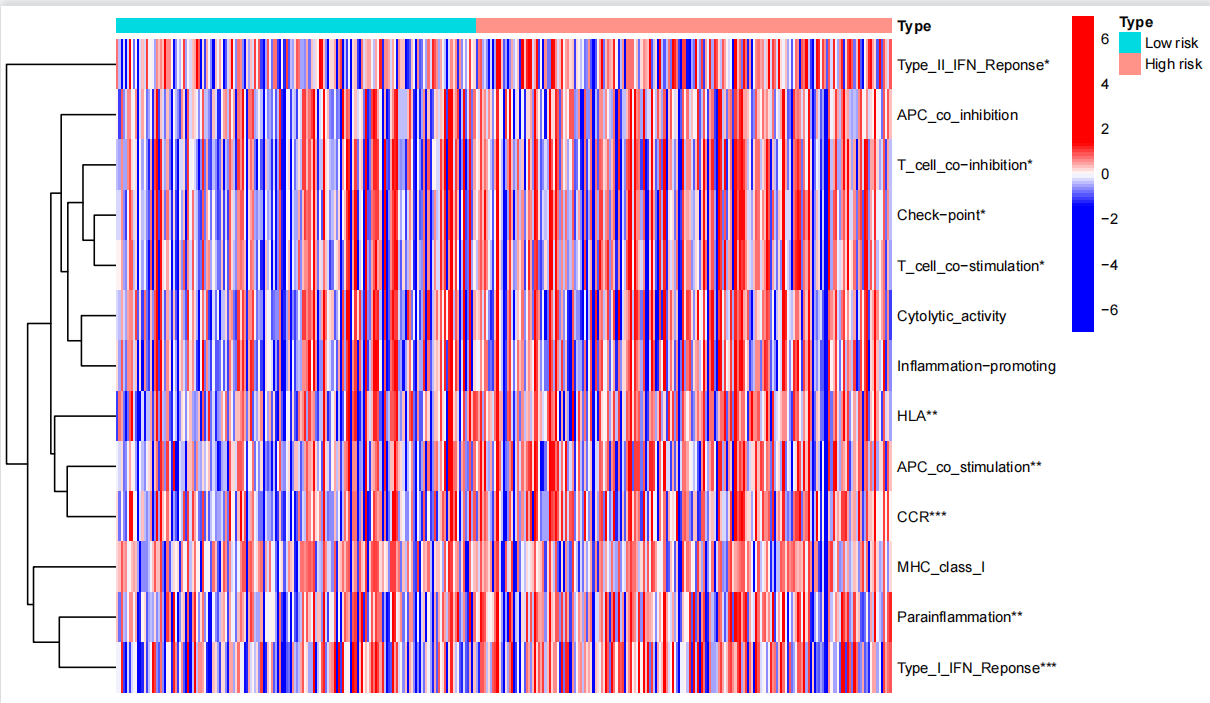


Figure 46. Immune-related Functions in the immune analysis.

As shown in Figure 47, the horizontal axis represents the patients' risk, while the ordinate represents the Institute for Democracy and Electoral Assistance (TIDE) score. A higher score indicates a greater potential for immune evasion, which implies a poorer response to immune therapy in patients.


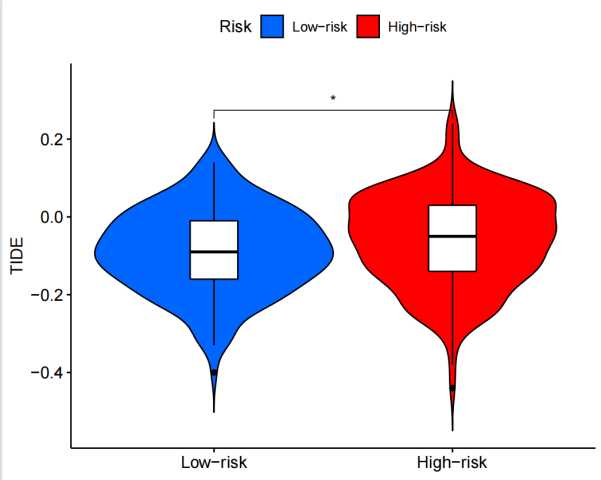


Figure 47. Immune Evasion and Immunotherapy in the immune analysis.

### Dimensionality Reduction

Under the CRGlncRNA Analysis drop-down, click Dimensionality Reduction to open and use the analysis. There is one sub-section the Dimensionality Reduction section:

1. Principle Component Analysis. *Present the ability of the prognostic genes to figure out the high-risk and the low-risk patients.*

The result reveals whether the prognostic model can distinguish the high-risk patients and low-risk patients(Figure 48).


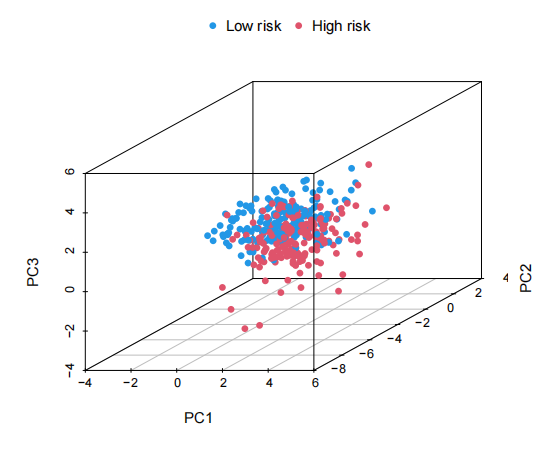


Figure 48. Principle Component Analysis (PCA) in the dimensionality reduction.

### Conjoint Analysis

Under the Conjoint Analysis drop-down, click mRNA-lncRNA-circRNA Analysis to open and use the analysis. The regulatory network is visually presented as follows: mRNAs are represented by blue circles, miRNAs by pink triangles, circRNAs by red diamonds, and lncRNA by green ovals. Interactions between the remaining three types of RNAs and miRNAs are depicted by connecting lines. Notably, when multiple RNAs connect to the same miRNA, it suggests the potential existence of competition among them, vying for the same miRNA.

1. Correlation Analysis. *The KEGG and GO enrichment analysis of each cancer.*

### Differential Analysis

CuPCA presents users with a differential analysis of mRNA, lncRNA, and circRNA, respectively. The sub-menu below this menu contains:

1. mRNA Differential Analysis. *Shows the differential expression condition of mRNA in cancer samples and normal samples.*
2. lncRNA Differential Analysis. *Shows the differential expression condition of lncRNA in cancer samples and normal samples.*
3. circRNA Differential Analysis. *Shows the differential expression condition of circRNA in cancer samples and normal samples.*

According to the differential analysis chart(Figure 49), the heatmap was made. These analyses show users the different expressions of cuproptosis-related mRNA, lncRNA, and circRNA between tumors and normal tissues.


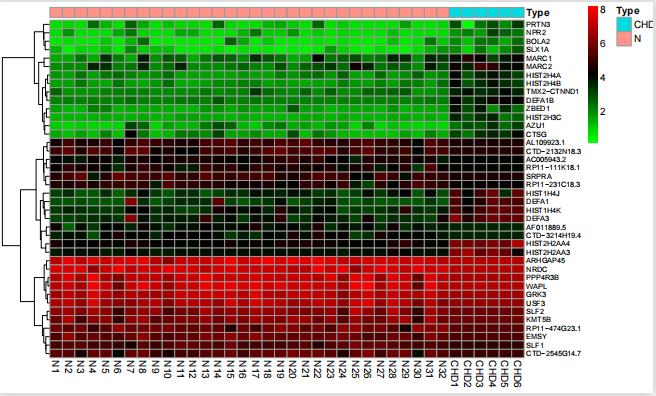


Figure 49. Differential heatmap of CRGs in the Coronary Heart Disease (CHD) cancer.

### Regulatory Internet

mRNA, lncRNA, and circRNA have a relationship with each other through the binding of miRNA, and miRNA is the bridge that brings them together. Thus, in this part, CuPCA shows users the binding condition of miRNA and mRNA, miRNA and lncRNA, and miRNA and circRNA, respectively. The sub-menu below this menu contains:

1. Regulatory Internet. *The regulatory internet of mRNA, lncRNA, and circRNA.*

According to the binding condition presented, a regulatory network is made. It is a visualization of the miRNA binding condition, users can gain the competition result between miRNA and mRNA, miRNA and lncRNA, miRNA and circRNA (Figure 50).


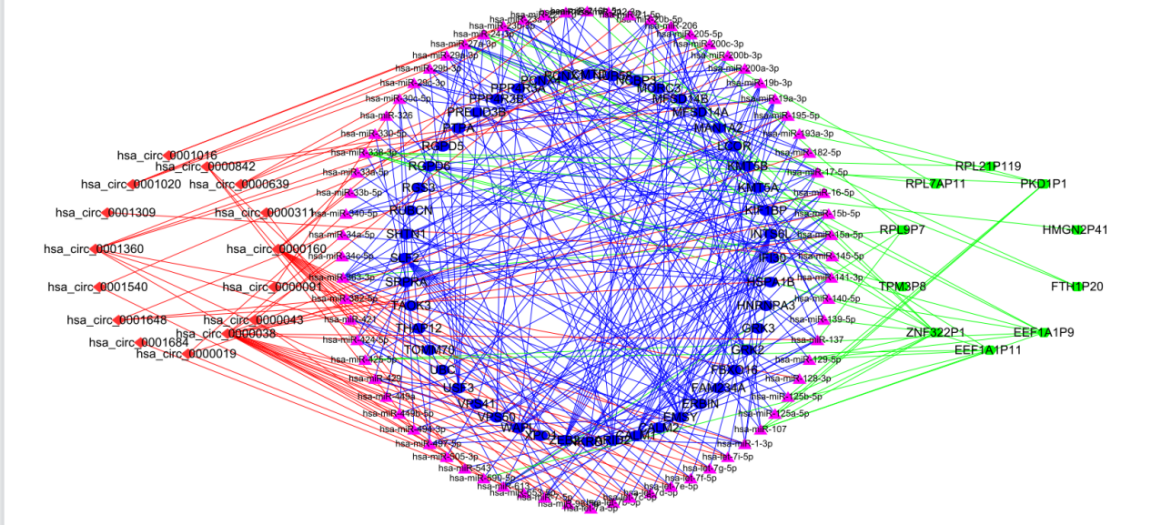


Figure 50. Regulatory Internet of CRGs in the Coronary Heart Disease (CHD) cancer.

### Correlation Analysis

CuPCA provides users with the results of GO enrichment analysis and KEGG enrichment analysis (Figures 51 and 52). Both analysis results present users with a bubble plot and a barplot, which shows the enrichment condition of genes in certain functions and pathways. The sub-menu below this menu contains:

1. KEGG enrichment Analysis. *Perform the KEGG enrichment analysis on the differentially expressed genes.*
2. GO enrichment Analysis. *Perform the GO enrichment analysis on the differentially expressed genes.*


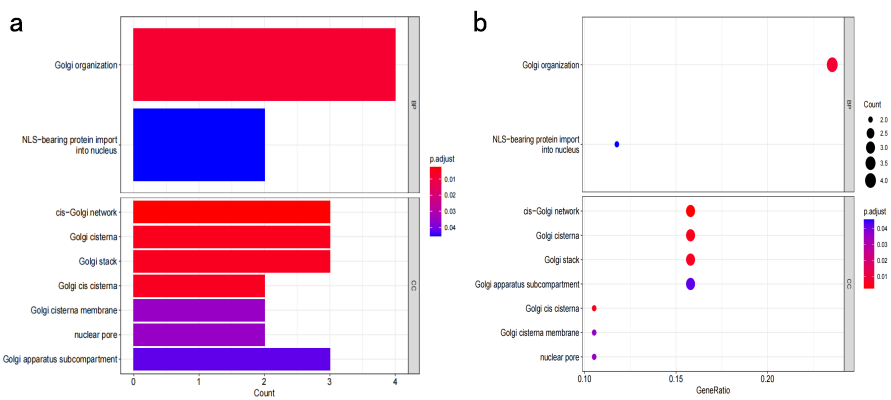


Figure 51. GO analysis of CRGs in the Colorectal Cancer(CRC). (a)Bar plot version of GO results. (b)Bubble plot version of GO results.

*
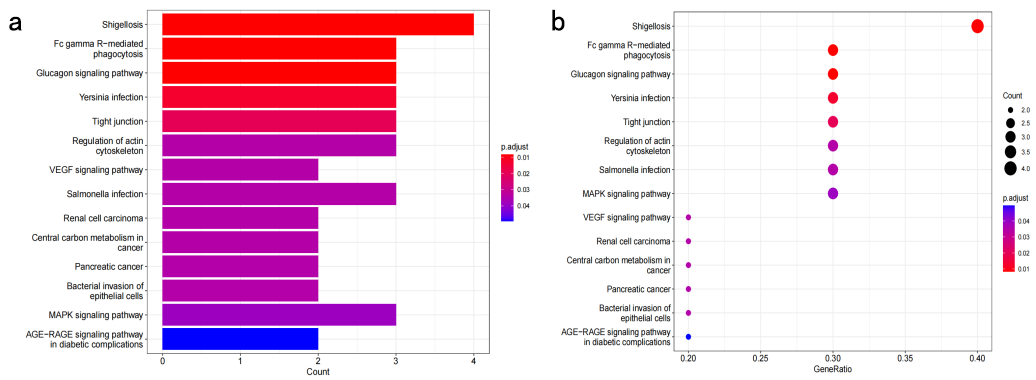
*

Figure 52. KEGG analysis of CRGs in the Coronary Heart Disease(CHD) cancer. (a)Bar plot version of KEGG results.(b)Bubble plot version of KEGG results.

### Feedback

If you have any comments, please email the webmaster at zryuan@bjfu.edu.cn. Your feedback is important to us.

### Working group

| **Name** | **Task/Role** | **Affiliation** |
| --- | --- | --- |
| Yishu Xu | Data Analysis and Database Construction | Beijing Forestry University |
| Zhenshu Ma | Data Analysis and Database Construction | Beijing Forestry University |
| Jiaming Ye | Gene Exploration and Data Analysis | Beijing Forestry University |
| Long Zhang | Art Design | Beijing Forestry University |
| Yuan Chen | Gene Exploration and Raw Data Collection | Beijing Ditan Hospital, Capital Medical University |
| Yajie Wang | Gene Exploration and Raw Data Collection | Beijing Ditan Hospital, Capital Medical University |
| Zhengrong Yuan | Gene Exploration, Raw Data Collection, and Study Supervise | Beijing Forestry University |
