# Supplementary material for: CuPCA: a web server for pan-cancer association analysis of large-scale cuproptosis-related genes
Source: Database (Oxford). 2024 Sep 4;2024:baae075. doi: 10.1093/database/baae075 (PMC11373563; doi:10.1093/database/baae075)
Supplement: baae075_Supp [file baae075_supp.zip › suppl_data/Table S3.docx]

**Table S3. The names of genes that were put into the pan-cancer analysis of Multi-cuproptosis-related genes (CRGs).**

| **Gene Name** | **Description** |
| --- | --- |
| *AANAT* | Aralkylamine N-Acetyltransferase |
| *ABCB10* | ATP Binding Cassette Sub Family B (Mdr/Tap) Member 10 |
| *ABCB11* | ATP Binding Cassette Sub Family B (Mdr/Tap) Member 11 |
| *ABCB4* | ATP Binding Cassette Sub Family B (Mdr/Tap) Member 4 |
| *ABCB6* | ATP Binding Cassette Sub Family B (Mdr/Tap) Member 6 |
| *ABCB7* | ATP Binding Cassette Sub Family B (Mdr/Tap) Member 7 |
| *ABCB8* | ATP Binding Cassette Sub Family B (Mdr/Tap) Member 8 |
| *ACO1* | Aconitase 1 Soluble |
| *ACO2* | Aconitase 2 Mitochondrial |
| *ACP1* | Acid Phosphatase 1 Prostate |
| *ACR* | Acrosin |
| *ADNP* | Activity-Dependent Neuroprotector Homeobox |
| *ALB* | Albumin |
| *ANKRD9* | Ankyrin Repeat Domain 9 |
| *AOC1* | Amine Oxidase Copper Containing 1 |
| *AOC2* | Amine Oxidase Copper Containing 2 |
| *AOC3* | Amine Oxidase Copper Containing 3 |
| *AP1S1* | Adaptor Related Protein Complex 1 Sigma 1 Subunit |
| *AQP1* | Aquaporin 1 |
| *ARF1* | ADP-Ribosylation Factor 1 |
| *ATOX1* | Antioxidant 1 Copper Chaperone |
| *ATP13A2* | ATPase Cation Transporting 13A2 |
| *ATP6AP1* | ATPase 6 Associated Protein 1 |
| *ATP7A* | ATPase Cu++ Transporting Alpha Polypeptide |
| *ATP7B* | ATPase Cu++ Transporting Beta Polypeptide |
| *ATP8B1* | ATPase Class I Type 8B Member 1 |
| *BECN1* | Beclin 1 |
| *CCDC115* | Coiled-Coil Domain Containing 115 |
| *CCDC22* | Coiled-Coil Domain Containing 22 |
| *CCS* | Copper Chaperone For Superoxide Dismutase |
| *CD44* | Cd44 Molecule |
| *CDKN1A* | Cyclin Dependent Kinase Inhibitor 1A |
| *CDKN2A* | Cyclin Dependent Kinase Inhibitor 2A |
| *CDKN3* | Cyclin Dependent Kinase Inhibitor 3 |
| *CHD8* | Coiled-Coil Helix Coiled Coil Helix Domain Containing 8 |
| *CIAO1* | Cellular Iron Arsenite Oxidase 1 |
| *CIAO2A* | Cellular Iron Arsenite Oxidase 2A |
| *CIAO2B* | Cellular Iron Arsenite Oxidase 2B |
| *CIAPIN1* | Cellular Inhibitor Of Apoptosis Protein 1 |
| *COA6* | Cox Assembly Factor 6 |
| *COMMD1* | Copii Coat Complex Component 1 |
| *COX11* | Cytochrome C Oxidase Assembly Factor 11 |
| *COX17* | Cytochrome C Oxidase Assembly Factor 17 |
| *COX18* | Cytochrome C Oxidase Assembly Factor 18 |
| *COX19* | Cytochrome C Oxidase Assembly Factor 19 |
| *COX4I1* | Cytochrome C Oxidase Subunit IV Isoform 1 |
| *COX5A* | Cytochrome C Oxidase Subunit 5A |
| *COX5B* | Cytochrome C Oxidase Subunit 5B |
| *COX6A1* | Cytochrome C Oxidase Subunit 6A1 |
| *COX6B1* | Cytochrome C Oxidase Subunit 6B1 |
| *COX7A2* | Cytochrome C Oxidase Subunit 7A2 |
| *COX7A2L* | Cytochrome C Oxidase Subunit 7A2L |
| *COX7B* | Cytochrome C Oxidase Subunit 7B |
| *COX7C* | Cytochrome C Oxidase Subunit 7C |
| *COX8A* | Cytochrome C Oxidase Subunit 8A |
| *CP* | Cytochrome P450 |
| *CUTA* | Cutinase A |
| *CUTC* | Cutinase C |
| *DAXX* | Death Associated Protein X Linked |
| *DBH* | Dopamine Beta Hydroxylase |
| *DBT* | Dopamine Beta Hydroxylase |
| *DLAT* | Dihydrolipoamide S Acetyltransferase |
| *DLD* | Dihydrolipoamide Dehydrogenase |
| *DLST* | Dihydrolipoamide S Transferase |
| *DPYD* | Dipeptidyl Peptidase D |
| *F5* | Factor V |
| *F8* | Factor V8 |
| *FDX1* | Flavodoxin 1 |
| *FDX2* | Flavodoxin 2 |
| *FDXR* | Flavodoxin Reductase |
| *FXN* | Glutaredoxin |
| *GCSH* | Glutathione Synthetase |
| *GLRX5* | Glutaredoxin 5 |
| *GLS* | Glutamine Synthetase |
| *HAMP* | Hepcidin Antimicrobial Peptide |
| *HEPH* | Hephestin |
| *HSCB* | HscB Mitochondrial Iron-Sulfur Cluster Cochaperone |
| *HSPA8* | Heat Shock Protein Family A (Hsp70) Member 8 |
| *HSPA9* | Heat Shock Protein Family A (Hsp70) Member 9 |
| *IBA57* | Inhibitor Of Apoptosis Protein 57 |
| *IDH1* | Isocitrate Dehydrogenase (NADP(+)) 1 |
| *IDH2* | Isocitrate Dehydrogenase (NADP(+)) 2 |
| *ISCA1* | Iron-Sulfur Cluster Assembly Protein 1 |
| *ISCA2* | Iron-Sulfur Cluster Assembly Protein 2 |
| *ISCU* | Iron-Sulfur Cluster Assembly Protein X |
| *IVD* | Inositol Dehydrogenase |
| *LIAS* | Lipoic Acid Synthetase |
| *LIPT1* | Lipid Transfer Protein 1 |
| *LIPT2* | Lipid Transfer Protein 2 |
| *LOXL1* | Lysyl Oxidase Like 1 |
| *LOXL4* | Lysyl Oxidase Like 4 |
| *MPI* | Mitochondrial Pyruvate Carrier Protein |
| *MT-CO1* | Mitochondrially Encoded Cytochrome C Oxidase I |
| *MT-CO2* | Mitochondrially Encoded Cytochrome C Oxidase II |
| *MT-CO3* | Mitochondrially Encoded Cytochrome C Oxidase III |
| *MT1A* | Metallothionein 1A |
| *MT1B* | Metallothionein1B |
| *MT1E* | Metallothionein1E |
| *MT1F* | Metallothionein1F |
| *MT1G* | Metallothionein1G |
| *MT1H* | Metallothionein1H |
| *MT1HL1* | Metallothionein1H Like 1 |
| *MT1X* | Metallothionein1X |
| *MT2A* | Metallothionein2A |
| *MT3* | Metallothionein3 |
| *MT4* | Metallothionein4 |
| *MTF1* | Mammalian Target Of Rapamycin Complex 1 (Alpha) |
| *NDOR1* | NADPH Dependent Diflavin Oxidoreductase 1 |
| *NFE2L2* | NFE2 Like BZIP Transcription Factor 2 |
| *NFS1* | NFS1 Cysteine Desulfurase |
| *NFU1* | NFU1 Iron-Sulfur Cluster Scaffold |
| *NLRP3* | NLR Family Pyrin Domain Containing 3 |
| *NUBP1* | NUBP iron-sulfur cluster assembly factor 1, cytosolic |
| *NUBP2* | NUBP Iron-Sulfur Cluster Assembly Factor 2, Cytosolic |
| *NUBPL* | NUBP Iron-Sulfur Cluster Assembly Factor, Mitochondrial |
| *OR5AR1* | Olfactory Receptor Family 5 Subfamily A Member 1 |
| *PAM* | Peptidylglycine Alpha Amidating Monooxygenase |
| *PDHA1* | Pyruvate Dehydrogenase Complex Component A1 |
| *PDHB* | Pyruvate Dehydrogenase Complex Component B |
| *PRND* | Proline Rich Nuclear Receptor Coactivator 1 |
| *PRNP* | Prion Protein |
| *SCO1* | Synthesis Of Cytochrome C Oxidase 1 |
| *SDHA* | Succinate Dehydrogenase Complex Flavoprotein Subunit A |
| *SDHB* | Succinate Dehydrogenase Complex Iron-Sulfur Subunit B |
| *SDHC* | Succinate Dehydrogenase Complex Subunit C |
| *SDHD* | Succinate Dehydrogenase Complex Subunit D |
| *SLC11A2* | Solute Carrier Family 11 Member 2 |
| *SLC25A3* | Solute Carrier Family 25 Member 3 |
| *SLC31A1* | Solute Carrier Family 31 Member 1 |
| *SLC31A2* | Solute Carrier Family 31 Member 2 |
| *SLC33A1* | Solute Carrier Family 33 Member 1 |
| *SNCA* | Synuclein Alpha |
| *SNCB* | Synuclein Beta |
| *SOD1* | Superoxide Dismutase 1 |
| *SOD2* | Superoxide Dismutase 2 |
| *SOD3* | Superoxide Dismutase 3 |
| *STEAP2* | Steap Family Member 2 |
| *STEAP4* | Steap Family Member 4 |
| *SUMF1* | Sulfatase Modifying Factor 1 |
| *TFRC* | Transferrin Receptor |
| *TMEM199* | Transmembrane Protein 199 |
| *TPI1* | Triosephosphate Isomerase 1 |
| *XIAP* | X-Linked Inhibitor Of Apoptosis |
