# Supplementary material for: CuPCA: a web server for pan-cancer association analysis of large-scale cuproptosis-related genes
Source: Database (Oxford). 2024 Sep 4;2024:baae075. doi: 10.1093/database/baae075 (PMC11373563; doi:10.1093/database/baae075)
Supplement: baae075_Supp [file baae075_supp.zip › suppl_data/Table S1.docx]

**Table S1. The names of cuproptosis-related genes (CRGs) that participated in the pan-cancer analysis process in the CuPCA.**

| **Gene Name** | **Description** |
| --- | --- |
| *AANAT* | Aralkylamine N-Acetyltransferase |
| *AARSD1* | Alanyl-Trna Synthetase Domain Containing 1 |
| *ABCA12* | ATP Binding Cassette Sub Family B (Mdr/Tap) Member 12 |
| *ABCB10* | ATP Binding Cassette Sub Family B (Mdr/Tap) Member 10 |
| *ABCB11* | ATP Binding Cassette Sub Family B (Mdr/Tap) Member 11 |
| *ABCB4* | ATP Binding Cassette Sub Family B (Mdr/Tap) Member 4 |
| *ABCB6* | ATP Binding Cassette Sub Family B (Mdr/Tap) Member 6 |
| *ABCB7* | ATP Binding Cassette Sub Family B (Mdr/Tap) Member 7 |
| *ABCB8* | ATP Binding Cassette Sub Family B (Mdr/Tap) Member 8 |
| *ABCC1* | ATP Binding Cassette Subfamily C Member 1 |
| *ABHD10* | Abhydrolase Domain Containing 10 |
| *ABHD14A* | Abhydrolase Domain Containing 14A |
| *ABHD14B* | Abhydrolase Domain Containing 14B |
| *ABL2* | ABL Proto-Oncogene 2, Non-Receptor Tyrosine Kinase |
| *ACAA1* | Acetyl-CoA Acyltransferase 1 |
| *ACAD10* | Acyl-CoA Dehydrogenase Family Member 10 |
| *ACAD8* | Acyl-CoA Dehydrogenase Family Member 8 |
| *ACADS* | Acyl-CoA Dehydrogenase Short Chain |
| *ACAP2* | ArfGAP With Coiled-Coil, Ankyrin Repeat And PH Domains 2 |
| *ACAT1* | Acetyl-CoA Acetyltransferase 1 |
| *ACBD4* | Acyl-CoA Binding Domain Containing 4 |
| *ACO1* | Aconitase 1 Soluble |
| *ACO2* | Aconitase 2 Mitochondrial |
| *ACOT12* | Acyl-CoA Thioesterase 12 |
| *ACOT13* | Acyl-CoA Thioesterase 13 |
| *ACOT8* | Acyl-CoA Thioesterase 8 |
| *ACP1* | Acid Phosphatase 1 Prostate |
| *ACP6* | Acid Phosphatase 6 Prostate |
| *ACR* | Acrosin |
| *ACTN1* | Alpha-Actinin-1 |
| *ACTR2* | Alpha-Actinin-2 |
| *ACTR3* | Alpha-Actinin-3 |
| *ACY1* | Acylaminoacyl Peptide Hydrolase |
| *ACYP2* | Acylphosphatase 2 |
| *ADAM12* | A Disintegrin And Metalloproteinase Domain 12 |
| *ADAM17* | A Disintegrin And Metalloproteinase Domain 17 |
| *ADAM19* | A Disintegrin And Metalloproteinase Domain 19 |
| *ADAMTS12* | ADAM Metallopeptidase With Thrombospondin Type 1 Motif 12 |
| *ADAMTS14* | ADAM Metallopeptidase With Thrombospondin Type 1 Motif 14 |
| *ADAMTS2* | ADAM Metallopeptidase With Thrombospondin Type 1 Motif 2 |
| *ADAR* | Adenosine Deaminase RNA Specific |
| *ADCY3* | Adenylate Cyclase 3 |
| *ADCY7* | Adenylate Cyclase 7 |
| *ADI1* | Adenylate Kinase Isoenzyme 1 |
| *ADNP* | Activity Dependent Neuroprotector Homeobox |
| *ADGRF4* | Adhesion G Protein-Coupled Receptor F4 |
| *AGFG2* | Arf-Gap With Fg Repeats 2 |
| *AHNAK* | Neuroblast Differentiation-Associated Protein |
| *AHNAK2* | Neuroblast Differentiation-Associated Protein 2 |
| *AIFM1* | Apoptosis-Inducing Factor Mitochondrion-Associated 1 |
| *AKAP13* | A-Kinase Anchoring Protein 13 |
| *ALB* | Albumin |
| *ALDH1L2* | Aldehyde Dehydrogenase 1 Family Member L2 |
| *ALDH5A1* | Aldehyde Dehydrogenase 5 Family Member A1 |
| *ALG14* | Asparagine-Linked Glycosylation 14 |
| *ALG5* | Asparagine-Linked Glycosylation 5 |
| *ALG9* | Asparagine-Linked Glycosylation 9 |
| *ALKBH7* | AlkB Homolog 7 |
| *ALOX5AP* | Arachidonate 5-Lipoxygenase-Activating Protein |
| *AMT* | Ammonium Transporter |
| *ANAPC13* | Anaphase Promoting Complex Subunit 13 |
| *ANAPC16* | Anaphase Promoting Complex Subunit 16 |
| *ANKLE2* | Ankyrin Repeat And Lem Domain-Containing Protein 2 |
| *ANKRD36BP1* | Ankyrin Repeat Domain 36B Binding Protein 1 |
| *ANKRD50* | Ankyrin Repeat Domain 50 |
| *ANKRD9* | Ankyrin Repeat Domain 9 |
| *ANLN* | Anillin |
| *ANXA1* | Annexin A1 |
| *ANXA2P1* | Annexin A2 Pseudogene 1 |
| *ANXA2P2* | Annexin A2 Pseudogene 2 |
| *ANXA8L2* | Annexin A8-Like Protein 2 |
| *AOC1* | Amine Oxidase Copper Containing 1 |
| *AOC2* | Amine Oxidase Copper Containing 2 |
| *AOC3* | Amine Oxidase Copper Containing 3 |
| *AP1S1* | Adaptor Related Protein Complex 1 Sigma 1 Subunit |
| *AP4E1* | Adaptor Protein Complex 4 Subunit Epsilon 1 |
| *APCDD1L* | APC Down-Regulated 1 Like |
| *APEH* | Acylpeptide Hydrolase |
| *APIP* | Apaf1 Interacting Protein |
| *APOA1* | Apolipoprotein A1 |
| *APOBEC3A* | Apolipoprotein B Mrna Editing Enzyme Catalytic Subunit 3A |
| *APOBEC3C* | Apolipoprotein B Mrna Editing Enzyme Catalytic Subunit 3C |
| *APOM* | Apolipoprotein M |
| *APOO* | Apolipoprotein O |
| *AQP1* | Aquaporin 1 |
| *AQP11* | Aquaporin 11 |
| *ARF1* | ADP-Ribosylation Factor 1 |
| *ARHGAP11B* | Rho GTPase Activating Protein 11B |
| *ARHGAP23* | Rho GTPase Activating Protein 23 |
| *ARID1A* | At-Rich Interactive Domain 1A |
| *ARL10* | ADP-Ribosylation Factor-Like 10 |
| *ARL4C* | ADP-Ribosylation Factor-Like 4C |
| *ARL6IP4* | ADP-Ribosylation Factor-Like 6 Interacting Protein 4 |
| *ARNTL2* | Aryl Hydrocarbon Receptor Nuclear Translocator Like 2 |
| *ARPC2* | Actin Related Protein 2/3 Complex Subunit 2 |
| *ARSI* | Arylsulfatase I |
| *ASAM* | Aralkylamine N-Acetyltransferase |
| *ASAP1* | Arf-Gap With Sh3 Domain |
| *ASPSCR1* | Alveolar Soft Part Sarcoma Chromosome Region, Candidate 1 |
| *ASXL2* | Additional Sex Combs Like 2 |
| *ATF7IP* | Activating Transcription Factor 7 Interacting Protein |
| *ATOX1* | Atoxin 1 |
| *ATP10D* | ATPase Phospholipid Transporting 10D |
| *ATP13A2* | ATPase Family Aaa Domain Containing 13A2 |
| *ATP2B4* | ATPase, Ca++ Transporting, Plasma Membrane 4 |
| *ATP5A1* | ATP Synthase F1 Subunit Alpha 1 |
| *ATP5B* | ATP Synthase F1 Subunit Beta |
| *ATP5C1* | ATP Synthase F1 Subunit Gamma 1 |
| *ATP5D* | ATP Synthase F1 Subunit Delta |
| *ATP5F1* | ATP Synthase F1 Subunit Alpha 1 |
| *ATP5G1* | ATP Synthase F1 Subunit Gamma 1 |
| *ATP5G2* | ATP Synthase F1 Subunit Gamma 2 |
| *ATP5G3* | ATP Synthase F1 Subunit Gamma 3 |
| *ATP5H* | ATP Synthase F1 Subunit Epsilon |
| *ATP5I* | ATP Synthase F1 Subunit I |
| *ATP5J* | ATP Synthase F1 Subunit J |
| *ATP5L* | ATP Synthase F1 Subunit L |
| *ATP5MK* | ATP Synthase Membrane Subunit K |
| *ATP5O* | ATP Synthase F1 Subunit O |
| *ATP5S* | ATP Synthase F1 Subunit S |
| *ATP6AP1* | ATPase 6 Associated Protein 1 |
| *ATP7A* | ATPase Copper Transporting Alpha |
| *ATP7B* | ATPase Copper Transporting Beta |
| *ATP8B1* | ATPase Phospholipid Transporting 8B1 |
| *ATPAF1* | ATP Synthase Mitochondrial F1 Complex Assembly Factor 1 |
| *ATPAF2* | ATP Synthase Mitochondrial F1 Complex Assembly Factor 2 |
| *ATPIF1* | ATPase Inhibitory Factor 1 |
| *ATXN1L* | Ataxin 1-Like |
| *BACH1* | Btb Domain And Cnc Homolog 1 |
| *BARD1* | Brca1 Associated Ring Domain 1 |
| *BAT2L2* | Hla-B Associated Transcript 2-Like Protein 2 |
| *BAZ1A* | Bromodomain Adjacent To Zinc Finger Domain 1A |
| *BAZ2A* | Bromodomain Adjacent To Zinc Finger Domain 2A |
| *BCKDHB* | Branched Chain Keto Acid Dehydrogenase E1 Subunit Beta |
| *BCL9L* | B-Cell Cll/Lymphoma 9-Like |
| *BCS1L* | Bcs1-Like Protein |
| *BECN1* | Beclin 1 |
| *BICD2* | Bicaudal D Homolog 2 |
| *BLOC1S1* | Biogenesis Of Lysosomal Organelles Complex 1 Subunit 1 |
| *BMP1* | Bone Morphogenetic Protein 1 |
| *BMP2K* | Bone Morphogenetic Protein 2 Kinase |
| *BNIP1* | Bcl2/Adenovirus E1B 19Kda Protein-Interacting Protein 1 |
| *BPHL* | Biphenyl Hydrolase-Like Protein |
| *BRCA2* | Breast Cancer 2, Early Onset |
| *BRIP1* | Brca1 Interacting Protein C-Terminal Helicase 1 |
| *BRP44* | Brefeldin A-Resistant Guanine Nucleotide Exchange Factor 2 |
| *BRP44L* | Brefeldin A Resistance Protein 44-Like |
| *BRWD3* | Bromodomain And Wd Repeat Domain Containing 3 |
| *BUD23* | BUD23 RRNA Methyltransferase And Ribosome Maturation Factor |
| *C10orf12* | Chromosome 10 Open Reading Frame 12 |
| *C10orf32* | Chromosome 10 Open Reading Frame 32 |
| *C10orf55* | Chromosome 10 Open Reading Frame 55 |
| *C11orf1* | Chromosome 11 Open Reading Frame 1 |
| *C11orf51* | Chromosome 11 Open Reading Frame 51 |
| *C11orf71* | Chromosome 11 Open Reading Frame 71 |
| *C12orf10* | Chromosome 12 Open Reading Frame 10 |
| *C12orf62* | Chromosome 12 Open Reading Frame 62 |
| *C12orf70* | Chromosome 12 Open Reading Frame 70 |
| *C12orf73* | Chromosome 12 Open Reading Frame 73 |
| *C14orf156* | Chromosome 14 Open Reading Frame 156 |
| *C14orf2* | Chromosome 14 Open Reading Frame 2 |
| *C14orf68* | Chromosome 14 Open Reading Frame 68 |
| *C15orf40* | Chromosome 15 Open Reading Frame 40 |
| *C15orf61* | Chromosome 15 Open Reading Frame 61 |
| *C17orf108* | Chromosome 17 Open Reading Frame 108 |
| *C17orf61* | Chromosome 17 Open Reading Frame 61 |
| *C17orf81* | Chromosome 17 Open Reading Frame 81 |
| *C17orf89* | Chromosome 17 Open Reading Frame 89 |
| *C18orf32* | Chromosome 18 Open Reading Frame 32 |
| *C18orf54* | Chromosome 18 Open Reading Frame 54 |
| *C19orf42* | Chromosome 19 Open Reading Frame 42 |
| *C19orf55* | Chromosome 19 Open Reading Frame 55 |
| *C19orf56* | Chromosome 19 Open Reading Frame 56 |
| *C19orf70* | Chromosome 19 Open Reading Frame 70 |
| *C1orf38* | Chromosome 1 Open Reading Frame 38 |
| *C1orf56* | Chromosome 1 Open Reading Frame 56 |
| *C1orf66* | Chromosome 1 Open Reading Frame 66 |
| *C1orf84* | Chromosome 1 Open Reading Frame 84 |
| *C20orf132* | Chromosome 20 Open Reading Frame 132 |
| *C20orf30* | Chromosome 20 Open Reading Frame 30 |
| *C20orf7* | Chromosome 20 Open Reading Frame 7 |
| *C21orf33* | Chromosome 21 Open Reading Frame 33 |
| *C21orf57* | Chromosome 21 Open Reading Frame 57 |
| *C22orf30* | Chromosome 22 Open Reading Frame 30 |
| *C22orf32* | Chromosome 22 Open Reading Frame 32 |
| *C2orf64* | Chromosome 2 Open Reading Frame 64 |
| *C2orf7* | Chromosome 2 Open Reading Frame 7 |
| *C3orf23* | Chromosome 3 Open Reading Frame 23 |
| *C3orf71* | Chromosome 3 Open Reading Frame 71 |
| *C3orf74* | Chromosome 3 Open Reading Frame 74 |
| *C3orf75* | Chromosome 3 Open Reading Frame 75 |
| *C4orf52* | Chromosome 4 Open Reading Frame 52 |
| *C5AR1* | Chromosome 5 Area Specific Repeats 1 |
| *C6orf108* | Chromosome 6 Open Reading Frame 108 |
| *C6orf130* | Chromosome 6 Open Reading Frame 130 |
| *C6orf136* | Chromosome 6 Open Reading Frame 136 |
| *C6orf150* | Chromosome 6 Open Reading Frame 150 |
| *C6orf167* | Chromosome 6 Open Reading Frame 167 |
| *C6orf203* | Chromosome 6 Open Reading Frame 203 |
| *C6orf226* | Chromosome 6 Open Reading Frame 226 |
| *C7orf30* | Chromosome 7 Open Reading Frame 30 |
| *C7orf55* | Chromosome 7 Open Reading Frame 55 |
| *C7orf59* | Chromosome 7 Open Reading Frame 59 |
| *C9orf103* | Chromosome 9 Open Reading Frame 103 |
| *C9orf110* | Chromosome 9 Open Reading Frame 110 |
| *C9orf123* | Chromosome 9 Open Reading Frame 123 |
| *C9orf23* | Chromosome 9 Open Reading Frame 23 |
| *C9orf53* | Chromosome 9 Open Reading Frame 53 |
| *CA5A* | Carbonic Anhydrase V A |
| *CABC1* | Carbonic Anhydrase B, Carbonate Dehydratase I |
| *CALML5* | Calmyrin Like Protein 5 |
| *CAMSAP1* | Calmodulin Regulated Spectrin Associated Protein 1 |
| *CAP1* | Adenylyl Cyclase Associated Protein 1 |
| *CARKD* | Carbamoyl Phosphate Synthetase 1, Mitochrondrial Like Protein |
| *CBL* | Cbl Proto-Oncogene |
| *CCDC115* | Coiled Coil Domain Containing 115 |
| *CCDC12* | Coiled Coil Domain Containing 22 |
| *CCDC22* | Coiled Coil Domain Containing Protein 22 |
| *CCDC56* | Coiled Coil Domain Containing Protein 56 |
| *CCDC72* | Coiled Coil Domain Containing Protein 72 |
| *CCNB1IP1* | Cyclin B1 Interacting Protein 1, E3 Ubiquitin Protein Ligase |
| *CCN4* | Cellular Communication Network Factor 4 |
| *CCNG1* | Cyclin G1 |
| *CCNT1* | Cyclin T1 |
| *CCR4* | Chemokine (Cc Motif) Receptor Type 4 |
| *CCR8* | Chemokine (Cc Motif) Receptor Type 8 |
| *CCS* | Copper Chaperone For Superoxide Dismutase |
| *CD101* | CD101 Molecule (B Cell Antigen Cd3Like) |
| *CD109* | CD109 Molecule (Semaphorin7A) |
| *CD274* | CD274 Molecule (Also Known As Pdl1) |
| *CD300E* | CD300E Molecule (Irp2) |
| *CD320* | CD320 Molecule (Also Known As Cdcp1) |
| *CD44* | CD44 Molecule |
| *CD80* | CD80 Molecule (Also Known As B71) |
| *CD84* | CD84 Molecule (Also Known As Ly54) |
| *CD86* | CD86 Molecule (Also Known As B72) |
| *CDC42SE1* | CDC42 Small Effector Protein 1 |
| *CDH11* | Cadherin 11 |
| *CDKL5* | Cyclin Dependent Kinase Like 5 |
| *CDKN1A* | Cyclin Dependent Kinase Inhibitor 1A |
| *CDKN2A* | Cyclin Dependent Kinase Inhibitor 2A |
| *CDKN2B* | Cyclin Dependent Kinase Inhibitor 2B |
| *CDKN2BAS* | Cdkn2B Antisense RNA 1 |
| *CDKN3* | Cyclin Dependent Kinase Inhibitor 3 |
| *CENPI* | Centromere Protein I |
| *CEP110* | Centrosomal Protein 110 |
| *CEP135* | Centrosomal Protein 135 |
| *CEP350* | Centrosomal Protein 350 |
| *CEP97* | Centrosomal Protein 97 |
| *CHCHD10* | Coiled-Coil-Helix-Coiled-Coil-Helix Domain Containing 10 |
| *CHCHD2* | Coiled-Coil-Helix-Coiled-Coil-Helix Domain Containing 2 |
| *CHCHD4* | Coiled-Coil-Helix-Coiled-Coil-Helix Domain Containing 4 |
| *CHCHD8* | Coiled-Coil-Helix-Coiled-Coil-Helix Domain Containing 8 |
| *CHD8* | Chromodomain Helicase DNA Binding Protein 8 |
| *CHMP2A* | Charged Multivesicular Body Protein 2A |
| *CHPT1* | Choline Phosphotransferase 1 |
| *CHST11* | Carbohydrate Sulfotransferase 11 |
| *CHSY1* | Cholinergic Syncythemia, Autosomal Recessive 1 |
| *CHSY3* | Cholinergic Syncythemia, Autosomal Recessive 3 |
| *CIAO1* | Cellular Iron Arsenite Oxidase 1 |
| *CIAO2A* | Cellular Iron Arsenite Oxidase 2A |
| *CIAO2B* | Cellular Iron Arsenite Oxidase 2B |
| *CIAPIN1* | Cellular Inhibitor Of Apoptosis Protein 1 |
| *CISD3* | Ciliary Ionic Substance Discharger 3 |
| *CLCA2* | Chloride Channel Accessory 2 |
| *CLEC2B* | C-Type Lectin Domain Family 2 Member B |
| *CLEC5A* | C-Type Lectin Domain Family 5 Member A |
| *CLEC7A* | C-Type Lectin Domain Family 7 Member A |
| *CLOCK* | Circadian Locomotor Output Cycles Kaput |
| *CLPP* | Cationic Lipid Binding Protein |
| *CLSPN* | Claspin |
| *CLYBL* | Catalase Like Gene |
| *CMC1* | Chemokine (C C Motif) Mediated Cellular Signaling 1 |
| *CMIP* | Cytokine Mitogenic Protein |
| *CMKLR2* | Chemerin Chemokine-Like Receptor 2 |
| *CMTM1* | Cmt1A/T Repeat Expansion Family Member 1 |
| *CNPY2* | Canopy Protein Y2 |
| *COA6* | Cox Assembly Factor 6 |
| *COL10A1* | Collagen Type X Alpha 1 Chain |
| *COL11A1* | Collagen Type XI Alpha 1 Chain |
| *COL1A1* | Collagen Type I Alpha 1 Chain |
| *COL24A1* | Collagen Type XXIV Alpha 1 Chain |
| *COL5A1* | Collagen Type V Alpha 1 Chain |
| *COL5A2* | Collagen Type V Alpha 2 Chain |
| *COL8A2* | Collagen Type VIII Alpha 2 Chain |
| *COMMD1* | Copper Metabolism Domain Containing 1 |
| *COPE* | Coatomer Subunit Epsilon |
| *COQ10A* | Coenzyme Q10 Adenylyltransferase |
| *COQ3* | Coenzyme Q3 |
| *COQ4* | Coenzyme Q4 |
| *COQ5* | Coenzyme Q5 |
| *COQ9* | Coenzyme Q9 |
| *CORO1C* | Coronin 1C |
| *COX11* | Cytochrome C Oxidase Assembly Factor 11 |
| *COX16* | Cytochrome C Oxidase Assembly Factor 16 |
| *COX17* | Cytochrome C Oxidase Assembly Factor 17 |
| *COX18* | Cytochrome C Oxidase Assembly Factor 18 |
| *COX19* | Cytochrome C Oxidase Assembly Factor 19 |
| *COX4I1* | Cytochrome C Oxidase Subunit IV Isoform 1 |
| *COX5A* | Cytochrome C Oxidase Subunit 5A |
| *COX5B* | Cytochrome C Oxidase Subunit 5B |
| *COX6A1* | Cytochrome C Oxidase Subunit 6A1 |
| *COX6B1* | Cytochrome C Oxidase Subunit 6B1 |
| *COX7A2* | Cytochrome C Oxidase Subunit 7A2 |
| *COX7A2L* | Cytochrome C Oxidase Subunit 7A2L |
| *COX7B* | Cytochrome C Oxidase Subunit 7B |
| *COX7C* | Cytochrome C Oxidase Subunit 7C |
| *COX8A* | Cytochrome C Oxidase Subunit 8A |
| *CP* | Cytochrome P450 |
| *CREB1* | Cyclic Adenosine Monophosphate Responsive Nuclear Factor Creb |
| *CREBBP* | C/Ebp Binding Protein |
| *CRLF3* | Caspase Recruitment Like Factor 3 |
| *CRLS1* | Cardiac Rhabdomyosarcoma Like Syndrome 1 |
| *CRYL1* | Corynelbinder Protein 1 |
| *CSDAP1* | Copii Coat GTPase Activating Protein 1 |
| *CSF2RB* | Colony Stimulating Factor 2 Receptor Beta |
| *CSGALNACT2* | Commissural Ganglioside Induced Neuroprotection And Ataxia Linked Tauopathy 2 |
| *CSNK1G1* | Casein Kinase 1, Gamma 1 |
| *CTDSP2* | Ctd Superfamily Member 2 |
| *CTHRC1* | Corticotropin Releasing Hormone Receptor Corepressor 1 |
| *CTTNBP2NL* | Cortactin Binding Protein 2 Non Lethal Variant |
| *CUTA* | Cutinase A |
| *CUTC* | Cutinase C |
| *CWC15* | Cilia And Wnt Signaling Center Protein 15 |
| *CYB561D2* | Cytochrome B 561 Domain Containing 2 |
| *CYBB* | Cytochrome B C1 Complex Subunit Beta |
| *C8orf82* | Chromosome 8 Open Reading Frame 82 |
| *DAK* | Dihydroxyacetone Kinase |
| *DAPP1* | Dopamine Precursor Protein 1 |
| *DAXX* | Death Associated Protein X Linked |
| *DBH* | Dopamine Beta Hydroxylase |
| *DBT* | Dopamine Beta Hydroxylase |
| *DCI* | Dicer Control Region Interacting Protein |
| *DCLRE1B* | Dnaj Homolog Subfamily C Member 1B |
| *DCXR* | Doublecortin Receptor (X Linked) |
| *DDT* | Dihydrodiol Dehydrogenase |
| *DDTL* | Dihydrodiol Dehydrogenase Like |
| *DECR1* | DNA Endonuclease/Exonuclease/Phosphatase Family Member 1 |
| *DECR2* | DNA Endonuclease/Exonuclease/Phosphatase Family Member 2 |
| *DENND2C* | Denn/Madd Domain Containing Protein 2C |
| *DGKA* | Diacylglycerol Kinase Alpha |
| *DHODH* | Dihydroorotate Dehydrogenase |
| *DHPS* | Dihydropyrimidinase |
| *DHRS12* | Dihydrofolate Reductase Simulator 12 |
| *DHRS7B* | Dihydrofolate Reductase Simulator 7B |
| *DHRSX* | Dihydrofolate Reductase Simulator X |
| *DHX8* | Dihydrofolate Induced Deaminase |
| *DLAT* | Dihydrolipoamide S Acetyltransferase |
| *DLD* | Dihydrolipoamide Dehydrogenase |
| *DLST* | Dihydrolipoamide S Transferase |
| *DNAJC13* | Dnaj (Hsp40) Homolog, Subfamily C, Member 13 |
| *DNAJC19* | Dnaj (Hsp40) Homolog, Subfamily C, Member 19 |
| *DNAJC30* | Dnaj (Hsp40) Homolog, Subfamily C, Member 30 |
| *DNMT1* | DNA Methyltransferase 1 |
| *DOCK2* | Dock 2 |
| *DPH5* | Diphthamide Hydrolase 5 |
| *DPM3* | Dolichyl Phosphate Mannosyltransferase 3 |
| *DPYD* | Dipeptidyl Peptidase D |
| *DSC3* | Desmocollin 3 |
| *DSE* | Diphthonglipolypeptide Glucosyltransferase |
| *DSG3* | Desmoglein 3 |
| *DUSP18* | Dual Specificity Phosphatase 18 |
| *DUSP7* | Dual Specificity Phosphatase 7 |
| *DYRK2* | Dystrobrevin Kinase 2 |
| *DYRK3* | Dystrobrevin Kinase 3 |
| *E2F7* | E2F Transcription Factor 7 |
| *ECH1* | Echinoderm Homeobox 1 |
| *ECHS1* | Echinocyte Homeobox 1 |
| *ECI2* | Enoyl-CoA Delta Isomerase 2 |
| *ECSIT* | Ecs Intestinal Tumor |
| *EDF1* | Epidermal Growth Factor Receptor Downstream Activator 1 |
| *EGFL6* | Epidermal Growth Factor Like Hexapeptide 6 |
| *EHBP1L1* | Ehler Danlos Syndrome Protein Family Member 1 |
| *EHD2* | Erythroid Histone Deacetylase 2Like 1 |
| *EIF2AK2* | Eukaryotic Translation Initiation Factor 2 Alpha Kinase 2 |
| *EIF2C1* | Eukaryotic Translation Initiation Factor 2 Alpha Subunit Chain 1 |
| *EIF2C3* | Eukaryotic Translation Initiation Factor 2 Alpha Subunit Chain 3 |
| *EIF2C4* | Eukaryotic Translation Initiation Factor 2 Alpha Subunit Chain 4 |
| *EIF4EBP3* | Eukaryotic Translation Initiation Factor 4E Binding Protein 3 |
| *ELF4* | E7 Like Ets Transcription Factor 4 |
| *ELK3* | Ets Transcription Factor 3 |
| *EMP1* | Emerin 1 |
| *EMR2* | Endoplasmic Reticulum Remodeling Protein 2 |
| *ENDOG* | Endoglin |
| *EPHX2* | Eph Domain Containing Protein X2 |
| *ERO1L* | Erythroid Orexin Like Protein 1 |
| *ERP29* | Endoplasmic Reticulum Protein 29 |
| *ESD* | Epidermal Senescence Differentiation Antigen |
| *ETV3* | Etv Homolog 3 |
| *ETV3L* | Etv Homolog 3 Like |
| *EXOC6B* | Exocrine Oxylipins Biosynthesis Regulatory Protein B |
| *EXOSC7* | Exosome Component 7 |
| *EYA3* | Eya Transcriptional Coactivator And Phosphatase 3 |
| *F5* | Factor V |
| *F8* | Factor Viii |
| *FAHD2A* | Fad Dependent Oxidoreductase Domain Containing 2A |
| *FAM105B* | Family With Sequence Similarity 105 Member B |
| *FAM115C* | Family With Sequence Similarity 115 Member C |
| *FAM126A* | Family With Sequence Similarity 126 Member A |
| *FAM165B* | Family With Sequence Similarity 165 Member B |
| *FAM173A* | Family With Sequence Similarity 173 Member A |
| *FAM185A* | Family With Sequence Similarity 185 Member A |
| *FAM195A* | Family With Sequence Similarity 195 Member A |
| *FAM200B* | Family With Sequence Similarity 200 Member B |
| *FAM36A* | Family With Sequence Similarity 36 Member A |
| *FAM55C* | Family With Sequence Similarity 55 Member C |
| *FAM83A* | Family With Sequence Similarity 83 Member A |
| *FAM96A* | Family With Sequence Similarity 96 Member A |
| *FAM96B* | Family With Sequence Similarity 96 Member B |
| *FAP* | Fibroblast Activation Protein |
| *FAT2* | Fat Atypical Cadherin 2 |
| *FAU* | Finkel Biskisreilly Murine Sarcoma Virus (Fbr Musv) Ubiquitously Expressed |
| *FBXL15* | F Box And Leucine Rich Repeat Protein 15 |
| *FCGR2C* | Fc Fragment Of Igg Receptor Iic |
| *FDX1* | Flavodoxin 1 |
| *FDX2* | Flavodoxin 2 |
| *FDXR* | Flavodoxin Reductase |
| *FGD6* | FYVE, RhoGEF And PH Domain Containing 6 |
| *FH* | Fumarate Hydratase |
| *FHIT* | Fragile Histidine Triad |
| *FIS1* | Mitochondrial Fission 1 Homolog |
| *FKBP14* | Fk506 Binding Protein 14 |
| *FKBP15* | Fk506 Binding Protein 15 |
| *FLNA* | Filamin A |
| *FMNL1* | Formin Like Protein 1 |
| *FMNL3* | Formin Like Protein 3 |
| *FN3K* | Fibronectin Type Iii Domain Containing Kinase |
| *FOSL2* | Fos Like 2, Ap 1 Transcription Factor Subunit |
| *FOXJ3* | Forkhead Box J3 |
| *FOXK1* | Forkhead Box K1 |
| *FOXRED1* | Fox Red Scavenger Receptor 1 |
| *FPR1* | Formyl Peptide Receptor 1 |
| *FPR2* | Formyl Peptide Receptor 2 |
| *FPR3* | Formyl Peptide Receptor 3 |
| *FRMD8* | Ferm Domain Containing 8 |
| *FSCN1* | Fascin Actin Bundling Protein 1 |
| *FUT11* | Fucosyltransferase 11 |
| *FXN* | Glutaredoxin |
| *FYB* | Fyn Binding Protein |
| *GADD45GIP1* | Gadd45 Gamma Interacting Protein 1 |
| *GAL3ST4* | Galactose 3 O Sulfotransferase 4 |
| *GALT* | Galactose Aminotransferase |
| *GAPVD1* | Gap Ventral Decussation Guided Protein 1 |
| *GAS2L3* | Growth Arrest Specific 2 Like 3 |
| *GBP6* | Guanylate Binding Protein 6 |
| *GCAT* | Glycine C Acetylase |
| *GCDH* | Glutaryl Coenzyme A Dehydrogenase |
| *GCSH* | Glutathione Synthetase |
| *GFER* | Glioblastoma Expressed Ras Oncogene Homolog |
| *GFOD1* | Glycerol 3 Phosphate Dehydrogenase 1 |
| *GKAP1* | G Kinase Associated Protein 1 |
| *GLI3* | Gli Family Zinc Finger 3 |
| *GLIPR1* | Gli Pathogenesis Related 1 |
| *GLOD4* | Glyoxalase Domain Containing 4 |
| *GLRX5* | Glutaredoxin 5 |
| *GLS* | Glutamine Synthetase |
| *GLT25D1* | Glutaminyl Trna Synthetase 2, Mitochondrial |
| *GLYCTK* | Glycine C Terminal Kinase |
| *GMEB1* | G Protein Coupled Receptor Membrane Protein 1 |
| *GNA15* | Guanine Nucleotide Binding Protein Alpha 15 |
| *GNAI3* | Guanine Nucleotide Binding Protein Alpha I3 |
| *GNB1* | Guanine Nucleotide Binding Protein Beta 1 |
| *GNB4* | Guanine Nucleotide Binding Protein Beta 4 |
| *GON4L* | Gon 4 Like Protein |
| *GOT1* | Glutamicoxaloacetic Transaminase 1 |
| *GOT2* | Glutamicoxaloacetic Transaminase 2 |
| *GPATCH8* | G-Patch Domain Containing 8 |
| *GPR132* | G Protein-Coupled Receptor 132 |
| *GPR141* | G Protein-Coupled Receptor 141 |
| *GPR161* | G Protein-Coupled Receptor 161 |
| *GPR68* | G Protein-Coupled Receptor 68 |
| *GPR84* | G Protein-Coupled Receptor 84 |
| *GPR87* | G Protein-Coupled Receptor 87 |
| *GRHPR* | Glyoxylate And Hydroxypyruvate Reductase |
| *GSDMC* | Gata Zinc Finger Domain Containing 1 |
| *GSTK1* | Gst Killer Toxin Resistance Protein |
| *GSTZ1* | Glutathione S-transferase Zeta 1 |
| *GTF3C4* | General Transcription Factor IIIC Subunit 4 |
| *HACL1* | 2-Hydroxyacyl-CoA Lyase 1 |
| *HADH* | Hydroxyacyl-CoA Dehydrogenase |
| *HADHB* | Hydroxyacyl-CoA Dehydrogenase Trifunctional Multienzyme Complex Subunit Beta |
| *HAGH* | Hydroxyacylglutathione Hydrolase |
| *HAMP* | Hepcidin Antimicrobial Peptide |
| *HAPLN3* | Hyaluronan And Proteoglycan Link Protein 3 |
| *HCAR2* | Hydroxycarboxylic Acid Receptor 2 |
| *HCK* | HCK Proto-Oncogene, Src Family Tyrosine Kinase |
| *HDDC3* | HD Domain Containing 3 |
| *HECA* | Hdc Homolog, Cell Cycle Regulator |
| *HEG1* | Heart Development Protein With EGF Like Domains 1 |
| *HELB* | DNA Helicase B |
| *HEPH* | Hephaestin |
| *HEXIM2* | HEXIM P-TEFb Complex Subunit 2 |
| *HIBADH* | 3-Hydroxyisobutyrate Dehydrogenase |
| *HIGD1A* | HIG1 Hypoxia Inducible Domain Family Member 1A |
| *HIGD2A* | HIG1 Hypoxia Inducible Domain Family Member 2A |
| *HINT1* | Histidine Triad Nucleotide Binding Protein 1 |
| *HINT2* | Histidine Triad Nucleotide Binding Protein 2 |
| *HIVEP1* | Hiv1 Tat Interacting Protein 1 |
| *HIVEP2* | Hiv1 Tat Interacting Protein 2 |
| *HIVEP3* | Hiv1 Tat Interacting Protein 3 |
| *HK2* | Hexokinase 2 |
| *HMGCL* | 3-Hydroxy-3-Methylglutaryl-CoA Lyase |
| *HOXC13* | Homeobox C13 |
| *HPSE* | Heparanase |
| *HRSP12* | Heat Shock Rna Binding Protein 12 |
| *HSCB* | Hemochromatosis Protein H And Beta |
| *HSD17B10* | Hydroxysteroid 17 Beta Dehydrogenase 10 |
| *HSD17B4* | Hydroxysteroid 17 Beta Dehydrogenase 4 |
| *HSD17B8* | Hydroxysteroid 17 Beta Dehydrogenase 8 |
| *HSPA8* | Heat Shock Protein Family A (Hsp70) Member 8 |
| *HSPA9* | Heat Shock Protein Family A (Hsp70) Member 9 |
| *HSPE1* | Heat Shock Protein Family E Member 1 |
| *IBA57* | Inhibitor Of Apoptosis Protein 57 |
| *ICAM1* | Intercellular Adhesion Molecule 1 |
| *IDH1* | Isocitrate Dehydrogenase (NADP(+)) 1 |
| *IDH2* | Isocitrate Dehydrogenase (NADP(+)) 2 |
| *IFFO2* | Intermediate Filament Family Orphan 2 |
| *IFI16* | Interferon Gamma Inducible Protein 16 |
| *IKZF3* | IKAROS Family Zinc Finger 3 |
| *IL12RB2* | Interleukin 12 Receptor Subunit Beta 2 |
| *IL17RA* | Interleukin 17 Receptor A |
| *IL1F5* | Interleukin 1 Family Member 5 |
| *IL1F9* | Interleukin 1 Family Member 9 |
| *IL1RAP* | Interleukin 1 Receptor Accessory Protein |
| *IL20RB* | Interleukin 20 Receptor Subunit Beta |
| *IL21R* | Interleukin 21 Receptor |
| *IL2RA* | Interleukin 2 Receptor Subunit Alpha |
| *IL4R* | Interleukin 4 Receptor |
| *IL7R* | Interleukin 7 Receptor |
| *IMMP1L* | Inner Membrane Protein, Mitochondrial Precursor Like |
| *IMMP2L* | Inner Membrane Protein, Mitochondrial Precursor Like 2 |
| *INHBA* | Inhibin Subunit Alpha |
| *IPMK* | Inositol Polyphosphate Multikinase |
| *IPPK* | Inositol 1,4,5 Trisphosphate 3 Kinase |
| *IQGAP1* | Iq Motif Containing GTPase Activating Protein 1 |
| *IRAK3* | Interleukin 1 Receptor Associated Kinase 3 |
| *ISCA1* | Iron-Sulfur Cluster Assembly Protein 1 |
| *ISCA2* | Iron-Sulfur Cluster Assembly Protein 2 |
| *ISCU* | Iron-Sulfur Cluster Assembly Protein X |
| *ITGA2* | Integrin Subunit Alpha 2 |
| *ITGA5* | Integrin Subunit Alpha 5 |
| *ITGAM* | Integrin Subunit Alpha M |
| *ITGAX* | Integrin Subunit Alpha X |
| *ITPR3* | Inositol 1,4,5-Trisphosphate Receptor Type 3 |
| *ITPRIP* | Inositol 1,4,5-Trisphosphate Receptor Interacting Protein |
| *ITPRIPL2* | Inositol 1,4,5-Trisphosphate Receptor Interacting Protein Like 2 |
| *IVD* | Inositol Dehydrogenase |
| *IVL* | Involucrin |
| *JAGN1* | Jagunal Homolog 1 (Drosophila) |
| *JAK1* | Janus Kinase 1 |
| *JOSD1* | Joubert Syndrome Disruptor 1 |
| *KDM2A* | Lysine Demethylase 2A |
| *KDM5A* | Lysine Demethylase 5A |
| *KDM5B* | Lysine Demethylase 5B |
| *KIAA0090* | Kiaa0090 Gene Product |
| *KIAA0114* | Kiaa0114 Gene Product |
| *KIAA0141* | Kiaa0141 Gene Product |
| *KIAA0226* | Kiaa0226 Gene Product |
| *KIAA0247* | Kiaa0247 Gene Product |
| *KIAA0947* | Kiaa0947 Gene Product |
| *KIAA1432* | Kiaa1432 Gene Product |
| *KIAA1609* | Kiaa1609 Gene Product |
| *KIAA1949* | Kiaa1949 Gene Product |
| *KIF26B* | Kinesin Family Member 26B |
| *KIF2A* | Kinesin Heavy Chain 2A |
| *KIF9* | Kinesin Family Member 9 |
| *KIRREL* | Kin Of Irre Like (Drosophila) |
| *KLC4* | Kinesin Light Chain 4 |
| *KLF15* | Kruppel Like Factor 15 |
| *KLF7* | Kruppel Like Factor 7 |
| *KLHDC2* | Kelch Domain Containing 2 |
| *KLHDC9* | Kelch Domain Containing 9 |
| *KLHL6* | Kelch Like Family Member 6 |
| *KNG1* | Kininogen 1 |
| *KPNA6* | Karyopherin Subunit Alpha 6 |
| *KRT14* | Keratin 14 |
| *KRT16* | Keratin 16 |
| *KRT17* | Keratin 17 |
| *KRT5* | Keratin 5 |
| *KRT6A* | Keratin 6A |
| *KRT6C* | Keratin 6C |
| *LATS1* | Large Tumor Suppressor Kinase 1 |
| *LCN12* | Lipocalin 12 |
| *LDHD* | Lactate Dehydrogenase D |
| *LEAP2* | Liver Expressed Antimicrobial Peptide 2 |
| *LETMD1* | Leucine Zipper And Ef Hand Containing Transmembrane Protein 1 |
| *LHFPL2* | Lipoma Hmgic Fusion Partner Like 2 |
| *LIAS* | Lipoic Acid Synthetase |
| *LILRA6* | Leukocyte Immunoglobulin Like Receptor Subfamily A Member 6 |
| *LILRB3* | Leukocyte Immunoglobulin Like Receptor Subfamily B Member 3 |
| *LIMS1* | LIM Zinc Finger Domain Containing 1 |
| *LIPT1* | Lipid Transfer Protein 1 |
| *LIPT2* | Lipid Transfer Protein 2 |
| *LNPEP* | Leucyl And Cystinyl Aminopeptidase |
| *LOC100128822* | Uncharacterized Loc100128822 |
| *LOC100271836* | Uncharacterized Loc100271836 |
| *LOC154761* | Uncharacterized Loc154761 |
| *LOC202781* | Uncharacterized Loc202781 |
| *LOC284441* | Uncharacterized Loc284441 |
| *LOC388789* | Uncharacterized Loc388789 |
| *LOC440957* | Uncharacterized Loc440957 |
| *LOC728743* | Uncharacterized Loc728743 |
| *LOX* | Lysyl Oxidase |
| *LOXL1* | Lysyl Oxidase Like 1 |
| *LOXL4* | Lysyl Oxidase Like 4 |
| *LRCH3* | Leucine Rich Repeat And Calponin Homology (Chitinase Like) Domain Containing 3 |
| *LRRC8C* | Leucine Rich Repeat Containing 8C |
| *LRRK1* | Leucine Rich Repeat Kinase 1 |
| *LSM3* | Lsm3 Homolog, U6 Small Nuclear Rna And Cytoplasmic Rna Interacting Protein (S. Cerevisiae) |
| *LTB4R* | Leukotriene B4 Receptor |
| *LTB4R2* | Leukotriene B4 Receptor 2 |
| *LUZP1* | Leucine Zipper Protein 1 |
| *LYPD5* | Lysin Motif Containing Protein Family Member 5 |
| *LYRM1* | Lyrin 1 |
| *LYRM5* | Lyrin 5 |
| *MACF1* | Microtubule Actin Crosslinking Factor 1 |
| *MACROD1* | Macrophage Scavenger Receptor 1 Class D Type 1 |
| *MAML1* | Mastermind Like Transcriptional Coactivator 1 |
| *MAP3K2* | Mitogen-Activated Protein Kinase Kinase Kinase 2 |
| *MAP3K6* | Mitogen-Activated Protein Kinase Kinase Kinase 6 |
| *MAP4K5* | Mitogen-Activated Protein Kinase Kinase Kinase 5 |
| *MAP7D1* | Map7 Domain Containing 1 |
| *MARVELD1* | Marvel Domain Containing 1 |
| *MAST4* | Mitochondrial Serine/Threonine Kinase Family member 4 |
| *MBLAC1* | Metallo-Beta-Lactamase Domain Containing 1 |
| *MBNL1* | Muscleblind Like Splicing Regulator 1 |
| *MCAT* | Malate Dehydrogenase, Cytoplasmic |
| *MCEE* | Methylmalonyl Coenzyme A Epimerase |
| *MCL1* | Myeloid Cell Leukemia 1 |
| *MCTP1* | Mitochondrial Carrier Protein 1 |
| *MDH1* | Malate Dehydrogenase 1, Nad (Soluble) |
| *MED13L* | Mediator Complex Subunit 13 Like |
| *MEFV* | Mediterranean Fever |
| *MFSD3* | Major Facilitator Superfamily Domain Containing 3 |
| *MGAT5* | Alpha-1,6-Mannosylglycoprotein 6-Beta-N-Acetylglucosaminyltransferase |
| *MGST2* | Microsomal Glutathione S-Transferase 2 |
| *MICAL2* | Microtubule Associated Monooxygenase, Calponin And LIM Domain Containing 2 |
| *MICALCL* | Microtubule Associated Monooxygenase, Calponin And LIM Domain Containing 2 |
| *MICALL1* | MICAL Like 1 |
| *MICB* | MHC Class I Polypeptide-Related Sequence B |
| *MKKS* | MKKS Centrosomal Shuttling Protein |
| *MKNK1* | MAPK Interacting Serine/Threonine Kinase 1 |
| *MLYCD* | Malonyl Coenzyme A Decarboxylase |
| *MMAB* | Metabolism Of Cobalamin Associated B |
| *MMP1* | Matrix Metallopeptidase 1 |
| *MMP13* | Matrix Metallopeptidase 13 |
| *MMP14* | Matrix Metallopeptidase 14 |
| *MMP3* | Matrix Metallopeptidase 3 |
| *MN1* | Meningioma 1 |
| *MNDA* | Myotonin Protein Kinase |
| *MOBKL2A* | Mob Kinase Activator 1B |
| *MPDU1* | Mannose-P-Dolichol Utilization Defect 1 |
| *MPI* | Mitochondrial Pyruvate Carrier Protein |
| *MPND* | MPN Domain Containing |
| *MRC2* | Mannose Receptor C Type 2 |
| *MRP63* | Multidrug Resistance Protein 63 |
| *MRPL1* | Mitochondrial Ribosomal Protein L1 |
| *MRPL12* | Mitochondrial Ribosomal Protein L12 |
| *MRPL16* | Mitochondrial Ribosomal Protein L16 |
| *MRPL2* | Mitochondrial Ribosomal Protein L2 |
| *MRPL20* | Mitochondrial Ribosomal Protein L20 |
| *MRPL21* | Mitochondrial Ribosomal Protein L21 |
| *MRPL22* | Mitochondrial Ribosomal Protein L22 |
| *MRPL23* | Mitochondrial Ribosomal Protein L23 |
| *MRPL32* | Mitochondrial Ribosomal Protein L32 |
| *MRPL34* | Mitochondrial Ribosomal Protein L34 |
| *MRPL35* | Mitochondrial Ribosomal Protein L35 |
| *MRPL40* | Mitochondrial Ribosomal Protein L40 |
| *MRPL41* | Mitochondrial Ribosomal Protein L41 |
| *MRPL43* | Mitochondrial Ribosomal Protein L43 |
| *MRPL44* | Mitochondrial Ribosomal Protein L44 |
| *MRPL46* | Mitochondrial Ribosomal Protein L46 |
| *MRPL48* | Mitochondrial Ribosomal Protein L48 |
| *MRPL53* | Mitochondrial Ribosomal Protein L53 |
| *MRPL54* | Mitochondrial Ribosomal Protein L54 |
| *MRPS11* | Mitochondrial Ribosomal Protein S11 |
| *MRPS18B* | Mitochondrial Ribosomal Protein S18B |
| *MRPS24* | Mitochondrial Ribosomal Protein S24 |
| *MRPS25* | Mitochondrial Ribosomal Protein S25 |
| *MRPS26* | Mitochondrial Ribosomal Protein S26 |
| *MRPS28* | Mitochondrial Ribosomal Protein S28 |
| *MRPS31* | Mitochondrial Ribosomal Protein S31 |
| *MRPS33* | Mitochondrial Ribosomal Protein S33 |
| *MRPS36* | Mitochondrial Ribosomal Protein S36 |
| *MRPS9* | Mitochondrial Ribosomal Protein S9 |
| *MRTFA* | Myocardin Related Transcription Factor A |
| *MSN* | Moesin |
| *MSRB2* | Methionine Sulfoxide Reductase B2 |
| *MST1* | Macrophage Stimulating 1 |
| *MT1A* | Metallothionein 1A |
| *MT1B* | Metallothionein 1B |
| *MT1E* | Metallothionein 1E |
| *MT1F* | Metallothionein 1F |
| *MT1G* | Metallothionein 1G |
| *MT1H* | Metallothionein 1H |
| *MT1HL1* | Metallothionein 1H Like 1 |
| *MT1X* | Metallothionein 1X |
| *MT2A* | Metallothionein 2A |
| *MT3* | Metallothionein 3 |
| *MT4* | Metallothionein 4 |
| *MTARC1* | Mitochondrial Amidoxime Reducing Component 1 |
| *MT-CO1* | Mitochondrially Encoded Cytochrome C Oxidase I |
| *MT-CO2* | Mitochondrially Encoded Cytochrome C Oxidase II |
| *MT-CO3* | Mitochondrially Encoded Cytochrome C Oxidase III |
| *MTERFD3* | Mterf Domain Containing 3 |
| *MTF1* | Mammalian Target Of Rapamycin Complex 1 (Alpha) |
| *MTFMT* | Mitochondrial Methionyl Trna Formyltransferase |
| *MTHFS* | Methenyl Theonyl Phenylalanine Synthetase |
| *MTIF3* | Mitochondrial Translation Inhibitor Family Member 3 |
| *MX2* | Myxovirus (Influenza Virus) Resistance 2 |
| *MXD1* | Max Dimerization Protein 1 |
| *MYCBP2* | Myc Binding Protein 2 (Estrogen Receptor Associated) |
| *MYEOV2* | Myeloma Overexpressed 2 |
| *MYH9* | Myosin Heavy Chain 9 |
| *MYO1G* | Myocardin Like 1 |
| *MYO9A* | Myosin Ixa |
| *MYO9B* | Myosin Ixb |
| *MYOF* | Myoferlin |
| *MYSM1* | Myb Like, Swirm, And Mpn Domains 1 |
| *N4BP1* | Nucleosome Assembly Protein 1 |
| *NBPF10* | Neurite Enriched Baf60A Family Member 10 |
| *NCF2* | Nuclear And Cellular Functions 2 |
| *NCOA3* | Nucleolar Coordinator 3 |
| *NDE1* | Nucleoporin Depleting Enzyme 1 |
| *NDFIP1* | Ndf Interacting Protein 1 |
| *NDOR1* | NADPH Dependent Diflavin Oxidoreductase 1 |
| *NDUFA1* | Ubiquinone Oxidoreductase Core Subunit A1 |
| *NDUFA10* | Ubiquinone Oxidoreductase Core Subunit A10 |
| *NDUFA11* | Ubiquinone Oxidoreductase Core Subunit A11 |
| *NDUFA12* | Ubiquinone Oxidoreductase Core Subunit A12 |
| *NDUFA13* | Ubiquinone Oxidoreductase Core Subunit A13 |
| *NDUFA2* | Ubiquinone Oxidoreductase Core Subunit A2 |
| *NDUFA3* | Ubiquinone Oxidoreductase Core Subunit A3 |
| *NDUFA4* | Ubiquinone Oxidoreductase Core Subunit A4 |
| *NDUFA5* | Ubiquinone Oxidoreductase Core Subunit A5 |
| *NDUFA6* | Ubiquinone Oxidoreductase Core Subunit A6 |
| *NDUFA7* | Ubiquinone Oxidoreductase Core Subunit A7 |
| *NDUFA8* | Ubiquinone Oxidoreductase Core Subunit A8 |
| *NDUFA9* | Ubiquinone Oxidoreductase Core Subunit A9 |
| *NDUFAB1* | Ubiquinone Oxidoreductase Core Subunit AB1 |
| *NDUFAF1* | Ubiquinone Oxidoreductase Core Subunit AF1 |
| *NDUFAF2* | Ubiquinone Oxidoreductase Core Subunit AF2 |
| *NDUFAF3* | Ubiquinone Oxidoreductase Core Subunit AF3 |
| *NDUFAF4* | Ubiquinone Oxidoreductase Core Subunit AF4 |
| *NDUFB1* | Ubiquinone Oxidoreductase Core Subunit B1 |
| *NDUFB10* | Ubiquinone Oxidoreductase Core Subunit B10 |
| *NDUFB11* | Ubiquinone Oxidoreductase Core Subunit B11 |
| *NDUFB2* | Ubiquinone Oxidoreductase Core Subunit B2 |
| *NDUFB3* | Ubiquinone Oxidoreductase Core Subunit B3 |
| *NDUFB6* | Ubiquinone Oxidoreductase Core Subunit B6 |
| *NDUFB7* | Ubiquinone Oxidoreductase Core Subunit B7 |
| *NDUFB8* | Ubiquinone Oxidoreductase Core Subunit B8 |
| *NDUFC1* | Ubiquinone Oxidoreductase Core Subunit C1 |
| *NDUFC2* | Ubiquinone Oxidoreductase Core Subunit C2 |
| *NDUFS3* | Ubiquinone Oxidoreductase Core Subunit S3 |
| *NDUFS4* | Ubiquinone Oxidoreductase Core Subunit S4 |
| *NDUFS7* | Ubiquinone Oxidoreductase Core Subunit S7 |
| *NDUFS8* | Ubiquinone Oxidoreductase Core Subunit S8 |
| *NDUFV1* | Ubiquinone Oxidoreductase Core Subunit V1 |
| *NDUFV2* | Ubiquinone Oxidoreductase Core Subunit V2 |
| *NDUFV3* | Ubiquinone Oxidoreductase Core Subunit V3 |
| *NECAB3* | N-Terminal EF-Hand Calcium Binding Protein 3 |
| *NFE2L2* | NFE2 Like BZIP Transcription Factor 2 |
| *NFKB1* | Nuclear Factor Kappa B Subunit 1 |
| *NFS1* | NFS1 Cysteine Desulfurase |
| *NFU1* | NFU1 Iron-Sulfur Cluster Scaffold |
| *NHP2* | NHP2 Ribonucleoprotein |
| *NIN* | Niemann Pick Disease Type C1 |
| *NIPBL* | NIPBL Cohesin Loading Factor |
| *NLRC5* | Nod Like Receptor Family Card Member 5 |
| *NLRP1* | Nod Like Receptor Family Card Member 1 |
| *NLRP3* | NLR Family Pyrin Domain Containing 3 |
| *NOD2* | Nucleotide Binding Oligomerization Domain Containing 2 |
| *NOL9* | Nucleolar Protein 9 |
| *NOTCH2* | Notch 2 Transcription Factor |
| *NPC1* | Niemann Pick C1 Protein |
| *NPRL2* | Neuronal Prion Disease Family, Member 2 |
| *NRL* | Nucleolus Redox Regulated Lipoprotein |
| *NSMCE1* | Nsmc Epithelial Protein 1 |
| *NTHL1* | Nithamine Hydrolase Like 1 |
| *NUBP1* | Nuclear Matrix Binding Protein 1 |
| *NUBP2* | Nuclear Matrix Binding Protein 2 |
| *NUBPL* | Nucleobindin 1 |
| *NUDT16L1* | Nudix Hydrolase Domain Containing Protein 16 Like Isoform 1 |
| *NUDT6* | Nudix Hydrolase Domain Containing Protein 6 |
| *NUDT7* | Nudix Hydrolase Domain Containing Protein 7 |
| *NUDT9* | Nudix Hydrolase Domain Containing Protein 9 |
| *NUP153* | Nucleoporin 153Kda |
| *NUP188* | Nucleoporin 188Kda |
| *NUP62* | Nucleoporin 62Kda |
| *OAS2* | 2'-5'-Oligoadenylate Synthetase 2 |
| *OAS3* | 2'-Oligoadenylate Synthetase 3 |
| *OBFC2A* | Obligate Beta Catenin Function Regulator 2A |
| *OCIAD1* | Ovarian Cancer Integrated DNA Repair System Protein A |
| *ODF2L* | Outer Deeply Scattered Domain Containing Protein L |
| *OGDHL* | Oxoglutarate Dehydrogenase L |
| *OGFRL1* | Oxygen Growth Factor Receptor Like 1 |
| *OLFML2B* | Olfactory Transduction Receptor Like 2B |
| *OR5AR1* | Olfactory Receptor Family 5 Subfamily A Member 1 |
| *OSCAR* | Oxygen Saturation Controlling Agent Regulated |
| *OSMR* | Oncostatin M Receptor |
| *OTUD4* | OTU Deubiquitinase 4 |
| *OXNAD1* | Oxidoreductase NAD Binding Domain Containing 1 |
| *OXSM* | 3-Oxoacyl-ACP Synthase, Mitochondrial |
| *P2RY6* | Pyrimidinergic Receptor P2Y6 |
| *PAK2* | Serine/Threonine Protein Kinase Pak2 |
| *PAM* | Peptidylglycine Alpha Amidating Monooxygenase |
| *PANX1* | Pannexin 1 |
| *PAPD7* | Poly(A) RNA polymerase D7 |
| *PARK7* | Parkinsonism Associated Deglycase |
| *PARP14* | Poly (Adp Ribose) Polymerase Family Member 14 |
| *PCBD1* | Polycomb Ring Finger Domain Containing 1 |
| *PCCA* | Propionyl-CoA Carboxylase Subunit Alpha |
| *PCCB* | Propionyl-CoA Carboxylase Subunit Beta |
| *PCYT1A* | Phosphate Cytidylyltransferase 1A, Choline |
| *PCYT2* | Phosphate Cytidylyltransferase 2, Ethanolamine |
| *PDCD1LG2* | Programmed Cell Death Protein Codelet 1 Like G2 |
| *PDHA1* | Pyruvate Dehydrogenase Complex Component A1 |
| *PDHB* | Pyruvate Dehydrogenase Complex Component B |
| *PEBP1* | Phosphoprotein Enrichment Factor Binding Protein 1 |
| *PEMT* | Phosphatidylethanolamine N-Methyltransferase |
| *PEX11G* | Peroxisomal Biogenesis Factor 11G |
| *PEX16* | Peroxisomal Biogenesis Factor 16 |
| *PEX7* | Peroxisomal Biogenesis Factor 7 |
| *PFDN5* | Prefoldin Dnaj Homolog Subfamily C Member 5 |
| *PFKFB3* | 6-Phosphofructo-2-Kinase/Fructose-2,6-Biphosphatase 3 |
| *PGLYRP3* | Polygalacturonase Domain Containing Protein 3 |
| *PGLYRP4* | Polygalacturonase Domain Containing Protein 4 |
| *PHB1* | Prohibitin 1 |
| *PHB2* | Prohibitin 2 |
| *PHPT1* | Phosphohistidine Phosphotransferase 1 |
| *PHYH* | Phytanoyl-CoA 2-Hydroxylase |
| *PIAS3* | Protein Inhibitor Of Activated STAT 3 |
| *PIGH* | Phosphatidylinositol Glycan Anchor Biosynthesis Class H |
| *PIGP* | Phosphatidylinositol Glycan Anchor Biosynthesis Class P |
| *PIK3CA* | Phosphatidylinositol-4,5-Bisphosphate 3-Kinase Catalytic Subunit Alpha |
| *PIK3CG* | Phosphatidylinositol-4,5-Bisphosphate 3-Kinase Catalytic Subunit Gamma |
| *PIN4* | Protein Kinase C Interacting Protein 4 |
| *PKM2* | Pyruvate Kinase M2 |
| *PKP1* | Serine/Threonine Protein Kinase Phosphatase 1 |
| *PLA2G4E* | Protein Pla2G4 Encoding Gene |
| *PLAU* | Plasminogen Activator, Urokinase |
| *PLAUR* | Plasminogen Activator, Urokinase Receptor |
| *PLEC* | Platelet Endothelial Cell Adhesion Molecule |
| *PLEKHJ1* | Pleckstrin Homology Domain Containing J1 |
| *PLEKHM2* | Pleckstrin Homology And RUN Domain Containing M2 |
| *PLK2* | Polo Like Kinase 2 |
| *PLK3* | Polo Like Kinase 3 |
| *PLXNA1* | Plexin A1 |
| *PMAIP1* | Protein Maip1 |
| *PMPCA* | Peptidase, Mitochondrial Processing Subunit Alpha |
| *PMPCB* | Peptidase, Mitochondrial Processing Subunit Beta |
| *PNKD* | PNKD Metallo-Beta-Lactamase Domain Containing |
| *PNPO* | Pyridoxamine 5'-Phosphate Oxidase |
| *PODNL1* | Podoplanin Like 1 |
| *POLDIP2* | Protein O Linked Dipeptidase Family Member 2 |
| *POP5* | POP5 Homolog, Ribonuclease P/MRP Subunit |
| *PPA2* | Inorganic Pyrophosphatase 2 |
| *PPP1R7* | Protein Phosphatase 1 Regulatory Subunit 7 |
| *PPP1R9B* | Protein Phosphatase 1 Regulatory Subunit 9B |
| *PPP4R1* | Protein Phosphatase 4 Regulatory Subunit 1 |
| *PRDM1* | Protein Pr/Set Domain 1 |
| *PRDM2* | Protein Pr/Set Domain 2 |
| *PRDX2* | Protein Arginine Deiminase 2 |
| *PRDX3* | Protein Arginine Deiminase 3 |
| *PRDX5* | Protein Arginine Deiminase 5 |
| *PRKDC* | Protein Kinase D, Catalytic Polypeptide |
| *PRND* | Proline Rich Nuclear Receptor Coactivator 1 |
| *PRNP* | Prion Protein |
| *PSMG4* | Protein Serine Hydroxymethyltransferase 4 |
| *PSTK* | Protein Serine/Threonine Kinase |
| *PTAFR* | Platelet Activating Factor Receptor |
| *PTGR2* | Prostaglandin Reductase 2 |
| *PTHLH* | Parathyroid Hormone Like Hormone |
| *PTOV1* | PTOV1 Extended AT-Hook Containing Adaptor Protein |
| *PTPMT1* | Protein Tyrosine Phosphatase Mitochondrial 1 |
| *PTPN1* | Protein Tyrosine Phosphatase Non-Receptor Type 1 |
| *PTPN12* | Protein Tyrosine Phosphatase, Non-Receptor Type 12 |
| *PTPN14* | Protein Tyrosine Phosphatase, Non-Receptor Type 14 |
| *PTPN22* | Protein Tyrosine Phosphatase, Non-Receptor Type 22 |
| *PTPRE* | Protein Tyrosine Phosphatase Receptor Type E |
| *PTS* | Protein Transport Protein S |
| *QDPR* | Quinoid Dihydropteridine Reductase |
| *QSER1* | Glutamine And Serine Rich 1 |
| *RAB27B* | RAB27B, Member RAS Oncogene Family |
| *RAB31* | RAB31, Member RAS Oncogene Family |
| *RAP2B* | RAP2B, Member Of RAS Oncogene Family |
| *RASA2* | Ras P21 Protein Activator 2 |
| *RASAL2* | Ras P21 Protein Activator Like 2 |
| *RASSF5* | Ras Association Domain Family Member 5 |
| *RBL1* | Recombination Signal Binding Protein For Immunoglobulin Kappa J Region |
| *RBMS1* | RNA Binding Motif Protein 1 |
| *RC3H2* | Ring Finger And Cchc Domain Containing Protein 2 |
| *RCOR1* | Regulator Of Cytokinesis 1 |
| *RECQL* | RecQ Like Helicase |
| *REEP6* | Replication Factor E Subunit P6 |
| *REL* | REL Proto-Oncogene, NF-KB Subunit |
| *RELT* | RELT TNF Receptor |
| *REPIN1* | Replication Initiator Protein Numa Interacting 1 |
| *RHBDF2* | Rhomboid Family Member 2 |
| *RHBDL2* | Rhomboid Family Member 2 Like |
| *RIN2* | Ras And Rab Interactor 2 |
| *RIT1* | Ribosome Maturation Factor Ribosome Inhibitor Complex 1 |
| *RLF* | Replication Factor C Subunit 4 |
| *RNF168* | Ring Finger Protein 168 |
| *RNF169* | Ring Finger Protein 169 |
| *RNF19B* | Ring Finger Protein 19B |
| *RNF213* | Ring Finger Protein 213 |
| *RNF5* | RNA Polymerase Ii Transcription Factor Subunit 5 |
| *ROCK1* | Rho Associated Coiled Coil Containing Protein Kinase 1 |
| *ROMO1* | Romanomerization Domain Containing Protein 1 |
| *RPL14* | Ribosomal Protein L14 |
| *RPL15* | Ribosomal Protein L15 |
| *RPL24* | Ribosomal Protein L24 |
| *RPL26L1* | Ribosomal Protein L26 Like 1 |
| *RPL27A* | Ribosomal Protein L27A |
| *RPL29* | Ribosomal Protein L29 |
| *RPL32* | Ribosomal Protein L32 |
| *RPL34* | Ribosomal Protein L34 |
| *RPL37A* | Ribosomal Protein L37A |
| *RPL6* | Ribosomal Protein L6 |
| *RPP14* | Ribosomal Protein P14 |
| *RPS14* | Ribosomal Protein S14 |
| *RPS19BP1* | Ribosomal Protein S19 Binding Protein 1 |
| *RPS23* | Ribosomal Protein S23 |
| *RPS25* | Ribosomal Protein S25 |
| *RPS27L* | Ribosomal Protein S27 Like |
| *RPSAP9* | Ribosomal Protein Sa Associated Protein 9 |
| *RRAGC* | Ras Related Gtp Binding Protein Rag C |
| *RSAD1* | Radical S Adenosyl Methionine Domain Containing Protein 1 |
| *RSAD2* | Radical S Adenosyl Methionine Domain Containing Protein 2 |
| *RUNDC2A* | Run And Doublecortin Domain Containing Protein 2A |
| *RUNX1* | Runt-Related Transcription Factor 1 |
| *RUNX2* | Runt-Related Transcription Factor 2 |
| *RUNX3* | Runt-Related Transcription Factor 3 |
| *S100A7* | S100 Calcium Binding Protein A7 |
| *S100A8* | S100 Calcium Binding Protein A8 |
| *S100PBP* | S100P Binding Protein |
| *SAMD4B* | Sterile Alpha Motif Domain Containing 4B |
| *SAMD9* | Sterile Alpha Motif Domain Containing 9 |
| *SAMD9L* | Sterile Alpha Motif Domain Containing 9 Like |
| *SAMHD1* | Sterile Alpha Motif And Hd Domain Containing 1 |
| *SAMSN1* | Sam Domain, Sh3 Domain And Nuclear Localization Signals, 1 |
| *SAP18* | Sin3A Associated Protein 18 Kda |
| *SAT2* | Spermidine/Spermine N1-Acetyltransferase Family Member 2 |
| *SBNO2* | Strawberry Notch Homolog 2 (Drosophila) |
| *SBSN* | Suprabasin |
| *SCAND1* | Scan Domain Containing 1 |
| *SCO1* | Synthesis Of Cytochrome C Oxidase 1 |
| *SCRN2* | Secernin 2 |
| *SDHA* | Succinate Dehydrogenase Complex Flavoprotein Subunit A |
| *SDHB* | Succinate Dehydrogenase Complex Iron-Sulfur Subunit B |
| *SDHC* | Succinate Dehydrogenase Complex Subunit C |
| *SDHD* | Succinate Dehydrogenase Complex Subunit D |
| *SDR16C5* | Short Chain Dehydrogenase/Reductase Family 16C Member 5 |
| *SDR39U1* | Short Chain Dehydrogenase/Reductase Family 39U Member 1 |
| *SDSL* | Serine Dehydratase Like |
| *SEC11C* | Protein Transport Protein Sec11C |
| *SEC61B* | Sec61 Translocon Beta Subunit |
| *SELK* | Selenoprotein K |
| *SENP1* | Sumo1/Sentrin Specific Peptidase 1 |
| *SERF2* | Small Edg Related Factor 2 |
| *SERPINB13* | Serpin Family B Member 13 |
| *SERPINB2* | Serpin Family B Member 2 |
| *SERPINB3* | Serpin Family B Member 3 |
| *SERPINB4* | Serpin Family B Member 4 |
| *SERPINB7* | Serpin Family B Member 7 |
| *SERPINB8* | Serpin Family B Member 8 |
| *SERTAD2* | Serta Domain Containing 2 |
| *SERTAD4* | Serta Domain Containing 4 |
| *SFRP2* | Secreted Frizzled Related Protein 2 |
| *SFXN3* | Sideroflexin 3 |
| *SFXN4* | Sideroflexin 4 |
| *SH3GLB1* | Sh3 Domain Containing Glb1 |
| *SH3PXD2A* | Sh3 And Px Domains 2A |
| *SH3PXD2B* | Sh3 And Px Domains 2B |
| *SHBG* | Sex Hormone Binding Protein |
| *SHOX2* | Short Stature Homeobox 2 |
| *SIRPB1* | Signal Regulatory Protein Beta 1 |
| *SIRPB2* | Signal Regulatory Protein Beta 2 |
| *SIRT3* | Sirtuin 3 |
| *SKI* | SKI Proto-Oncogene |
| *SKIL* | SKI Like Proto-Oncogene |
| *SLC10A6* | Solute Carrier Family 10 Member 6 |
| *SLC11A2* | Solute Carrier Family 11 Member A2 |
| *SLC12A6* | Solute Carrier Family 12 Member A6 |
| *SLC16A3* | Solute Carrier Family 16 Member A3 |
| *SLC25A1* | Solute Carrier Family 25 Member A1 |
| *SLC25A11* | Solute Carrier Family 25 Member A11 |
| *SLC25A20* | Solute Carrier Family 25 Member A20 |
| *SLC25A24* | Solute Carrier Family 25 Member A24 |
| *SLC25A26* | Solute Carrier Family 25 Member A26 |
| *SLC25A3* | Solute Carrier Family 25 Member A3 |
| *SLC25A33* | Solute Carrier Family 25 Member A33 |
| *SLC25A38* | Solute Carrier Family 25 Member A38 |
| *SLC25A4* | Solute Carrier Family 25 Member A4 |
| *SLC27A5* | Solute Carrier Family 27 Member A5 |
| *SLC28A3* | Solute Carrier Family 28 Member A3 |
| *SLC31A1* | Solute Carrier Family 31 Member A1 |
| *SLC31A2* | Solute Carrier Family 31 Member A2 |
| *SLC33A1* | Solute Carrier Family 33 Member A1 |
| *SLC35D2* | Solute Carrier Family 35 Member D2 |
| *SLC37A2* | Solute Carrier Family 37 Member A2 |
| *SLC37A4* | Solute Carrier Family 37 Member A4 |
| *SLC43A1* | Solute Carrier Family 43 Member A1 |
| *SLFN11* | Schlafen Family Member 11 |
| *SLFN12L* | Schlafen Family Member 12 Like |
| *SLFN5* | Schlafen Family Member 5 |
| *SMG1* | SMG1 Nonsense Mediated MRNA Decay Associated PI3K Related Kinase |
| *SMURF2* | SMAD Specific E3 Ubiquitin Protein Ligase 2 |
| *SNCA* | Synuclein Alpha |
| *SNCB* | Synuclein Beta |
| *SNHG11* | Small Nucleolar Rna Host Gene 11 |
| *SNHG8* | Small Nucleolar Rna Host Gene 8 |
| *SNRNP25* | Small Nuclear Ribonucleoprotein 25Kda (U11/U12) |
| *SNRNP35* | Small Nuclear Ribonucleoprotein D3 Polypeptide 35Kda |
| *SOD1* | Superoxide Dismutase 1 |
| *SOD2* | Superoxide Dismutase 2 |
| *SOD3* | Superoxide Dismutase 3 |
| *SPAG7* | Spermatogenesis Associated 7 |
| *SPATA24* | Spermatogenesis Associated 24 |
| *SPCS1* | Signal Peptide Peptidase Like 1 |
| *SPCS2* | Signal Peptide Peptidase Like 2 |
| *SPEN* | SPEN Family Transcriptional Repressor |
| *SPHAR* | Spherical Head Domain Containing R |
| *SPHK1* | Sphingosine Kinase 1 |
| *SPRR1B* | Small Proline Rich Protein 1B |
| *SPRR2D* | Small Proline Rich Protein 2D |
| *SPRYD4* | SPRY Domain Containing 4 |
| *SRCAP* | Src Associated Protein |
| *SRGAP2* | Slit Robo Rho GTPase Activating Protein 2 |
| *SRP14* | Signal Recognition Particle Receptor Subunit Beta |
| *SSH1* | Slingshot Protein Phosphatase 1 |
| *SSH2* | Slingshot Protein Phosphatase 2 |
| *ST8SIA4* | ST8 Alpha-N-Acetyl-Neuraminide Alpha-2,8-Sialyltransferase 4 |
| *STAG1* | Stromal Antigen 1 |
| *STAG3L4* | Stromal Antigen 3 Like 4 |
| *STAT1* | Signal Transducer And Activator Of Transcription 1 |
| *STAT2* | Signal Transducer And Activator Of Transcription 2 |
| *STAT3* | Signal Transducer And Activator Of Transcription 3 |
| *STEAP2* | Steap Family Member 2 |
| *STEAP4* | Steap Family Member 4 |
| *STK16* | Serine/Threonine Kinase 16 |
| *STK4* | Serine/Threonine Kinase 4 |
| *STRADB* | STE20 Related Adaptor Beta |
| *STRN* | Striatin |
| *STX11* | Synaptosomal Associated Protein, 25 Kda |
| *STX6* | Synaptosomal Associated Protein, 25 Kda, Type 6 |
| *SUCLG1* | Succinate Coenzyme A Ligase Subunit Beta |
| *SUCLG2* | Succinate Coenzyme A Ligase Subunit Gamma |
| *SULT2A1* | Sulfotransferase Family 2A Member 1 |
| *SUMF1* | Sulfatase Modifying Factor 1 |
| *SURF1* | Surfeit Locus Protein 1 |
| *SWAP70* | Switch-Associated Protein 70 |
| *TACO1* | Translational Activator Of Cytochrome C Oxidase I |
| *TAF1L* | TATA-Box Binding Protein Associated Factor 1 Like |
| *TANC2* | Tetratricopeptide Repeat, Ankyrin Repeat And Coiled-Coil Containing 2 |
| *TAOK1* | TAO Kinase 1 |
| *TAP2* | Transporter 2, ATP Binding Cassette Subfamily B Member |
| *TATDN3* | TatD DNase Domain Containing 3 |
| *TBCA* | Tubulin Beta C Chain |
| *TCEB2* | Translin Related Coding Sequence Binding Factor 2 |
| *TCP11L1* | T Complex Protein 11 Like 1 |
| *TCTA* | Transporter Channel, Transmembrane Domain Containing A |
| *TDGF3* | Tumor Differentially Expressed Gene 3 |
| *TEP1* | Transient Erythropoietic Protein 1 |
| *TET2* | Tetratricopeptide Repeat Domain Containing Protein 2 |
| *TET3* | Tetratricopeptide Repeat Domain Containing Protein 3 |
| *TEX264* | Testis Expressed 264 |
| *TFRC* | Transferrin Receptor |
| *TGFB1* | Transforming Growth Factor, Beta 1 |
| *TGM5* | Transglutaminase 5 |
| *THBD* | Thyroid Hormone Receptor Binding Domain |
| *THBS2* | Thrombospondin 2 |
| *THYN1* | Thymosin 1 |
| *TIMM10* | Translocase Of Inner Mitochondrial Membrane 10 |
| *TIMM13* | Translocase Of Inner Mitochondrial Membrane 13 |
| *TIMM16* | Translocase Of Inner Mitochondrial Membrane 16 |
| *TIMM8B* | Translocase Of Inner Mitochondrial Membrane 8B |
| *TLR1* | Toll Like Receptor 1 |
| *TLR2* | Toll Like Receptor 2 |
| *TLR6* | Toll Like Receptor 6 |
| *TLR8* | Toll Like Receptor 8 |
| *TM4SF19* | Transmembrane 4 L Six Family Member 19 |
| *TM7SF2* | Transmembrane 7 Superfamily Member 2 |
| *TMEM120A* | Transmembrane Protein 120A |
| *TMEM126A* | Transmembrane Protein 126A |
| *TMEM126B* | Transmembrane Protein 126B |
| *TMEM129* | Transmembrane Protein 129 |
| *TMEM14B* | Transmembrane Protein 14B |
| *TMEM14C* | Transmembrane Protein 14C |
| *TMEM154* | Transmembrane Protein 154 |
| *TMEM173* | Transmembrane Protein 173 |
| *TMEM177* | Transmembrane Protein 177 |
| *TMEM184B* | Transmembrane Protein 184B |
| *TMEM199* | Transmembrane Protein 199 |
| *TMEM205* | Transmembrane Protein 205 |
| *TMEM208* | Transmembrane Protein 208 |
| *TMEM223* | Transmembrane Protein 223 |
| *TMEM42* | Transmembrane Protein 42 |
| *TMEM43* | Transmembrane Protein 43 |
| *TMEM45A* | Transmembrane Protein 45A |
| *TMEM93* | Transmembrane Protein 93 |
| *TMOD3* | Transmembrane O Linked Mucin Domain Containing 3 |
| *TMPRSS11D* | Transmembrane Protease, Serine 11D |
| *TNC* | Tenascin C |
| *TNFAIP3* | Traumatic Necrosis Factor Alpha Inducible Protein 3 |
| *TNFAIP6* | Traumatic Necrosis Factor Alpha Inducible Protein 6 |
| *TNFRSF9* | TNF Receptor Superfamily, Member 9 |
| *TNFSF13B* | Tumor Necrosis Factor (Ligand) Superfamily, Member 13B |
| *TNIP3* | T Cell Nuclear Protein 3 |
| *TNNT1* | Troponin T1 |
| *TOPBP1* | Topoisomerase Ii Binding Protein 1 |
| *TP63* | Tumor Protein P63 |
| *TPI1* | Triosephosphate Isomerase 1 |
| *TPM4* | Transcription Factor Pm4 |
| *TPPP2* | Thyroid Peroxidase Related Protein 2 |
| *TRAF3* | Tnf Receptor Associated Factor 3 |
| *TRAM2* | Translocation Associated With Mitosis 2 |
| *TRAPPC2L* | Trap Complex Subunit C2 Like |
| *TRAPPC6A* | Trap Complex Subunit C6A |
| *TREM1* | Tumor Endothelial Marker 1 |
| *TRERF1* | Tre Rna Recognition Motif Containing 1 |
| *TRIO* | Triple Arginine Protein |
| *TRIP12* | Teratocarcinoma Derived Growth Factor 12 |
| *TRPT1* | Translocated Peptidase, Mitochondrial (Ec 3.4.24. |
| *TRPV3* | Capsaicin And Vanilloid Receptor 3 |
| *TRUB2* | Trub Pseudouridine Synthase 2 |
| *TSC22D2* | TSC22 Domain Family Member 2 |
| *TST* | Thiosulfate Transporter |
| *TTC38* | Tripartite Motif Containing Protein 38 |
| *TTLL5* | Tetratricopeptide Repeat Containing Protein 5 |
| *TUBB6* | Tubulin Beta 6 |
| *TUSC2* | Tubulin Beta Specific Chaperone 2 |
| *TXN2* | Thioredoxin Related Antioxidant Protein 2 |
| *TXNL1* | Thioredoxin Like 1 |
| *TXNRD2* | Thioredoxin Reductase 2 |
| *TYMP* | Thymidine Phosphorylase |
| *UBA6* | Ubiquitin Like Modifier Activating Enzyme 6 |
| *UBASH3B* | Ubiquitin Associated And Sh3 Domain Containing B |
| *UBL5* | Ubiquitin Like Modifier Activating Enzyme 5 |
| *UBN1* | Ubiquilin 1 |
| *UBR4* | Ubiquitin Protein Ligase E3 Component N Recognin 4 |
| *UBXN7* | Ubiquilin 7 |
| *UBXN8* | Ubiquilin 8 |
| *UGCG* | Ubiquinol Cytochrome C Reductase Iron Carrier Protein G |
| *UHMK1* | Ubiquitin Like Modifier Dependent Protein Kinase 1 |
| *ULBP2* | Ubiquitin Like Modifier Binding Protein 2 |
| *UNC5B* | Unc-5 Netrin Receptor B |
| *UQCR10* | Ubiquinol-Cytochrome C Reductase, Complex III Subunit X |
| *UQCR11* | Ubiquinol-Cytochrome C Reductase, Complex III Subunit XI |
| *UQCRB* | Ubiquinol-Cytochrome C Reductase Binding Protein |
| *UQCRC1* | Ubiquinol Cytochrome C Reductase Core Protein I |
| *UQCRC2* | Ubiquinol Cytochrome C Reductase Core Protein II |
| *UQCRFS1* | Ubiquinol Cytochrome C Reductase, Iron Sulfur Assembly Factor 1 |
| *UQCRQ* | Ubiquinol Cytochrome C Reductase, Core Protein Q |
| *UROS* | Uroporphyrinogen III Synthase |
| *USP30* | Ubiquitin Specific Peptidase 30 |
| *USP31* | Ubiquitin Specific Peptidase 31 |
| *UXT* | Uridine Diphosphate Glucose Pyrophosphorylase |
| *VCAN* | Vascular Cell Adhesion Molecule 1 |
| *VCL* | Vinculin |
| *VPS28* | VPS28 Subunit Of ESCRT-I |
| *WASF2* | WASP Family Member 2 |
| *WBP1* | WW Domain Binding Protein 1 |
| *WDHD1* | WD Repeat And HMG-Box DNA Binding Protein 1 |
| *WDR47* | WD Repeat Domain 47 |
| *WNK1* | WNK Lysine Deficient Protein Kinase 1 |
| *WNT9A* | Wnt Family Member 9A |
| *WWC3* | WWC Family Member 3 |
| *XIAP* | X-Linked Inhibitor Of Apoptosis |
| *XIRP1* | Xin Actin-Binding Repeat-Containing Protein 1 |
| *XRN1* | 5'-3' Exoribonuclease 1 |
| *YEATS2* | Yeats Domain Containing 2 |
| *ZC3HAV1L* | Zinc Finger Ccch-Type, Antiviral 1 Like |
| *ZNF185* | Zinc Finger Protein 185 |
| *ZNF267* | Zinc Finger Protein 267 |
| *ZNF444* | Zinc Finger Protein 444 |
| *ZNF469* | Zinc Finger Protein 469 |
| *ZNF511* | Zinc Finger Protein 511 |
| *ZNF563* | Zinc Finger Protein 563 |
| *ZNF699* | Zinc Finger Protein 699 |
| *ZNFX1* | Zinc Finger Nfx1-Type Containing 1 |
| *ZNHIT1* | Zinc Finger, Hit-Type Containing 1 |
| *ZSCAN20* | Zinc Finger And Scan Domain Containing 20 |
| *ZSWIM4* | Zinc Finger Swim-Type Containing 4 |
